# The Burden and Trends of Degenerative Mitral Valve Disease at the Global, Regional, and National Levels From 1990 to 2021, With Projections to 2035

**DOI:** 10.5334/gh.1489

**Published:** 2025-10-28

**Authors:** Qiang Li, Yifan Yang, Zhi-Nan Lu, Xunan Guo, Xinmin Liu, Zhengming Jiang, Wenhui Wu, Chengqian Yin, Jianxin Li, Xiangfeng Lu, Guangyuan Song

**Affiliations:** 1 Interventional Center of Valvular Heart Disease, Beijing Anzhen Hospital, Capital Medical University, Beijing, China; 2 Department of Epidemiology, Fuwai Hospital, National Center for Cardiovascular Diseases, Chinese Academy of Medical Sciences and Peking Union Medical College, Beijing, China

**Keywords:** Degenerative mitral valve disease, Global burden of disease, Prevalence, Mortality, Disability-adjusted life years

## Abstract

**Background:**

Degenerative mitral valve disease (DMVD) is a significant contributor to the global burden of disease. This study aimed to estimate the prevalence, mortality, and disability-adjusted life years (DALYs) rates of DMVD at global, regional, and national levels from 1990 to 2021 and to project its future burden.

**Methods:**

This study extracted three pivotal indicators, including the prevalence, mortality, and DALYs related to DMVD, from the Global Burden of Disease 2021. The average annual percentage change and rate change were utilized to evaluate the changes in the disease burden. Decomposition analyses were conducted to evaluate these changes. In addition, a Bayesian age-period-cohort analysis was performed to forecast the future burden of DMVD.

**Results:**

In 2021, the global age-standardized prevalence rates (ASPRs), age-standardized mortality rates (ASMRs), and age-standardized disability-adjusted life year rates (ASDRs) for DMVD were 182.13 per 100,000 persons [95% uncertainty interval (UI): 169.952, 196.070], 0.456 per 100,000 persons (95% UI: 0.394, 0.514), and 11.362 per 100,000 persons (95% UI: 9.867, 13.611), respectively. Regions with a high sociodemographic index exhibited the most substantial disease burden. Women exhibited lower ASPR than men, but higher ASMR. Decomposition analyses reveal that improvements in DMVD burden were primarily attributable to epidemiological changes; however, it was negatively affected by population growth and aging. Predictive analysis suggests that global projections for DMVD in 2035 estimate approximately 21.41 million (95% UI: 15,718,776, 27,102,848) cases of prevalence, 47,878 (95% UI: 28,449, 67,307) cases of mortality, and 1.20 million (95% UI: 793,487, 1,615,972) cases of DALYs.

**Conclusions:**

The global burden of DMVD, indicated in its age-standardized prevalence, mortality, and DALYs rates, exhibits significant declines. However, significant regional and national variations exist. Findings of our study emphasize the importance of devising targeted public health strategies tailored to different regions, countries, and populations, with the aim of further mitigating DMVD’s global impact.

## Graphical Abstract

**Figure:**
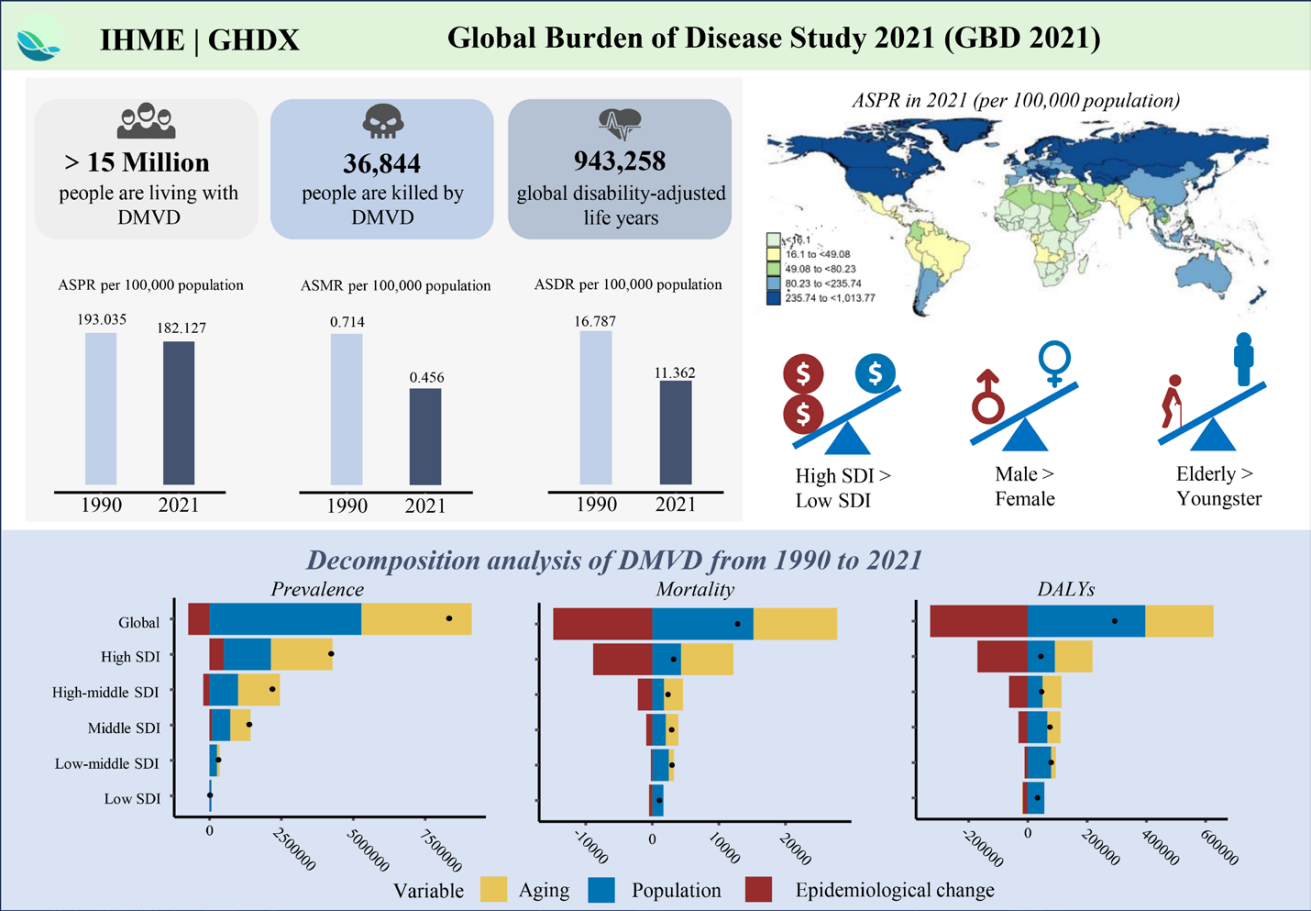

## Introduction

Degenerative mitral valve disease (DMVD), one of the most common types of valvular heart disease (VHD), is increasingly contributing to mortality, loss of quality of life, and rising healthcare costs as the global population ages (1,2,3). It is a series of diseases characterized by anatomical defects and morphological changes in the mitral valve, resulting in compromised mitral valve function (4). Morphologic changes of the mitral valve in DMVD include leaflet thickening, tendon lengthening, tendon rupture, and annulus dilatation (5,6). The pathogenesis primarily involves degeneration and dysfunction of the valve structure, with the principal pathological types comprising mucoid degeneration, lack of elastic fibers, and calcific lesions (7,8,9). DMVD is estimated to affect at least 24 million people globally, resulting in 34,000 deaths and 880,000 disability-adjusted life years (DALYs) in 2019 (2).

In contrast to rheumatic heart disease, non-rheumatic DMVD is associated with aging, genetic predisposition, metabolic disorders, and other factors (10). Because of improved living conditions and the discovery of penicillin, rheumatic valvular disease is effectively managed in developed countries, whereas the degenerative disease has emerged as the leading cause of heart valve disease (11). In addition, advancements in cardiac ultrasound, surgery, and transcatheter valve repair or replacement have reduced the mortality rate of DMVD in some regions, especially in developed countries. However, due to medical resources and valve surgery techniques, the treatment of heart valve disease in developing countries faces significant challenges. The prevalence of COVID-19 in recent years has further exacerbated these inequalities.

A comprehensive insight into the DMVD disease burden globally and regionally is essential for the formulation of targeted interventions and health strategies worldwide. However, there is currently a lack of evidence for updated and expansive data on the global burden and trends associated with DMVD. This study therefore aimed to assess the current status of the disease burden, evolving trends, and inequalities of DMVD between 1990 and 2021 across global, regional, and national levels using data from the Global Burden of Disease (GBD) 2021.

## Method

### Data acquisition and burden description

This study is a secondary analysis based on the GBD 2021. The data on DMVD in this study were obtained from the GBD 2021, which offers a comprehensive analysis of 369 diseases and 88 risk factors in 204 countries and territories (12,13,14). DMVD was identified using the International Classification of Diseases, 10th revision codes I34 to I34.9. From GBD 2021, we extracted data on patients with DMVD from 1990 to 2021, including prevalence, mortality, and DALYs, with their 95% uncertainty interval (UI), categorized by region, country, age, and sex. All data in this study are available at https://vizhub.healthdata.org/gbd-results.

DALYs are a metric for assessing health losses from non-fatal and fatal outcomes, which are calculated as the sum of years of life lost (YLLs) and years lived with disability (YLDs) (12). YLLs were defined as the standardized life expectancy multiplied by the standard life expectancy at age of death. Therefore, this metric reflects premature death due to disease. YLDs are calculated from disease sequelae, which is defined as the prevalence multiplied by the disability weights (DWs) of the health condition associated with that sequela. DWs are measured on a scale of 0–1, where 0 indicates a status equivalent to full health and 1 indicates a status equivalent to death. Under the assumption that the uncertainty in the YLLs is independent of the uncertainty in the YLDs, 1,000 repetitive samplings were performed for YLLs and YLDs. The 1,000 draws for DALYs were calculated by summing the first of the 1,000 draws for YLLs and YLDs and then repeating the process for each subsequent draw. The 95% UIs are expressed by using the 25th and 975th results of the DALYs uncertainty distribution. The core equations can be written as follows:


\[
\begin{array}{l}
YLL = \sum\nolimits_{c = 1, a = 0, s = 1}^{\infty} d_{cas} e_{a},\\
YLD\ Rate_{k} = \frac{\sum\nolimits_{l = 1}^{n} ADW_{lk}} {n},\\
DALYs = YLLs + YLDs,
\end{array}
\]

where *ADW_ik_* represents attributable DW for disease sequela *k* in simulant l. The actual number of YLDs from disease sequela *k* in an age-sex-location-year is then computed as the YLD rate *k* times the appropriate age-sex-location-year population.

In this study, the rates of prevalence, mortality, and DALYs were standardized using the global age structure in 2021, which makes the burden of disease comparable across regions and countries by standardizing age demographics. The estimates account for random and non-random error components, reflected in the 95% UI.

### Statistical analysis

This study offers a descriptive analysis of DMVD. We compared the prevalence, mortality, and DALYs for DMVD across regions, countries, age groups, and sex. The joinpoint regression analysis model was employed to reveal examine relative changes in the age-standardized rates of prevalence, mortality, and DALYs over the past 31 years, from 1990 to 2021. This approach has been widely used in the epidemiological analysis of chronic diseases such as cardiovascular diseases (15). The epidemiological data were first log-transformed, and a grid search was employed to segment the data, selecting the grid points with the smallest mean squared error as joinpoints. The optimal number of joinpoints for the Joinpoint regression model was determined using the Monte Carlo permutation test. The maximum number of joinpoints was set to 5 and the minimum number of potential joinpoints to 0. The average rate of change of DMVD from 1990 to 2021 was represented by the average annual percentage change (AAPC) and its 95% confidence intervals (CIs), which were calculated based on the t-distribution. The analysis of the results depends on the 95% CI of the AAPC. If the lower limit of the 95% CI is >0 (or the upper limit is <0), the indicator is considered to be trending upward (or downward); otherwise, the change is deemed non-statistically significant. For changes in the burden of disease within age groups, the rate change (RC) was used, which is defined as follows:


\[
RC\ =\ {rate_{2021}}\ -\ {rate_{1990}}.
\]

To explore the factors influencing changes in the prevalence, mortality, and DALYs of DMVD, we performed a decomposition analysis. This analysis is a quantitative method used to identify the factors that cause changes in an indicator, by decomposing the overall differences into three components: aging, population growth, and epidemiological changes (16). Epidemiological changes refer to changes in prevalence, mortality, and DALYs, which means the contribution of changes in rates across age groups to the overall differences. Aging refers to the contribution of changes in the proportion (composition ratio) of the population in each age group to the overall differences. Population growth refers to the contribution of changes in the total population to the overall differences. All these indicators were obtained by comparing data from 1990 to 2021. For specific calculation methods, please refer to the Supplementary Material.

The global disease burden of DMVD was projected up to 2035. A Bayesian age-period-cohort analysis was employed to forecast global trends in prevalence, mortality, and DALYs for DMVD from 2022 to 2035. This model employs generalized linear models to link disease indicators with population age structure and size (17) and has been widely applied and validated in epidemiological forecasting (18,19). In our study, we hypothesized that the age-specific and period-specific death counts *N_ij_* follow a Poisson distribution, *N_ij_* ∼ Pois(*µ_ij_*), and applied a model-specific link function within the Bayesian framework. The regression of age- and period-specific death counts was performed against age, period, and cohort effects, with the corresponding populations used as offsets. The formula is as follows:


\[
g\left({\frac{{{\mu _{ij}}}}{{{M_{ij}}}}} \right)\; = \;{a_i}\;+\;{\beta _j}\;+\;{\gamma _k},
\]

where *µ_ij_* represents period-specific death counts; 𝛼*_i_*, 𝛽*_j_*, and 𝛾*_k_* (*k* = *I* – *i* + *j*) represent the effects of age, period, and cohort, respectively; and *N_ij_* and *M_ij_* denote the age-specific and period-specific death counts and population, respectively.

The data were estimated and presented as both numerical counts and age-standardized rates per 100,000 persons of the population, along with 95% UIs. R software (version 4.3.3) was used for all analyses and graphical representations.

## Results

### Global level

From 1990 to 2021, the global age-standardized rates of prevalence, mortality, and DALYs for DMVD exhibited downward trends. In 2021, there were 15,494,647 cases of DMVD (95% UI: 14,457,324 to 16,702,738), with the age-standardized prevalence rates (ASPRs) decreasing from 193.035 per 100,000 people (95% UI: 178.737 to 208.374) in 1990 to 182.13 per 100,000 people (95% UI: 169.952 to 196.070) in 2021, reflecting an AAPC of –0.19% (95% CI: –0.21 to –0.16) (Table 1). The number of mortalities was 36,844 (95% UI: 31,883 to 41,572), with an age-standardized mortality rate (ASMR) of 0.456 per 100,000 people (95% UI: 0.394 to 0.514), indicating an AAPC of –1.44% (95% CI: –1.54 to –1.35) from 1990 to 2021 (Table 2). In 2021, the global DALYs for DMVD were 943,258 (95% UI: 818,239 to 1,134,001), with an age-standardized disability-adjusted life year rate (ASDR) of 11.362 per 100,000 people (95% UI: 9.867 to 13.611), representing an AAPC of –1.26% (95% CI: –1.32 to –1.20) from 1990 to 2021 (Table 3).

**Table 1:** The age-standardized prevalence rates and average annual percentage change for DMVD, 1990–2021.


	1990		2021		1990–2021
		
	CASES (95% UI)	AGE-STANDARDIZED PREVALENCE PER 100,000 POPULATION (95% UI)	CASES (95% UI)	AGE- STANDARDIZED PREVALENCE PER 100,000 POPULATION (95% UI)	AAPC % (95% CI)

Global	7,111,757 (6,572,737 to 7,719,089)	193.035 (178.737 to 208.374)	15,494,647 (14,457,324 to 167,02,738)	182.127 (169.952 to 196.070)	–0.19 (–0.21 to –0.16)

**Sex**					

Women	2,787,243 (2,574,278 to 3,004,423)	134.526 (124.141 to 144.824)	5,665,310 (5,281,762 to 6,073,695)	121.110 (112.919 to 129.824)	–0.33 (–0.36 to –0.30)

Men	4,324,515 (3,989,733 to 4,706,437)	272.362 (252.434 to 294.906)	9,829,338 (9,179,556 to 10,615,234)	257.917 (240.958 to 278.205)	–0.18 (–0.20 to –0.15)

**SDI**					

High SDI	3,870,206 (3,585,779 to 4,185,413)	337.886 (313.216 to 365.835)	8,149,325 (7,632,524 to 8,750,587)	364.237 (341.570 to 390.641)	0.26 (0.21 to 0.31)

High-middle SDI	2,248,063 (2,073,439 to 2,447,323)	235.909 (218.199 to 256.065)	4,486,461 (4,187,532 to 4,821,465)	223.553 (208.801 to 240.007)	–0.17 (–0.21 to –0.13)

Middle SDI	699,387 (632,990 to 774,419)	77.365 (70.345 to 85.015)	2,131,567 (1,978,434 to 2,317,641)	83.143 (77.415 to 89.931)	0.23 (0.20 to 0.25)

Low-middle SDI	236,184 (206,976 to 267,733)	44.255 (38.824 to 49.939)	597,241 (530,959 to 673,732)	44.667 (39.907 to 50.046)	0.04 (0.02 to 0.06)

Low SDI	49,418 (42,745 to 56,717)	24.133 (21.034 to 27.411)	115,513 (101,726 to 130,949)	24.772 (21.958 to 27.948)	0.10 (0.04 to 0.15)

**Region**					

Andean Latin America	5,382 (4,251 to 6,683)	27.279 (21.584 to 33.783)	16,250 (13,104 to 19,888)	27.857 (22.476 to 34.064)	0.07 (–0.15 to 0.29)

Australasia	49,743 (43,137 to 58,430)	202.344 (175.247 to 237.567)	115,502 (98,356 to 139,123)	200.848 (171.779 to 241.187)	–0.03 (–0.09 to 0.03)

Caribbean	9,532 (7,702 to 11,614)	36.710 (29.682 to 45.003)	19,885 (16,459 to 23,915)	36.943 (30.607 to 44.372)	0.04 (0.01 to 0.07)

Central Asia	164,483 (131,623 to 206,939)	376.903 (302.355 to 471.548)	360,193 (302,400 to 433,574)	496.926 (420.359 to 589.812)	0.90 (0.85 to 0.94)

Central Europe	395,349 (336,868 to 461,279)	264.609 (226.754 to 306.170)	678,553 (594,742 to 782,443)	291.325 (254.897 to 334.975)	0.32 (0.26 to 0.37)

Central Latin America	28,266 (24,458 to 32,552)	35.812 (31.179 to 41.249)	91,700 (80,539 to 104,481)	37.254 (32.807 to 42.377)	0.13 (0.06 to 0.20)

Central Sub-Saharan Africa	3,373 (2,633 to 4,211)	15.761 (12.525 to 19.432)	7,308 (5,802 to 8,967)	13.729 (10.910 to 16.744)	–0.43 (–0.45 to –0.42)

East Asia	835,228 (778,575 to 897,822)	111.551 (104.349 to 119.286)	2,697,966 (2,558,787 to 2,843,604)	124.226 (117.689 to 130.923)	0.33 (0.24 to 0.42)

Eastern Europe	653,909 (597,345 to 719,723)	236.319 (216.719 to 258.113)	936,899 (862,626 to 1,026,287)	258.753 (238.933 to 282.708)	0.29 (0.27 to 0.32)

Eastern Sub-Saharan Africa	10,727 (8,952 to 12,602)	14.488 (12.223 to 16.989)	21,886 (18,404 to 25,729)	13.002 (11.045 to 15.162)	–0.34 (–0.36 to –0.32)

High-income Asia Pacific	757,805 (701,584 to 824,389)	380.346 (352.856 to 411.724)	1,938,173 (1,818,157 to 2,065,322)	390.613 (366.971 to 418.011)	0.09 (0.07 to 0.11)

High-income North America	1,942,737 (1,829,074 to 2,071,000)	521.738 (490.575 to 556.915)	3,932,341 (3,732,847 to 4,161,755)	562.804 (535.226 to 594.686)	0.27 (0.23 to 0.30)

North Africa and Middle East	102,905 (85,688 to 121,454)	68.010 (56.753 to 80.588)	270,184 (230,370 to 317,865)	65.495 (56.181 to 76.561)	–0.11 (–0.19 to –0.03)

Oceania	1,678 (1,308 to 2,126)	72.248 (57.307 to 90.609)	4,262 (3,407 to 5,357)	71.210 (57.797 to 88.533)	–0.04 (–0.08 to 0)

South Asia	166,082 (150,544 to 182,992)	33.711 (30.837 to 36.734)	491,176 (447,265 to 539,291)	35.668 (32.656 to 38.802)	0.20 (0.11 to 0.29)

Southeast Asia	159,232 (137,821 to 184,629)	73.668 (63.834 to 84.867)	493,226 (428,684 to 566,120)	83.990 (73.573 to 96.394)	0.43 (0.41 to 0.44)

Southern Latin America	61,105 (50,073 to 74,682)	132.441 (108.732 to 161.925)	123,475 (102,958 to 152,017)	138.055 (114.773 to 169.687)	0.11 (0.05 to 0.16)

Southern Sub-Saharan Africa	4,034 (3,615 to 4,494)	14.145 (12.728 to 15.804)	7,598 (6,779 to 8,536)	12.569 (11.309 to 14.074)	–0.38 (–0.44 to –0.32)

Tropical Latin America	40,304 (37,047 to 43,713)	45.018 (41.414 to 48.830)	111,746 (103,635 to 120,984)	43.749 (40.642 to 47.259)	–0.08 (–0.17 to 0.01)

Western Europe	1,707,798 (1,578,748 to 1,854,639)	278.086 (257.079 to 302.185)	3,148,081 (2,910,257 to 3,407,229)	311.295 (287.651 to 337.336)	0.36 (0.33 to 0.39)

Western Sub-Saharan Africa	12,087 (10,446 to 13,993)	13.222 (11.490 to 15.355)	28,243 (24,381 to 32,739)	13.141 (11.344 to 15.333)	–0.02 (–0.07 to 0.03)


Abbreviations: AAPC, average annual percentage change; CI, confidence interval; DMVD, degenerative mitral valve disease; SDI, sociodemographic index; UI, uncertainty interval.

**Table 2:** The age-standardized mortality rates and average annual percentage change for DMVD, 1990–2021.


	1990		2021		1990–2021
		
	CASES (95% UI)	AGE-STANDARDIZED MORTALITY PER 100,000 POPULATION (95% UI)	CASES (95% UI)	AGE-STANDARDIZED MORTALITY PER 100,000 POPULATION (95% UI)	AAPC % (95% CI)

Global	23,954 (21,032 to 26,296)	0.714 (0.627 to 0.778)	36,844 (31,883 to 41,572)	0.456 (0.394 to 0.514)	–1.44 (–1.54 to –1.35)

**Sex**					

Women	15,396 (13,094 to 17,330)	0.793 (0.674 to 0.891)	22,675 (18,541 to 26,629)	0.487 (0.398 to 0.573)	–1.58 (–1.70 to –1.45)

Men	8,558 (7,513 to 9,479)	0.586 (0.514 to 0.640)	14,168 (12,368 to 16,111)	0.408 (0.357 to 0.459)	–1.16 (–1.28 to –1.04)

**SDI**					

High SDI	13,007 (11,846 to 13,785)	1.181 (1.071 to 1.255)	16,264 (13,478 to 17,843)	0.646 (0.548 to 0.702)	–1.95 (–2.19 to –1.70)

High-middle SDI	4,443 (3,962 to 4,833)	0.512 (0.454 to 0.555)	6,887 (6,041 to 7,711)	0.365 (0.319 to 0.409)	–1.05 (–1.23 to –0.86)

Middle SDI	2,706 (2,210 to 3,196)	0.282 (0.225 to 0.340)	5,692 (4,775 to 7,097)	0.230 (0.191 to 0.288)	–0.7 (–0.95 to –0.44)

Low-middle SDI	2,430 (1,707 to 3,236)	0.415 (0.287 to 0.555)	5,465 (4,214 to 6,975)	0.405 (0.310 to 0.526)	–0.06 (–0.34 to 0.22)

Low SDI	1,336 (776 to 1,875)	0.621 (0.378 to 0.910)	2,479 (1,698 to 3,446)	0.510 (0.344 to 0.729)	–0.61 (–0.71 to –0.50)

**Region**					

Andean Latin America	62 (48 to 73)	0.274 (0.208 to 0.325)	120 (89 to 148)	0.201 (0.148 to 0.247)	–1.02 (–1.98 to –0.04)

Australasia	251 (226 to 269)	1.127 (1.007 to 1.206)	361 (302 to 408)	0.585 (0.496 to 0.657)	–2.14 (–2.65 to –1.62)

Caribbean	130 (109 to 155)	0.483 (0.407 to 0.564)	244 (206 to 285)	0.457 (0.385 to 0.537)	–0.13 (–0.56 to 0.31)

Central Asia	35 (29 to 41)	0.076 (0.062 to 0.091)	214 (189 to 239)	0.295 (0.260 to 0.330)	4.21 (2.58 to 5.87)

Central Europe	1,221 (1,121 to 1,351)	0.858 (0.786 to 0.957)	2,432 (2,165 to 2,649)	1.048 (0.935 to 1.140)	0.59 (0.21 to 0.96)

Central Latin America	361 (347 to 374)	0.444 (0.422 to 0.461)	1,032 (896 to 1,184)	0.422 (0.366 to 0.484)	–0.38 (–1.58 to 0.84)

Central Sub-Saharan Africa	168 (90 to 246)	0.836 (0.458 to 1.300)	354 (207 to 547)	0.732 (0.443 to 1.206)	–0.42 (–0.49 to –0.35)

East Asia	956 (559 to 1,205)	0.141 (0.082 to 0.180)	1,101 (821 to 1,450)	0.058 (0.043 to 0.077)	–2.79 (–3.66 to –1.91)

Eastern Europe	153 (146 to 160)	0.059 (0.056 to 0.062)	758 (699 to 815)	0.224 (0.207 to 0.241)	4.65 (3.79 to 5.52)

Eastern Sub-Saharan Africa	535 (282 to 751)	0.711 (0.393 to 1.068)	858 (557 to 1,245)	0.503 (0.313 to 0.789)	–1.10 (–1.17 to –1.03)

High-income Asia Pacific	2,621 (2,363 to 2,768)	1.503 (1.334 to 1.598)	3,908 (2,905 to 4,493)	0.539 (0.417 to 0.610)	–3.34 (–3.64 to –3.03)

High-income North America	3,651 (3,289 to 3,844)	1.000 (0.903 to 1.053)	4,204 (3,540 to 4,558)	0.590 (0.506 to 0.635)	–1.73 (–2.27 to –1.19)

North Africa and Middle East	1,924 (1,459 to 2,579)	1.137 (0.857 to 1.516)	3,243 (2,548 to 4,606)	0.751 (0.592 to 1.076)	–1.34 (–1.45 to –1.22)

Oceania	4 (2 to 7)	0.066 (0.036 to 0.113)	6 (3 to 10)	0.044 (0.027 to 0.080)	–1.29 (–1.61 to –0.97)

South Asia	1,824 (1,031 to 2,629)	0.345 (0.196 to 0.498)	4,845 (3,212 to 6,326)	0.361 (0.238 to 0.475)	0.20 (–0.28 to 0.68)

Southeast Asia	219 (142 to 535)	0.096 (0.060 to 0.260)	575 (399 to 1,222)	0.104 (0.070 to 0.228)	0.19 (0.02 to 0.36)

Southern Latin America	249 (221 to 273)	0.566 (0.501 to 0.622)	329 (296 to 355)	0.368 (0.331 to 0.396)	–1.14 (–1.99 to –0.29)

Southern Sub-Saharan Africa	142 (87 to 169)	0.439 (0.257 to 0.539)	236 (167 to 300)	0.399 (0.277 to 0.516)	–0.35 (–0.67 to –0.03)

Tropical Latin America	786 (756 to 810)	0.826 (0.784 to 0.858)	1,702 (1,563 to 1,803)	0.674 (0.617 to 0.715)	–0.55 (–1.10 to 0.01)

Western Europe	8,031 (7,349 to 8,561)	1.357 (1.241 to 1.450)	9,302 (7,674 to 10,174)	0.781 (0.659 to 0.846)	–1.75 (–2.08 to –1.41)

Western Sub-Saharan Africa	628 (356 to 915)	0.778 (0.447 to 1.192)	1,021 (623 to 1,558)	0.561 (0.345 to 0.879)	–1.05 (–1.15 to –0.95)


Abbreviations: AAPC, average annual percentage change; CI, confidence interval; DMVD, degenerative mitral valve disease; SDI, sociodemographic index; UI, uncertainty interval.

**Table 3:** The age-standardized DALY rates and average annual percentage change for DMVD, 1990–2021.


	1990		2021		1990– 2021
		
	CASES (95% UI)	AGE-STANDARDIZED DALYS PER 100,000 POPULATION (95% UI)	CASES (95% UI)	AGE- STANDARDIZED DALYS PER 100,000 POPULATION (95% UI)	AAPC % (95% CI)

Global	645,877 (553,701 to 749,210)	16.787 (14.677 to 19.281)	943,258 (818,239 to 1,134,001)	11.362 (9.867 to 13.611)	–1.26 (–1.32 to –1.20)

**Sex**					

Women	374,581 (312,335 to 443,051)	17.622 (14.910 to 20.606)	509,344 (414,981 to 614,453)	11.278 (9.162 to 13.637)	–1.44 (–1.54 to –1.35)

Men	271,296 (230,849 to 319,851)	15.869 (13.324 to 18.935)	433,914 (358,440 to 527,231)	11.573 (9.657 to 14.071)	–1.01 (–1.10 to –0.92)

**SDI**					

High SDI	286,597 (261,114 to 323,954)	25.977 (23.762 to 29.184)	334,695 (286,438 to 404,530)	15.008 (13.019 to 17.984)	–1.75 (–1.91 to –1.60)

High-middle SDI	134,087 (115,261 to 158,668)	14.093 (12.165 to 16.728)	184,649 (156,704 to 228,858)	9.770 (8.331 to 12.006)	–1.16 (–1.28 to –1.05)

Middle SDI	97,118 (81,912 to 113,664)	8.331 (6.990 to 9.751)	175,874 (152,861 to 212,348)	6.761 (5.851 to 8.188)	–0.69 (–0.86 to –0.52)

Low-middle SDI	82,288 (57,207 to 112,961)	11.368 (8.181 to 15.040)	165,113 (129,856 to 204,532)	10.623 (8.406 to 13.210)	–0.21 (–0.33 to –0.10)

Low SDI	44,878 (23,753 to 63,997)	16.168 (9.410 to 22.691)	81,577 (56,425 to 109,180)	12.799 (8.997 to 17.540)	–0.74 (–0.83 to –0.64)

**Region**					

Andean Latin America	2,302 (1,784 to 2,735)	8.522 (6.626 to 10.131)	3,687 (2,789 to 4,541)	5.870 (4.449 to 7.227)	–1.00 (–1.93 to –0.05)

Australasia	5,267 (4,866 to 5,791)	22.836 (21.096 to 25.001)	6,561 (5,674 to 7,551)	11.823 (10.379 to 13.455)	–2.17 (–2.60 to –1.73)

Caribbean	4,558 (3,716 to 5,540)	15.470 (12.765 to 18.507)	7,557 (6,179 to 9,098)	14.462 (11.763 to 17.451)	–0.13 (–0.52 to 0.26)

Central Asia	3,378 (2,296 to 5,095)	7.740 (5.118 to 11.783)	10,234 (7,974 to 13,440)	14.090 (10.694 to 18.953)	1.87 (1.34 to 2.40)

Central Europe	33,650 (30,189 to 38,167)	22.953 (20.587 to 26.139)	53,614 (47,716 to 61,177)	24.507 (21.976 to 27.784)	0.12 (–0.19 to 0.44)

Central Latin America	11,018 (10,612 to 11,464)	11.323 (10.842 to 11.868)	26,377 (23,036 to 30,245)	10.396 (9.097 to 11.917)	–0.46 (–1.30 to 0.39)

Central Sub-Saharan Africa	5,445 (2,782 to 7,847)	20.342 (10.953 to 29.815)	11,291 (6,640 to 16,644)	17.033 (10.149 to 26.497)	–0.56 (–0.65 to –0.48)

East Asia	39,578 (26,460 to 51,543)	4.891 (3.396 to 6.470)	58,735 (42,657 to 85,576)	2.944 (2.163 to 4.257)	–1.58 (–1.91 to –1.25)

Eastern Europe	12,718 (8,942 to 19,035)	4.880 (3.462 to 7.273)	30,340 (24,624 to 38,511)	9.201 (7.592 to 11.465)	2.19 (1.41 to 2.97)

Eastern Sub-Saharan Africa	19,452 (9,300 to 27,713)	18.859 (10.026 to 26.385)	30,577 (20,704 to 42,521)	12.698 (8.307 to 18.379)	–1.27 (–1.33 to –1.21)

High-income Asia Pacific	56,708 (51,503 to 63,521)	30.028 (27.189 to 33.628)	70,200 (56,545 to 87,586)	11.927 (9.769 to 15.133)	–2.95 (–3.16 to –2.74)

High-income North America	93,395 (81,710 to 110,657)	26.030 (22.977 to 30.502)	115,613 (94,261 to 149,324)	17.326 (14.308 to 22.013)	–1.31 (–1.63 to –0.98)

North Africa and Middle East	65,022 (48,864 to 88,288)	31.364 (24.077 to 42.235)	98,611 (78,336 to 139,383)	19.510 (15.721 to 27.224)	–1.52 (–1.66 to –1.39)

Oceania	226 (119 to 438)	4.387 (2.743 to 7.405)	382 (236 to 646)	3.444 (2.294 to 5.510)	–0.80 (–1.07 to –0.54)

South Asia	59,659 (34,658 to 90,228)	9.001 (5.364 to 12.933)	137,673 (95,986 to 174,664)	8.904 (6.231 to 11.330)	–0.02 (–0.21 to 0.17)

Southeast Asia	9,107 (6,313 to 18,147)	3.453 (2.359 to 6.709)	21,562 (15,607 to 36,749)	3.565 (2.573 to 5.998)	0.10 (–0.05 to 0.24)

Southern Latin America	6,737 (6,027 to 7,543)	14.667 (13.100 to 16.438)	8,346 (7,421 to 9,708)	9.604 (8.576 to 11.103)	–1.19 (–1.95 to –0.42)

Southern Sub-Saharan Africa	6,003 (3,771 to 7,057)	14.917 (9.347 to 17.622)	8,579 (6,268 to 10,727)	12.076 (8.712 to 15.013)	–0.80 (–1.15 to –0.45)

Tropical Latin America	27,336 (26,617 to 27,999)	24.213 (23.402 to 24.925)	45,717 (43,132 to 47,817)	17.818 (16.800 to 18.626)	–0.97 (–1.61 to –0.32)

Western Europe	165,595 (152,458 to 182,826)	28.694 (26.488 to 31.463)	165,605 (142,786 to 193,314)	15.992 (14.073 to 18.410)	–1.87 (–2.14 to –1.59)

Western Sub-Saharan Africa	18,725 (10,526 to 26,208)	18.250 (10.508 to 26.299)	31,996 (19,264 to 47,153)	12.936 (7.948 to 19.607)	–1.10 (–1.19 to –1.02)


Abbreviations: AAPC, average annual percentage change; CI, confidence interval; DALYs, disability-adjusted life years; DMVD, degenerative mitral valve disease; SDI, sociodemographic index; UI, uncertainty interval.

### Sociodemographic index regional level

**Figure 1:**
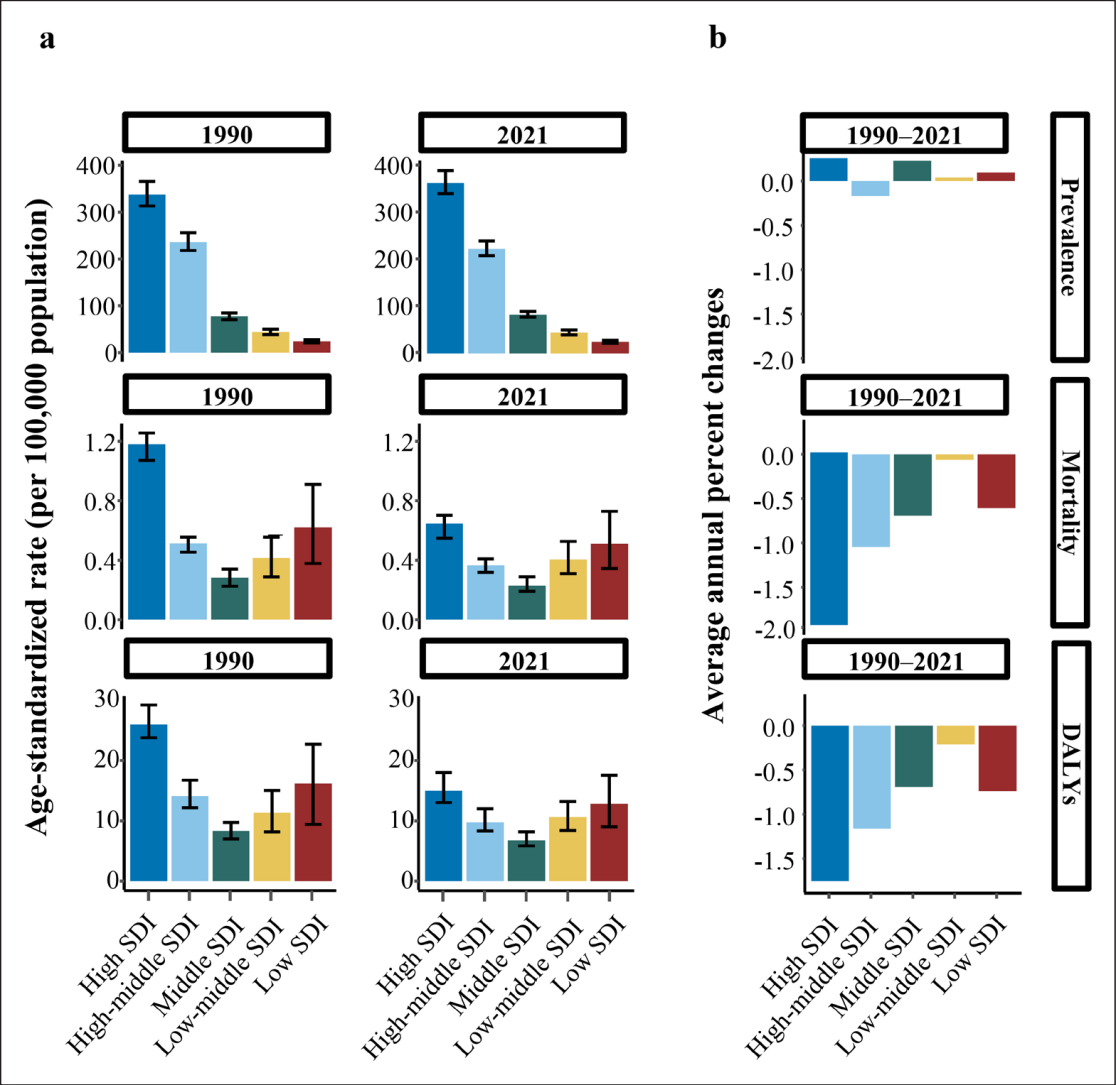
Temporal trend of DMVD burden at SDI levels, 1990–2021. **(a)** Age-standardized prevalence, mortality, and disability-adjusted life year rates (per 100,000 population) between 1990 and 2021. **(b)** Average annual percent changes of prevalence, mortality, and DALYs from 1990 to 2021. Abbreviations: DALYs, disability-adjusted life years; DMVD, degenerative mitral valve disease; SDI, sociodemographic index.

In 2021, high sociodemographic index (SDI) regions exhibited the highest disease burden, with ASPR, ASMR, and ASDR of 364.237 (95% UI: 341.570 to 390.641), 0.646 (95% UI: 0.548 to 0.702), and 15.008 (95% UI: 13.019 to 17.984), respectively (Tables 1, 2, 3 and Figure 1a). High SDI regions accounted for approximately 50% of global DMVD cases and exhibited the most substantial disease burden (Table 1). However, ASMR and ASDR demonstrated a downward trend across all SDI regions from 1990 to 2021, particularly in high SDI regions, with AAPCs of –1.95% (95% CI: –2.19 to –1.70) and –1.75% (95% CI: –1.91 to –1.60), respectively (Tables 2 and 3 and Figure 1b).

### GBD regional level

**Figure 2:**
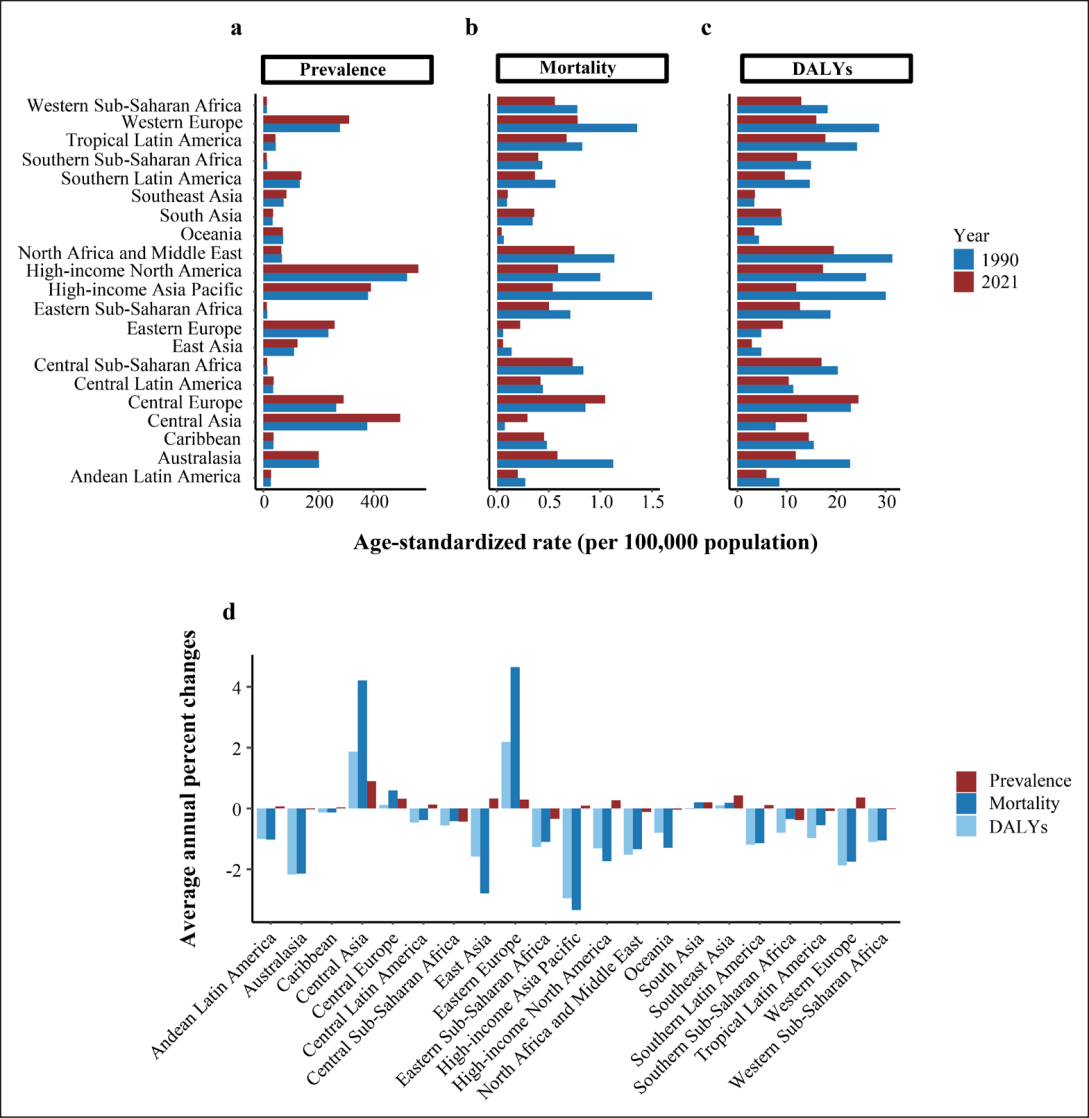
Temporal trend of DMVD burden at region levels, 1990–2021. **(a)** Age-standardized prevalence rate in 2021. **(b)** Age-standardized mortality rate in 2021. **(c)** Age-standardized DALY rates in 2021. **(d)** Average annual percent changes of age-standardized prevalence, mortality, and disability-adjusted life year rates from 1990 to 2021. Abbreviations: DALYs, disability-adjusted life years; DMVD, degenerative mitral valve disease.

In 2021, the highest ASPR for DMVD was observed in high-income North America (562.804 per 100,000 persons; 95% UI: 535.226 to 594.686), Central Asia (496.926 per 100,000 persons; 95% UI: 420.359 to 589.812), and high-income Asia Pacific (390.613 per 100,000 persons; 95% UI: 366.971 to 418.011) (Table 1 and Figure 2a). Over the past 32 years, the ASPR trends varied across 21 regions. The most significant increases occurred in Central Asia, Southeast Asia, Western Europe, and East Asia, with AAPCs of 0.90% (95% UI: 0.85 to 0.94), 0.43% (95% UI: 0.41 to 0.44), 0.36% (95% UI: 0.33 to 0.39), and 0.33% (95% UI: 0.24 to 0.42), respectively. However, Central Sub-Saharan Africa exhibited the most pronounced decline trend, with an AAPC of –0.43% (95% UI: –0.45 to –0.42) (Table 1 and Figure 2d).

In 2021, Central Europe exhibited the highest ASMR at 1.048 per 100,000 persons (95% UI: 0.935 to 1.140) (Table 2 and Figure 2b). From 1990 to 2021, over 70% of regions exhibited a downward trend in ASMR, with the largest decreases observed in high-income Asia Pacific, demonstrating an AAPC of –3.34% (95% CI: –3.64 to –3.03) (Table 2 and Figure 2d). However, Eastern Europe and Central Asia experienced the most significant increases in ASMR, with AAPCs of 4.65% (95% CI: 3.79 to 5.52) and 4.21% (95% CI: 2.58 to 5.87), respectively (Table 2 and Figure 2d).

Globally, the ASDR for DMVD demonstrated an overall downward trend. However, a few regions exhibited an upward trend, with the most significant increases observed in Eastern Europe and Central Asia, with AAPCs of 2.19% (95% CI: 1.41 to 2.97) and 1.87% (95% CI: 1.34 to 2.40), respectively (Table 3 and Figure 2d).

### National level

**Figure 3:**
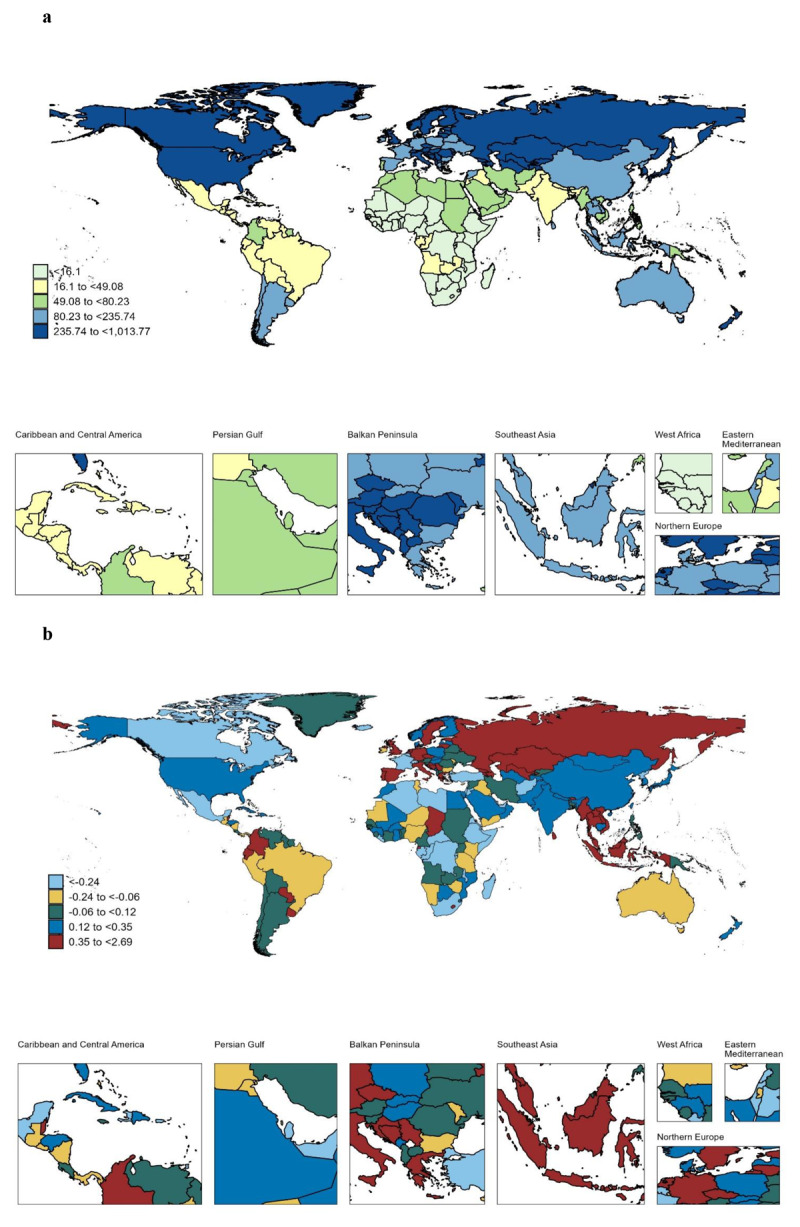
Map showing age-standardized rates and average annual percentage change in global prevalence of DMVD, 1990–2021. **(a)** Age-standardized prevalence rates in 2021. **(b)** Average annual percent changes of age-standardized prevalence rates from 1990 to 2021. Abbreviation: DMVD, degenerative mitral valve disease.

In 2021, the global ASPR for DMVD was 182.127 per 100,000 persons (95% UI: 178.737 to 208.374) (Table 1). The highest ASPR was observed in Georgia (1,013.767 per 100,000 persons; 95% UI: 860.903 to 1,194.241), Norway (883.241 per 100,000 persons; 95% UI: 839.582 to 929.201), Italy (924.860 per 100,000 persons; 95% UI: 882.157 to 966.441), and Greenland (605.583 per 100,000 persons; 95% UI: 488.192 to 732.339) (Figure 3a and Supplementary Table 1). Conversely, the countries with the lowest ASPR were Liberia (11.314 per 100,000 persons; 95% UI: 8.988 to 14.441), Burundi (11.470 per 100,000 persons; 95% UI: 9.019 to 14.159), Ethiopia (11.522 per 100,000 persons; 95% UI: 10.437 to 12.667), and Mali (11.813 per 100,000 persons; 95% UI: 9.237 to 14.932) (Figure 3a and Supplementary Table 1). Over the past 31 years, ASPR changes have varied significantly across countries. Georgia exhibited the most significant increase, with an AAPC of 2.69% (95% UI: 2.57 to 2.81), followed by Greece and Grenada, with AAPCs of 1.56% (95% UI: 1.51 to 1.61) and 1.05% (95% UI: 0.85 to 1.25), respectively (Figure 3b and Supplementary Table 1).

For the ASMR of DMVD, the Netherlands demonstrated the highest ASMR (1.855 per 100,000 persons; 95% UI: 1.554 to 2.065), Hungary (1.599 per 100,000 persons; 95% UI: 1.422 to 1.765), Georgia (1.308 per 100,000 persons; 95% UI: 1.133 to 1.509) (Supplementary Table 2 and Supplementary Figure 1a). From 1990 to 2021, the most significant increases in ASMR were observed in Georgia (AAPC: 10.11%; 95% CI: 7.34 to 12.96), Turkmenistan (AAPC: 5.60%; 95% CI: 2.72 to 8.57), Kazakhstan (AAPC: 5.15%; 95% CI: 2.83 to 7.52), Ukraine (AAPC: 4.85%; 95% CI: 2.35 to 7.40), and the Russian Federation (AAPC: 4.42%; 95% CI: 3.50 to 5.35) (Supplementary Table 2 and Supplementary Figure 1b). Approximately 76% of countries exhibited a downward trend in ASDR. However, Georgia, the Russian Federation, and Poland demonstrated significant increases in ASDR, with AAPCs of 5.62% (95% CI: 4.43 to 6.82), 2.35% (95% CI: 1.60 to 3.10), and 2.13% (95% CI: 1.27 to 3.00), respectively. For more information on DALYs, refer to Supplementary Table 3 and Supplementary Figure 2.

### Sex level

In 2021, the global prevalence of DMVD was 5,665,309 in women (95% UI: 5,281,762 to 6,073,695) and 9,829,338 in men (95% UI: 9,179,556 to 10,615,234), accounting for 36.6% and 63.4% of the total cases, respectively (Table 1). Between 1990 and 2021, the decreases in ASPR for DMVD were more significant in women than in men, with an AAPC of –0.33% compared to –0.18% (Table 1). In 2021, despite the SDI level, the ASPR for men was higher than that for women (Supplementary Table 4 and Supplementary Figure 3). From 1990 to 2021, trends in ASPR varied across different SDI regions and between sexes (Supplementary Table 4 and Supplementary Figure 4).

Compared to 1990, the reduction in ASMR for DMVD was more pronounced in women than in men (Table 2). Across most SDI regions, a downward trend in ASMR was observed for both males and females, except in the low-middle SDI region, where ASMR in men increased, demonstrating an AAPC of 0.18% (95% CI: 0.08 to 0.28) (Supplementary Table 5 and Supplementary Figures 3 and 4). Despite the SDI level, the decline in ASMR for women was more significant than that for men (Supplementary Table 5 and Supplementary Figure 4). From 1990 to 2021, the trend in ASDR for both males and females was similar to that in ASMR, with ASDR for DMVD decreasing in men (from 15.869 to 11.573 per 100,000 persons) and in women (from 17.622 to 11.278 per 100,000 persons) (Table 3). The most significant decreases were observed in the high SDI region, with AAPCs of –1.44% for men and –2.04% for women (Supplementary Table 6 and Supplementary Figures 3 and 4).

### Age level

**Figure 4:**
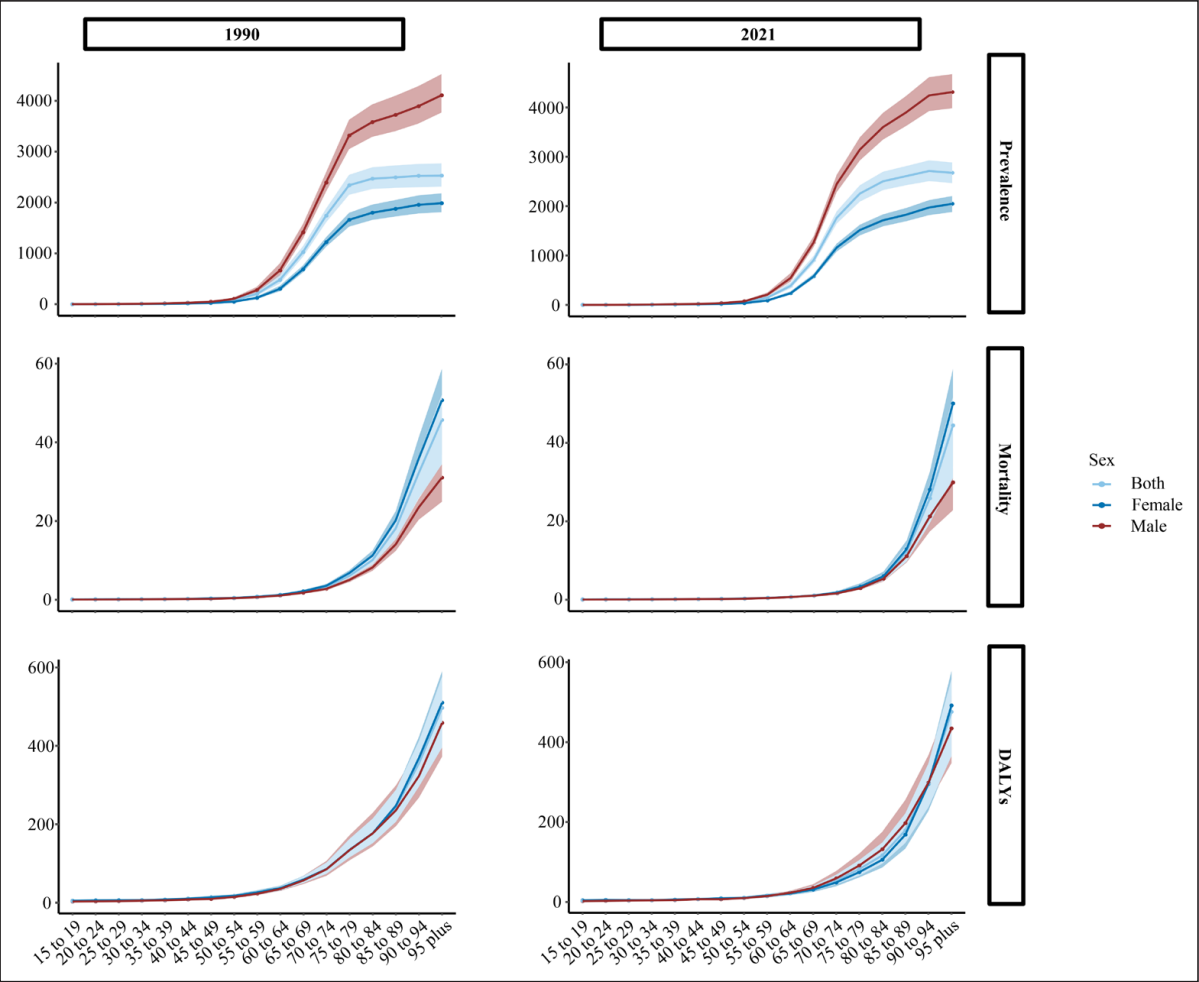
Temporal trend of DMVD burden at sex and age levels, 1990–2021. Abbreviations: DALYs, disability-adjusted life years; DMVD, degenerative mitral valve disease.

Over the past 32 years, global rates of DMVD prevalence, mortality, and DALYs increased with age in both men and women (Figure 4). Notably, across all age groups, men exhibited higher global prevalence rates, while women had higher global mortality rates in 2021 (Figure 4). From 1990 to 2021, trends in global prevalence rates varied across different age groups. The most significant decline was seen in the 65–69 age group, with an RC of –117.252 per 100,000 persons. In contrast, an increasing trend in prevalence was observed in age groups over 80 years, particularly in the 90–94 age group, which showed an RC of 187.409 per 100,000 persons (Supplementary Table 7 and Supplementary Figure 5). Compared to 1990, both mortality and DALY rates showed a downward trend across all age groups (Supplementary Tables 8 and 9 and Supplementary Figure 5). For more details on mortality and DALYs, refer to Supplementary Tables 8 and 9 and Supplementary Figure 5.

Supplementary Figures 6–8 present detailed trends in the prevalence, mortality, and DALYs rates of DMVD by age group across the five SDI regions in 2021. The prevalence of DMVD in 2021 was predominantly concentrated among individuals over 65 years of age (Figure 4). From 1990 to 2021, the RC in prevalence across age groups exhibited varying trends. Notably, an increasing prevalence rate was observed in most older age groups in the high-middle and high SDI regions (Supplementary Figure 6).

In 2021, mortality and DALY rates increased with age across all SDI levels (Supplementary Figures 7 and 8). Compared to 1990, most age groups showed declining trends in both mortality and DALY rates. Across most regions globally, DMVD was predominantly concentrated among patients over 65 years of age.

### Decomposition analysis

**Figure 5:**
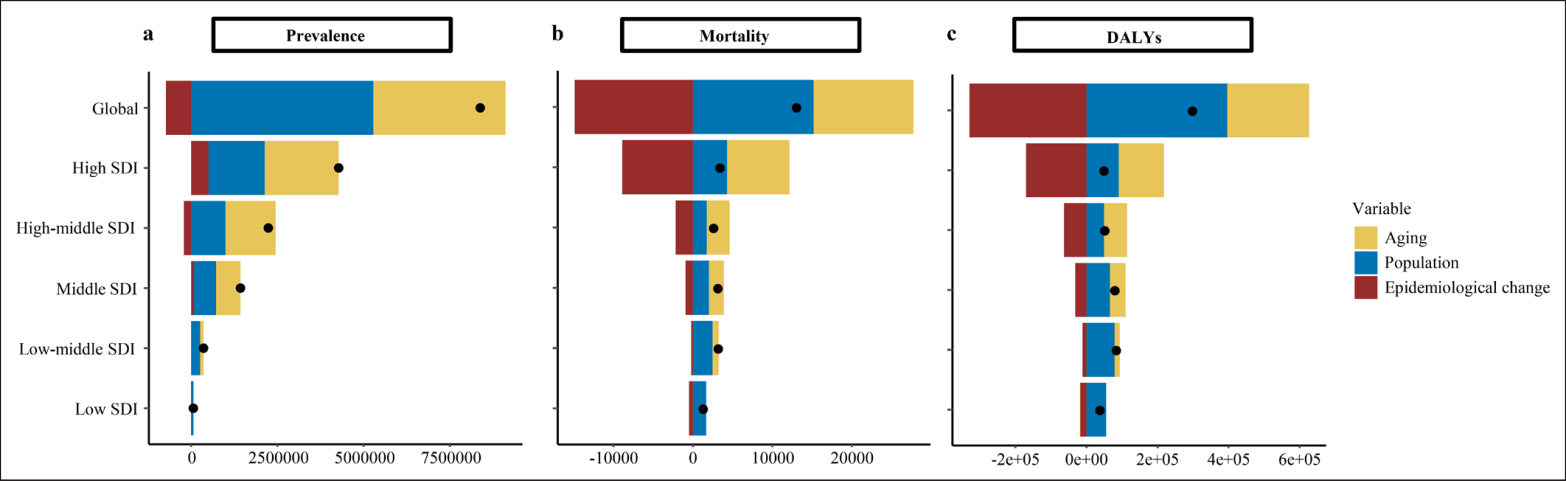
Decomposition analysis of DMVD prevalence, mortality, and DALYs at global and SDI levels, 1990–2021. Abbreviations: DALYs, disability-adjusted life years; DMVD, degenerative mitral valve disease; SDI, sociodemographic index.

Between 1990 and 2021, although the ASPR for DMVD decreased by 8.73%, the global cases of DMVD increased, driven by a 45.72% rise due to population aging and a 63.01% increase due to population growth (Figure 5a and Supplementary Table 10). A significant increase was observed in mortality cases of DMVD globally and across different SDI regions over the past 31 years (Figure 5b and Supplementary Table 10). From 1990 to 2021, population growth and aging contributed 117.91% and 97.52%, respectively, to the increase in DMVD mortality cases, whereas epidemiological changes had opposite impact, decreasing the mortality by 115.43% (Figure 5b and Supplementary Table 10). Over the past 32 years, the mortality cases from DMVD increased across all SDI levels. In high and high-middle SDI regions, this increase was predominantly attributed to aging, whereas in low and low-middle SDI regions, population growth was the primary factor (Figure 5b and Supplementary Table 10). Epidemiological changes consistently alleviated the DMVD mortality burden, particularly in high SDI regions, where the reduction accounted for –273.51% (Figure 5b and Supplementary Table 10). The results of decomposition analysis on DALYs were similar to those for mortality. For more information on DALYs, refer to Supplementary Table 10 and Figure 5c.

### Prediction

**Figure 6:**
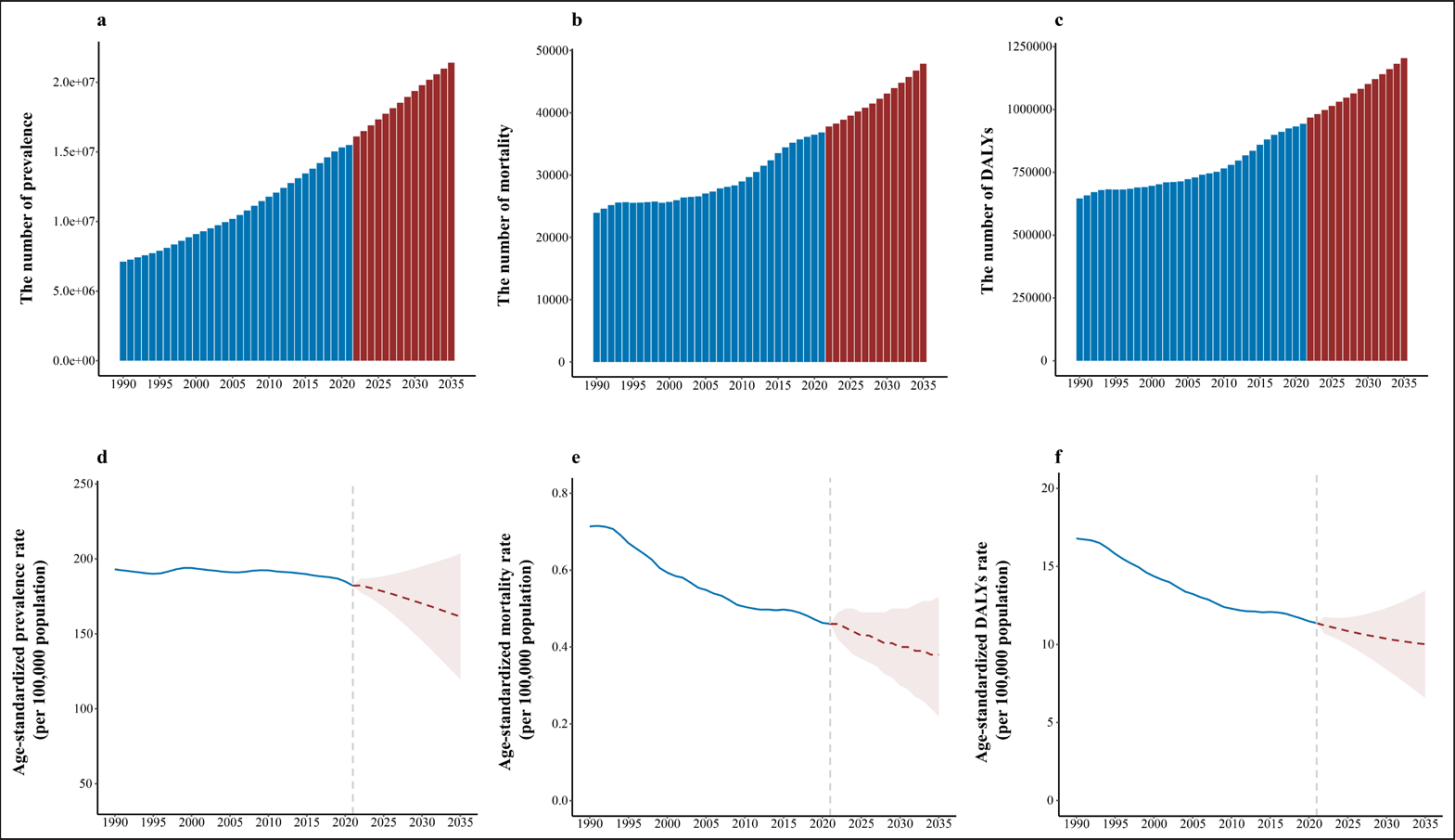
Global trends and forecasts of DMVD, 1990–2035. **(A)** Prevalence cases of DMVD from 1990 to 2035. **(B)** Mortality cases of DMVD from 1990 to 2035. **(C)** DALYs cases of DMVD from 1990 to 2035. **(D)** Age-standardized prevalence rates of DMVD from 1990 to 2035. **(E)** Age-standardized mortality rates of DMVD from 1990 to 2035. **(F)** Age-standardized DALYs rates of DMVD from 1990 to 2035. Abbreviations: DALYs, disability-adjusted life years; DMVD, degenerative mitral valve disease.

By 2035, global projections for DMVD estimate approximately 21.41 million (95% UI: 15,718,776 to 27,102,848) cases of prevalence, 47,878 (95% UI: 28,449 to 67,307) cases of mortality, and 1.20 million (95% UI: 793,487 to 1,615,972) cases of DALYs (Supplementary Table 11 and Figure 6). From 1990 to 2035, the prevalence, mortality, and DALYs of DMVD have shown declining trends (Supplementary Table 11 and Figure 6). In 2035, DMVD is projected to have an ASPR of 161.499 (95% UI: 119.519 to 203.479) per 100,000 persons, an ASMR of 0.379 (95% UI: 0.224 to 0.534) per 100,000 persons, and an ASDR of 10.009 (95% UI: 6.572 to 13.446) per 100,000 persons (Supplementary Table 11 and Figure 6).

## Discussion

This study represents the first exhaustive assessment of the prevalence, mortality, and DALYs of DMVD over the past 31 years using GBD 2021 data on a global scale. Our analysis spanned three decades and covered an extensive geographic range, encompassing six continents, 204 countries, and 20 age groups, thereby providing a comprehensive depiction of DMVD’s burden across various populations and over time. Our results reveal a general decline in the disease burden globally over the past 31 years, consistent across different genders. Nonetheless, there are substantial regional and national disparities. Decomposition analysis reveals that epidemiological and demographic changes are the main drivers behind the improvement in disease burden, although population aging has also exerted a negative impact.

From 1990 to 2021, the global disease burden of DMVD showed overall improvement. Notably, there was a slight decrease in the global prevalence of DMVD, while mortality and DALYs experienced a continuous and marked decline. This divergence may reflect temporal advances in cardiovascular care. In 2014, the American College of Cardiology and the American Heart Association released a guideline for VHD (20). This guideline, which has since been updated multiple times, is widely adopted, and establishes standardized treatment and management protocols (21,22). With new insights into the natural history of VHD, advancements in imaging diagnostics, and improvements in catheter and surgical interventions, the field of VHD has seen rapid development over the past decade. The COAPT trial demonstrated that transcatheter edge-to-edge repair (TEER) reduced 5-year mortality by 28% in patients with severe mitral regurgitation and heart failure (23). Furthermore, US registry data revealed an 88.6% 1-year survival rate among mitral regurgitation patients undergoing TEER intervention (24). These findings suggest that the expanding clinical adoption of transcatheter heart valve intervention and the rapid progression of catheter-based interventions may contribute to improved prognosis in patients with DMVD.

At the SDI level, the highest ASPR, ASMR, and ASDR were observed in high SDI regions. This finding emphasizes the influence of socioeconomic disparities in the global burden of DMVD. The highest burden in high SDI regions may be due to the impact of an aging population. In addition, the widespread availability of echocardiography in high SDI regions has increased the detection rates of DMVD. Strategic advances, such as the broad implementation of early screening and diagnostic practices, have also contributed to the increased prevalence. Mortality and DALYs declined across various SDI regions, especially in high SDI regions. This decline correlates strongly with the popularity of surgical procedures and transcatheter valve interventions, which are predominantly undertaken in high SDI regions.

In 2021, the burden of DMVD exhibits significant global heterogeneity. In Central Asia, the higher ASPR and the rapidly increasing ASMR can be partly attributed to socioeconomic development and healthcare factors. This underscores substantial regional challenges in healthcare access and quality, exacerbated by advancements in ultrasound technology that improve DMVD detection but are mismatched by limited treatment resources. In contrast, trends within Asia vary notably, with an increase in ASPR in Central Asia and a decline in the high-income Asia-Pacific region, highlighting the imbalance in socioeconomic development and healthcare policies. Meanwhile, the DMVD burden appears lower in most African regions, although this finding may be linked to underdiagnosis and diagnostic confusion with rheumatic heart disease in these areas.

This study also analyzed the gender differences in the disease burden of DMVD. We observed a lower DMVD prevalence and higher mortality and DALYs in women compared to men. Such gender differences are not only attributable to physiological differences in anatomical and metabolic processes between sexes but may also be indirectly influenced by social, cultural, and healthcare accessibility factors. In some regions, due to social, cultural, and economic constraints, women often receive less medical care than men, potentially resulting in a higher prevalence of earlier diagnosis and treatment of DMVD among men. Conversely, women frequently have high mortality rates due to delayed diagnosis, detection at advanced stages of the disease, and inadequate systematic treatment. Studies indicate that males undergo a higher percentage of DMVD surgeries than females (25). A series of research on DMVD surgeries reported that women present with more symptoms at referral, exhibit a higher likelihood of heart failure, experience lower rates of successful repair (26,27), and have higher surgical mortality and lower postoperative survival rates compared to men (28,29). In addition, women are prone to pathological changes, including mitral leaflet fibrosis and annulus calcification (30), which are recognized risk factors for increased mortality. These factors partially explain the higher mortality rate and DALYs in women than in men; however, the specific mechanisms underlying this gender difference require further investigation. It is therefore imperative to develop additional gender-specific treatment strategies to enhance therapeutic outcomes and tailor treatment plans more effectively.

Based on age patterns, DMVD prevalence, mortality, and DALYs are primarily concentrated among the elderly population aged over 65 years of age. Globally, the prevalence, mortality, and DALYs demonstrated an increasing trend with advancing age, irrespective of gender. A global decline in mortality and DALYs of DMVD over the past 31 years has been particularly pronounced in the 80–95 years age group, especially within middle SDI regions. In the context of an aging population, the provision of nursing care and medical resources for elderly DMVD patients must be enhanced.

In addition, we performed a decomposition analysis of DMVD to determine the primary factors influencing changes in the burden of DMVD. Although the epidemiology of DMVD has declined, the prevalence, mortality, and DALYs associated with DMVD have increased globally due to aging and population growth. The primary determinants of the disease burden associated with DMVD vary significantly across regions with differing economic statuses. The predominant factor driving the high SDI region is aging, whereas population growth is the principal contributor in the low SDI region. Forecast analysis indicates that with the progression of global population aging, the number of people with DMVD is projected to increase significantly. These patients frequently present with multiple cardiovascular comorbidities, posing challenges to healthcare systems and resulting in increased mortality, DALYs, and healthcare costs.

Achieving a one-third reduction in premature mortality from non-communicable diseases between 2015 and 2030 through prevention and treatment is a sustainable development goal of the World Health Organization (31,32). DMVD is a significant type of non-communicable disease; early diagnosis and treatment are crucial for this goal. Currently, comprehensive analyses of mortality and DALYs associated with DMVD are lacking across different countries and regions worldwide. It is crucial to promptly describe and update the data on the global burden of DMVD. As part of the GBD 2021 study, this research represents the first utilization of GBD 2021 data to systematically evaluate the global disease burden of DMVD from 1990 to 2021. Notably, this study introduces an innovative assessment of changes in the DMVD disease burden using a decomposition analysis method, quantitatively analyzing the driving factors behind these changes. This information will enable policymakers to grasp the extent of the issue and develop effective prevention and control strategies for DMVD.

This study has several limitations. First, the GBD database primarily relies on national and regional reported data and publications. For many countries with limited raw data, the GBD 2021 working group addressed the issue of data scarcity through hierarchical Bayesian models. This model can use global data or data from neighboring countries to fill in gaps for countries with insufficient data. However, this does not guarantee that the data for these countries are accurate, as evidenced by the significant expansion of the 95% UI. These results should be interpreted with caution. Second, variations exist in the quality of vital registration systems and their disease management across countries and regions, potentially affecting data completeness, introducing bias, and impacting the accuracy of the results. Third, this study was unable to assess the comorbidities or elucidate the underlying etiologies and severity of DMVD. Consequently, high-quality, real-world studies are required to validate the findings of this study.

## Conclusion

This study conducted a comprehensive assessment of the global, regional, and national disease burden of DMVD using data from GBD 2021 and analyzed the trends between 1990 and 2021. The findings indicate that the disease burden due to DMVD has generally declined globally over the past 31 years, independent of gender. However, significant regional and national variations exist. Notably, the disease burden significantly decreased in high SDI regions while exhibiting an opposite trend of increase in some low-income countries and regions. Enhancing the global access to diagnostic imaging, surgical, and transcatheter interventions is essential to alleviate the disease burden of DMVD.

## Additional Files

The additional files for this article can be found as follows:

10.5334/gh.1489.s1 Supplementary Material. Supplementary Tables 1 to 11 and Supplementary Figures 1 to 8.

## Abbreviations

AAPC, average annual percent change

ASDRs, age-standardized disability-adjusted life year rates

ASMRs, age-standardized mortality rates

ASPRs, age-standardized prevalence rates

CI, confidence interval

DALYs, disability-adjusted life years

DMVD, degenerative mitral valve disease

DWs, disability weights

GBD, Global Burden of Disease

RC, rate change

SDI, sociodemographic index

TEER, transcatheter edge-to-edge repair

UI, uncertainty interval

VHD, valvular heart disease

YLDs, years lived with disability

YLLs, years of life lost

## References

[1] Coffey S, Roberts-Thomson R, Brown A, et al. Global epidemiology of valvular heart disease. Nature Reviews Cardiology. 2021;18(12):853–864. DOI: 10.1038/s41569-021-00570-z 34172950

[2] Roth GA, Mensah GA, Johnson CO, et al. Global burden of cardiovascular diseases and risk factors, 1990–2019: Update from the GBD 2019 study. Journal of the American College of Cardiology. 2020;76(25):2982–3021. DOI: 10.1016/j.jacc.2020.11.010 33309175 PMC7755038

[3] Vaduganathan M, Mensah GA, Turco JV, et al. The global burden of cardiovascular diseases and risk: A compass for future health. Journal of the American College of Cardiology. 2022;80(25):2361–2371. DOI: 10.1016/j.jacc.2022.11.005 36368511

[4] Anyanwu AC, Adams DH. Etiologic classification of degenerative mitral valve disease: Barlow’s disease and fibroelastic deficiency. Seminars in Thoracic and Cardiovascular Surgery. 2007;19(2):90–96. DOI: 10.1053/j.semtcvs.2007.04.002 17870001

[5] Basso C, Perazzolo Marra M. Mitral annulus disjunction: Emerging role of myocardial mechanical stretch in arrhythmogenesis. Journal of the American College of Cardiology. 2018;72(14):1610–1612. DOI: 10.1016/j.jacc.2018.07.069 30261962

[6] Sabbag A, Essayagh B, Barrera JDR, et al. EHRA expert consensus statement on arrhythmic mitral valve prolapse and mitral annular disjunction complex in collaboration with the ESC Council on valvular heart disease and the European Association of Cardiovascular Imaging endorsed by the Heart Rhythm Society, by the Asia Pacific Heart Rhythm Society, and by the Latin American Heart Rhythm Society. Europace. 2022;24(12):1981–2003. DOI: 10.1093/europace/euac125 35951656 PMC11636573

[7] van Wijngaarden AL, Kruithof BPT, Vinella T, et al. Characterization of degenerative mitral valve disease: Differences between fibroelastic deficiency and Barlow’s disease. Journal of Cardiovascular Development and Disease. 2021;8(2):23. DOI: 10.3390/jcdd8020023 PMC7926852 33671724

[8] Abramowitz Y, Jilaihawi H, Chakravarty T, et al. Mitral annulus calcification. Journal of the American College of Cardiology. 2015;66(17):1934–1941. DOI: 10.1016/j.jacc.2015.08.872 26493666

[9] Fornes P, Heudes D, Fuzellier JF, et al. Correlation between clinical and histologic patterns of degenerative mitral valve insufficiency: A histomorphometric study of 130 excised segments. Cardiovascular Pathology. 1999;8(2):81–92. DOI: 10.1016/S1054-8807(98)00021-0 10724505

[10] Iung B, Vahanian A. Epidemiology of valvular heart disease in the adult. Nature Reviews Cardiology. 2011;8(3):162–172. DOI: 10.1038/nrcardio.2010.202 21263455

[11] Iung B, Baron G, Butchart EG, et al. A prospective survey of patients with valvular heart disease in Europe: The Euro Heart Survey on Valvular Heart Disease. European Heart Journal. 2003;24(13):1231–1243. DOI: 10.1016/S0195-668X(03)00201-X 12831818

[12] GBD 2021 Diseases and Injuries Collaborators. Global incidence, prevalence, years lived with disability (YLDs), disability-adjusted life-years (DALYs), and healthy life expectancy (HALE) for 371 diseases and injuries in 204 countries and territories and 811 subnationallocations, 1990–2021: A systematic analysis for the Global Burden of Disease Study 2021. Lancet (London, England). 2024;403(10440):2133–2161. DOI: 10.1016/S0140-6736(24)00757-8 38642570 PMC11122111

[13] GBD 2021 Causes of Death Collaborators. Global burden of 288 causes of death and life expectancy decomposition in 204 countries and territories and 811 subnational locations, 1990–2021: A systematic analysis for the Global Burden of Disease Study 2021. Lancet (London, England). 2024;403(10440):2100–2132. DOI: 10.1016/S0140-6736(24)00367-2 38582094 PMC11126520

[14] GBD 2021 Risk Factors Collaborators. Global burden and strength of evidence for 88 risk factors in 204 countries and 811 subnational locations, 1990–2021: A systematic analysis for the Global Burden of Disease Study 2021. Lancet (London, England). 2024;403(10440):2162–2203. DOI: 10.1016/S0140-6736(24)00933-4 38762324 PMC11120204

[15] Kim HJ, Fay MP, Feuer EJ, et al. Permutation tests for joinpoint regression with applications to cancer rates. Statistics in Medicine. 2000;19(3):335–351. DOI: 10.1002/(SICI)1097-0258(20000215)19:3<335::AID-SIM336>3.3.CO;2-Q 10649300

[16] Das Gupta P. Standardization and decomposition of rates from cross-classified data. Genus. 1994;50(3–4):171–196. 12319256

[17] Holford TR. The estimation of age, period and cohort effects for vital rates. Biometrics. 1983;39(2):311–324. DOI: 10.2307/2531004 6626659

[18] Møller B, Fekjaer H, Hakulinen T, et al. Prediction of cancer incidence in the Nordic countries: Empirical comparison of different approaches. Statistics in Medicine. 2003;22(17):2751–2766. DOI: 10.1002/sim.1481 12939784

[19] Jürgens V, Ess S, Cerny T, et al. A Bayesian generalized age-period-cohort power model for cancer projections. Statistics in Medicine. 2014;33(26):4627–4636. DOI: 10.1002/sim.6248 24996118

[20] Nishimura RA, Otto CM, Bonow RO, et al. 2014 AHA/ACC guideline for the management of patients with valvular heart disease: Executive summary: A report of the American College of Cardiology/American Heart Association Task Force on Practice Guidelines. Journal of the American College of Cardiology. 2014;63(22):2438–2488. DOI: 10.1016/j.jacc.2014.02.537 24603192

[21] Nishimura RA, Otto CM, Bonow RO, et al. 2017 AHA/ACC focused update of the 2014 AHA/ACC guideline for the management of patients with valvular heart disease: A report of the American College of Cardiology/American Heart Association Task Force on Clinical Practice Guidelines. Journal of the American College of Cardiology. 2017;70(2):252–289. DOI: 10.1016/j.jacc.2017.03.011 28315732

[22] Otto CM, Nishimura RA, Bonow RO, et al. 2020 ACC/AHA guideline for the management of patients with valvular heart disease: Executive summary: A report of the American College of Cardiology/American Heart Association Joint Committee on Clinical Practice Guidelines. Journal of the American College of Cardiology. 2021;77(4):450–500. DOI: 10.1016/j.jacc.2020.11.035 33342587

[23] Stone GW, Abraham WT, Lindenfeld J, et al. Five-year follow-up after transcatheter repair of secondary mitral regurgitation. The New England Journal of Medicine. 2023;388(22):2037–2048. DOI: 10.1056/NEJMoa2300213 36876756

[24] Makkar RR, Chikwe J, Chakravarty T, et al. Transcatheter mitral valve repair for degenerative mitral regurgitation. JAMA. 2023;329(20):1778–1788. DOI: 10.1001/jama.2023.7089 37219553 PMC10208157

[25] Suri RM, Schaff HV, Dearani JA, et al. Survival advantage and improved durability of mitral repair for leaflet prolapse subsets in the current era. The Annals of Thoracic Surgery. 2006;82(3):819–826. DOI: 10.1016/j.athoracsur.2006.03.091 16928491

[26] Mantovani F, Clavel MA, Michelena HI, et al. Comprehensive imaging in women with organic mitral regurgitation: Implications for clinical outcome. JACC Cardiovascular Imaging. 2016;9(4):388–396. DOI: 10.1016/j.jcmg.2016.02.017 27056158

[27] Vakamudi S, Jellis C, Mick S, et al. Sex differences in the etiology of surgical mitral valve disease. Circulation. 2018;138(16):1749–1751. DOI: 10.1161/CIRCULATIONAHA.118.035789 30354470

[28] Kislitsina ON, Zareba KM, Bonow RO, et al. Is mitral valve disease treated differently in men and women? European Journal of Preventive Cardiology. 2019;26(13):1433–1443. DOI: 10.1177/2047487319833307 30832507

[29] Vassileva CM, McNeely C, Mishkel G, et al. Gender differences in long-term survival of Medicare beneficiaries undergoing mitral valve operations. The Annals of Thoracic Surgery. 2013;96(4):1367–1373. DOI: 10.1016/j.athoracsur.2013.04.055 23915585

[30] Elmariah S, Budoff MJ, Delaney JA, et al. Risk factors associated with the incidence and progression of mitral annulus calcification: The multi-ethnic study of atherosclerosis. American Heart Journal. 2013;166(5):904–912. DOI: 10.1016/j.ahj.2013.08.015 24176447 PMC3978772

[31] NCD Countdown 2030 Collaborators. NCD countdown 2030: Worldwide trends in non-communicable disease mortality and progress towards Sustainable Development Goal target 3.4. Lancet (London, England). 2018;392(10152):1072–1088. DOI: 10.1016/S0140-6736(18)31992-5 30264707

[32] NCD Countdown 2030 Collaborators. NCD Countdown 2030: Efficient pathways and strategic investments to accelerate progress towards the Sustainable Development Goal target 3.4 in low-income and middle-income countries. Lancet (London, England). 2022;399(10331):1266–1278. DOI: 10.1016/S0140-6736(21)02347-3 35339227 PMC8947779

