# Supplementary material for: The Burden and Trends of Degenerative Mitral Valve Disease at the Global, Regional, and National Levels From 1990 to 2021, With Projections to 2035
Source: Glob Heart. 2025 Oct 28;20(1):101. doi: 10.5334/gh.1489 (PMC12577541; doi:10.5334/gh.1489)
Supplement: Supplementary Material. — Supplementary Tables 1 to 11 and Supplementary Figures 1 to 8. [file gh-20-1-1489-s1.pdf]

Supplementary Material

The burden and trends of degenerative mitral valve disease at the global, regional, and national levels from 1990 to 2021, with projections to 2035

Table of contents

Supplementary Methods 4

1 Global burden of disease (GBD) overview 4

2 Non-fatal outcome estimation 4

3 Data sources 4

4 Data processing 5

5 The definition of degenerative mitral valve disease (DMVD) in GBD 2021 5

6 SDI 6

7 Joinpoint regression analysis 6

8 Decomposition analysis section 7

Reference 8

Supplementary Table 1: Age-standardized prevalence rates and average annual percentage change for DMVD at national level, 1990-2021 9

Supplementary Table 2: Age-standardized mortality rates and average annual percentage change for DMVD at national level, 1990-2021 25

Supplementary Table 3: Age-standardized DALYs rates and average annual percentage change for DMVD at national level, 1990-2021 41

Supplementary Table 4: Age-standardized prevalence rates and average annual percentage change for DMVD at sex and SDI levels, 1990-2021 57

Supplementary Table 5: Age-standardized mortality rates and average annual percentage change for DMVD at sex and SDI levels, 1990-2021 58

Supplementary Table 6: Age-standardized DALYs rates and average annual percentage change for DMVD at sex and SDI levels, 1990-2021 59

Supplementary Table 7: The prevalence rates and rate change for DMVD at sex and age levels, 1990-2021 60

Supplementary Table 8: The mortality rates and rate change for DMVD at sex and age levels, 1990-2021 63

Supplementary Table 9: The DALYs rates and rate change for DMVD at sex and age levels, 1990-2021 66

Supplementary Table 10: Decomposition analysis of DMVD prevalence, mortality and DALYs at global and SDI levels, 1990-2021 69

Supplementary Table 11: Predicted prevalence, mortality and DALYs of DMVD from 2021 to 2035 reported by 5-year intervals at global level 71

Supplementary Figure 1: Map showing age-standardized rates and average annual percentage change in global mortality of DMVD, 1990-2021 72

Supplementary Figure 2: Map showing age-standardized rates and average annual percentage change in global DALYs of DMVD, 1990-2021 74

Supplementary Figure 3: Age-standardized prevalence, mortality and DALYs rate of DMVD at SDI and sex levels, 1990-2021 76

Supplementary Figure 4: Average annual percent changes of age-standardized rates of DMVD at SDI and sex levels, 1990-2021 77

Supplementary Figure 5: Rate change in prevalence, mortality and DALYs of DMVD at sex and age levels, 1990-2021 78

Supplementary Figure 6: Rate and rate change in prevalence of DMVD at SDI and age levels, 1990-2021 79

Supplementary Figure 7: Rate and rate change in mortality of DMVD at SDI and age levels, 1990-2021 80

Supplementary Figure 8: Rate and rate change in DALYs of DMVD at SDI and age levels, 1990-2021 81

Supplementary Methods

1 Global burden of disease (GBD) overview

GBD 2021 provides an in-depth assessment of 369 diseases and 88 risk factors using the latest data and standardized data processing methods. This study covers the number and rate of incidence, prevalence, mortality and disability-adjusted life years (DALYs) during 1980-2021[1-3]. By dividing study population according to region, gender, age group, and sociodemographic index (SDI), GBD 2021 demonstrates the epidemiological characteristics of various diseases[1]. All data in this study were available in https://vizhub.healthdata.org/gbd-results.

2 Non-fatal outcome estimation

In GBD 2021, for non-fatal estimates, data sources included scientific literature, household survey data, epidemiological surveillance data, disease registry data, clinical informatics data, and other sources[1]. The GBD 2021 working group has published a process for calculating non-fatal indicators of disease and injury: incidence, prevalence and DALYs: (1) Gathering data sources through identification and extraction; (2) Adjusting data; (3) Estimating prevalence and incidence by cause and sequelae using DisMod-MR 2.1 or other modeling strategies for selected cause groups; (4) Impairment estimates; (5) Analyzing severity distributions; (6) Incorporating the weight of persons with disabilities; (7) Adjusting comorbidity; (8) The estimation of years lived with disability (YLDs) by sequelae and causes.

3 Data sources

Data for GBD 2021 mainly come from censuses, household surveys, civil registration and vital statistics, disease registries, health service utilization, air pollution monitors, satellite imagery, disease notifications,

and other sources, with only a small portion coming from the published literature. All data for this study were obtained from GBD 2021 and include: (1) Global age- and sex-specific prevalence, mortality and DALYs numbers and age-standardized rate (per 100,000 population) from 1990 to 2021; (2) Regional age- and sex- specific prevalence, mortality, DALYs numbers and age-standardized rate from 1990 to 2021 by SDI categories; (3) National age- and sex- specific incidence, mortality, DALYs numbers and age-standardized rate from 1990 to 2021.

4 Data processing

The data of GBD 2021 is derived from multiple data sources. These data sources are heterogeneous in terms of size, comprehensiveness and level of detail. In order to make multiple data sources comparable across locations, ages, genders, years, and reasons, GBD 2021 takes the following approach: (1) Mapping International Classification of Diseases system to GBD-defined diagnosis groups to simplify disease classification and reduce complexity. (2) Unique identifiers are created for individuals to facilitate association. (3) Collect data for age and sex from 4,722 location-years in 49 countries. (4) Administrative data, reports, and survey data were used to estimate per capita hospitalization rates for each site and population group in the GBD hierarchy to assess whether the data sources captured a complete and representative GBD population. (5) Methods such as crosswalking are used to adjust data bias.

5 The definition of degenerative mitral valve disease (DMVD) in GBD 2021

In GBD 2021, DMVD was defined as a condition where the mitral valve, which separates the two left chambers of the heart, becomes damaged due to weakening of the valve tissue, leading to leakage of blood across the valve. DMVD was defined as physician diagnosis based on echocardiographic findings

of haemodynamically moderate or severe mitral regurgitation according to criteria from the American Heart Association and American College of Cardiology. DMVD did not include disease with an aetiology that was congenital, rheumatic, infectious, traumatic, carcinoid, or functional.

6 SDI

The SDI is a composite indicator of the contextual, social and economic conditions which influence health outcomes. It is the geometric mean of the 0 to 1 indices of the total fertility rate for persons under 25 years of age, the average educational attainment of persons 15 years of age and over, and the lagged distributional income per capita. Based on SDI value, the world's 204 countries or regions were divided into five levels through SDI: low, low-middle, middle, high-middle, and high.

7 Joinpoint regression analysis

Time trend analysis is an important method in epidemiological research. To accurately characterize the epidemiological trends of the disease, Kim et al. first proposed the Joinpoint regression model. The idea of the model is to divide the time change trend of the disease into different intervals by several joinpoints, and to fit and optimize the trend of each interval. The model has been widely used in the field of trend study of disease prevalence and mortality[4].

The population-based epidemiological data were first log-transformed to meet the modeling requirements of the Joinpoint regression model. The grid search method is used to divide the study data into a grid. The grid search method was utilized to build all possible segmentation functions, calculate the sum of squared errors and mean square errors for each possible scenario, and select the grid point with the smallest mean square errors as the coupling point. Monte Carlo permutation tests were used to

determine the optimal number of joinpoints for the Joinpoint regression model. The maximum number of potential joinpoints was set to be 5 and the minimum number of potential joinpoints to be 0. The permutation test starts with the number of joinpoints k = 0 with k_max = 5. If k ≠ k_max, then set k = k+1 and continue the test until the model corresponding to k = k_max is selected as the optimal model.

Annual percent change and average annual percent change (AAPC) along with their 95% confidence intervals are the primary outcome indicators of the Joinpoint model. In this study, AAPC was used to represent the relative changes in the age-standardized rates of prevalence, mortality, and DALYs over the past 30 years, from 1990 to 2021. The parameter calculation method of AAPC involves weighted calculation of the regression coefficients of each interval based on the width w of the segment intervals. Its formula is as follows:

$$AAPC = \left(e^{\frac{\sum w_i \beta_i}{\sum w_i}} - 1 \right) \times 100$$

In this formula, w_i represents each segment function interval, β_i denotes the regression coefficient corresponding to each interval, $\sigma^2 i$ is the variance of β_i, and Z_α represents the corresponding value of the α percentile in the normal distribution.

8 Decomposition analysis section

Decomposition analysis is used to decompose the overall differences of prevalence, mortality and DALYs from 1990 to 2021 into three components, including aging, population growth, and epidemiological changes. Decomposition analyses draw from methods developed by Das Gupta[5]. Both the total burden and the attributable burden are determined, following the methods of Das Gupta, as a product of four factors such that:

$$T_{asgt} = (A_{agt} B_{asgt} C_{asgt})$$

T_{asgt} represents either the total burden or the attributable burden at year. A_{sgt} represents the size of

the population of all ages in a, given gender s, region g (in this article, both are global) and year t. B_{asgt} represents the size of the population of age group a, in a given gender s, region g and year t. C_{asgt} represents the epidemiological rate of age group a, in a given gender s, region g and year t. The contribution of each factor to the total change in total or attributable burden is determined by holding other factors constant and comparing the change in this factor from 1990 to 2021. The calculation process is as follows:

$$E_A = (A_{21} - A_{90})(\frac{B_{90}C_{90} + B_{21}C_{21}}{4} + \frac{B_{90}C_{21} + B_{21}C_{90}}{4})$$

In this equation, EA denotes the proportion of the change in the overall differences that is caused by factor A. The subscript for each factor in the equation represents the year corresponding to each estimate. Considering that the effects are influenced by the order in which the factors are entered, we calculated the average of all combinations of the three factors.

Reference

1. Global incidence, prevalence, years lived with disability (YLDs), disability-adjusted life-years (DALYs), and healthy life expectancy (HALE) for 371 diseases and injuries in 204 countries and territories and 811 subnational locations, 1990-2021: a systematic analysis for the Global Burden of Disease Study 2021. *Lancet (London, England)* 2024;403(10440):2133-61. doi: 10.1016/s0140-6736(24)00757-8 [published Online First: 2024/04/21]
2. Global burden of 288 causes of death and life expectancy decomposition in 204 countries and territories and 811 subnational locations, 1990-2021: a systematic analysis for the Global Burden of Disease Study 2021. *Lancet (London, England)* 2024;403(10440):2100-32. doi: 10.1016/s0140-6736(24)00367-2 [published Online First: 2024/04/07]
3. Global burden and strength of evidence for 88 risk factors in 204 countries and 811 subnational locations, 1990-2021: a systematic analysis for the Global Burden of Disease Study 2021. *Lancet (London, England)* 2024;403(10440):2162-203. doi: 10.1016/s0140-6736(24)00933-4 [published Online First: 2024/05/19]
4. Kim HJ, Fay MP, Feuer EJ, et al. Permutation tests for joinpoint regression with applications to cancer rates. *Statistics in medicine* 2000;19(3):335-51. doi: 10.1002/(sici)1097-0258(20000215)19:3<335::aid-sim336>3.0.co;2-z [published Online First: 2000/01/29]
5. Das Gupta P. Standardization and decomposition of rates from cross-classified data. *Genus* 1994;50(3-4):171-96. [published Online First: 1994/07/01]

Supplementary Table 1: Age-standardized prevalence rates and average annual percentage change for DMVD at national level, 1990-2021

	1990		2021		1990 – 2021
**Nation**	**Cases (95% UI)**	**Age-standardized prevalence per 100 000 population (95% UI)**	**Cases (95% UI)**	**Age- standardized prevalence per 100 000 population (95% UI)**	**AAPC % (95% CI)**
Afghanistan	3601 (2869 to 4434)	57.323 (46.469 to 70.296)	4187 (3463 to 5028)	53.021 (43.500 to 64.504)	-0.26 (-0.3 to -0.21)
Albania	5562 (4572 to 6899)	288.281 (238.036 to 356.252)	13382 (10994 to 16470)	292.987 (242.087 to 358.271)	0.06 (0.04 to 0.07)
Algeria	8488 (6916 to 10172)	73.353 (60.080 to 88.160)	21772 (18027 to 26556)	65.503 (54.472 to 80.253)	-0.37 (-0.41 to -0.33)
American Samoa	18 (14 to 23)	99.441 (77.946 to 124.951)	40 (32 to 49)	93.854 (76.257 to 115.124)	-0.19 (-0.23 to -0.15)
Andorra	87 (71 to 106)	151.014 (123.685 to 184.486)	212 (175 to 252)	137.219 (113.106 to 163.333)	-0.32 (-0.4 to -0.23)
Angola	616 (485 to 769)	16.741 (13.293 to 20.594)	1868 (1477 to 2325)	16.489 (13.036 to 20.287)	-0.04 (-0.06 to -0.01)
Antigua and Barbuda	26 (21 to 31)	46.872 (37.906 to 57.203)	53 (43 to 63)	50.014 (41.227 to 59.262)	0.21 (0.18 to 0.23)
Argentina	41132 (33113 to 50977)	127.007 (102.537 to 157.341)	73854 (59770 to 92359)	128.388 (103.520 to 160.657)	0.02 (-0.03 to 0.07)
Armenia	7833 (6242 to 9746)	314.972 (251.873 to 391.187)	16508 (13687 to 19842)	375.996 (312.084 to 447.886)	0.56 (0.40 to 0.71)
Australia	38260 (31909 to 46594)	187.160 (156.068 to 227.316)	87805 (71336 to 110848)	181.324 (147.623 to 227.566)	-0.11 (-0.20 to -0.03)
Austria	59338 (49917 to 75248)	468.351 (392.81 to 595.988)	90687 (72982 to 135157)	469.772 (381.419 to 707.516)	-0.02

					(-0.14 to 0.10)
Azerbaijan	16898 (13426 to 21174)	371.398 (294.302 to 460.961)	38532 (30932 to 47946)	428.834 (351.466 to 520.930)	0.44<br>(0.33 to 0.56)
Bahamas	57 (45 to 69)	37.214 (29.625 to 45.730)	145 (117 to 181)	36.384 (29.628 to 45.191)	-0.06<br>(-0.11 to -0.01)
Bahrain	97 (78 to 120)	63.734 (51.245 to 79.534)	487 (386 to 609)	61.125 (49.202 to 75.919)	-0.14<br>(-0.18 to -0.10)
Bangladesh	14499 (11249 to 17892)	34.080 (26.619 to 41.965)	46028 (36776 to 57347)	34.699 (27.905 to 43.369)	0.06<br>(0.03 to 0.10)
Barbados	99 (77 to 123)	33.209 (25.747 to 41.122)	164 (131 to 203)	31.781 (25.347 to 39.239)	-0.13<br>(-0.18 to -0.07)
Belarus	29219 (22905 to 36724)	227.060 (179.532 to 282.047)	37747 (29261 to 47406)	230.271 (178.921 to 286.814)	0.05<br>(0.03 to 0.06)
Belgium	18274 (14635 to 22338)	112.004 (89.550 to 136.119)	24735 (20588 to 30270)	100.213 (83.615 to 121.256)	-0.37<br>(-0.39 to -0.34)
Belize	35 (28 to 43)	37.385 (29.983 to 46.413)	126 (104 to 150)	43.395 (36.011 to 51.785)	0.49<br>(0.35 to 0.63)
Benin	265 (211 to 334)	12.831 (10.192 to 16.302)	728 (577 to 898)	12.868 (10.304 to 15.950)	0.01<br>(-0.06 to 0.07)
Bermuda	34 (27 to 41)	53.485 (43.081 to 65.355)	60 (48 to 73)	44.451 (36.235 to 54.027)	-0.58<br>(-0.64 to -0.52)
Bhutan	68 (53 to 85)	33.715 (26.406 to 41.966)	224 (178 to 282)	38.617 (30.792 to 48.367)	0.45<br>(0.44 to 0.47)
Bolivia (Plurinational State of)	899 (704 to 1120)	29.621 (23.586 to 37.022)	2690 (2153 to 3286)	30.486 (24.409 to 37.107)	0.10<br>(-0.05 to 0.25)
Bosnia and Herzegovina	11073 (9023 to 13570)	294.512 (242.497 to 358.216)	25517 (21059 to 31070)	394.253 (326.341 to 476.904)	0.94

					(0.82 to 1.07)
Botswana	87 (68 to 109)	14.670 (11.584 to 18.210)	252 (201 to 310)	15.832 (12.773 to 19.417)	0.26<br>(0.19 to 0.32)
Brazil	39497 (36329 to 42752)	45.233 (41.663 to 49.007)	109273 (101497 to 118081)	43.764 (40.720 to 47.199)	-0.09<br>(-0.19 to 0)
Brunei Darussalam	368 (296 to 448)	398.093 (314.842 to 486.629)	1272 (1064 to 1518)	410.115 (343.712 to 488.549)	0.09<br>(0.04 to 0.14)
Bulgaria	30096 (23422 to 38190)	240.002 (187.749 to 301.266)	35213 (28412 to 43119)	234.039 (189.293 to 285.650)	-0.08<br>(-0.1 to -0.07)
Burkina Faso	536 (427 to 687)	12.091 (9.596 to 15.205)	1292 (1027 to 1624)	13.116 (10.378 to 16.507)	0.28<br>(0.24 to 0.32)
Burundi	367 (294 to 452)	15.671 (12.544 to 19.362)	555 (434 to 682)	11.470 (9.019 to 14.159)	-1.00<br>(-1.06 to -0.94)
Cabo Verde	29 (22 to 36)	12.892 (10.051 to 16.110)	74 (60 to 94)	15.840 (12.643 to 19.968)	0.68<br>(0.65 to 0.71)
Cambodia	2462 (1920 to 3093)	65.969 (52.097 to 81.902)	7827 (6198 to 9641)	72.429 (57.719 to 89.722)	0.30<br>(0.29 to 0.31)
Cameroon	675 (541 to 842)	14.521 (11.701 to 18.173)	2021 (1609 to 2525)	14.551 (11.547 to 18.114)	0.01<br>(-0.03 to 0.05)
Canada	156152 (130551 to 191655)	463.657 (388.162 to 568.707)	316688 (262897 to 390392)	414.206 (345.229 to 508.264)	-0.36<br>(-0.37 to -0.35)
Central African Republic	159 (123 to 200)	14.848 (11.562 to 18.502)	258 (204 to 324)	12.429 (9.881 to 15.346)	-0.56<br>(-0.67 to -0.45)
Chad	336 (263 to 424)	11.738 (9.192 to 14.686)	833 (661 to 1042)	13.313 (10.575 to 16.613)	0.40<br>(0.37 to 0.43)
Chile	14315 (11828 to 17395)	147.789 (121.953 to 178.896)	40269 (33282 to 48818)	154.176 (127.618 to 186.362)	0.11

					(-0.03 to 0.25)
China	799331 (746632 to 856146)	110.752 (103.652 to 118.023)	2598301 (2472060 to 2729052)	123.874 (117.747 to 130.133)	0.35<br>(0.25 to 0.44)
Colombia	7955 (6343 to 9753)	47.728 (38.452 to 58.957)	32369 (27201 to 38043)	58.774 (49.552 to 68.977)	0.71<br>(0.61 to 0.82)
Comoros	32 (26 to 40)	16.528 (13.196 to 20.305)	64 (51 to 80)	13.308 (10.542 to 16.334)	-0.70<br>(-0.72 to -0.67)
Congo	182 (144 to 224)	17.643 (13.949 to 21.769)	420 (342 to 521)	16.277 (13.256 to 19.942)	-0.25<br>(-0.32 to -0.17)
Cook Islands	9 (7 to 11)	78.144 (61.766 to 98.557)	20 (15 to 25)	73.755 (58.194 to 93.754)	-0.18<br>(-0.23 to -0.13)
Costa Rica	567 (450 to 711)	33.048 (26.263 to 41.380)	1831 (1452 to 2299)	33.388 (26.531 to 41.745)	0.02<br>(-0.32 to 0.37)
Croatia	607 (476 to 768)	363.135 (294.798 to 434.771)	1764 (1404 to 2233)	459.156 (379.313 to 581.168)	0.76<br>(0.62 to 0.91)
Cuba	21579 (17422 to 26028)	35.949 (28.849 to 44.043)	43290 (35915 to 55132)	38.051 (31.673 to 46.089)	0.19<br>(0.15 to 0.23)
Cyprus	3709 (2979 to 4540)	79.742 (65.094 to 94.500)	7365 (6075 to 8977)	77.120 (64.348 to 92.096)	-0.13<br>(-0.21 to -0.04)
Czechia	708 (579 to 838)	356.629 (288.019 to 435.576)	1726 (1439 to 2068)	486.565 (406.729 to 598.642)	1.04<br>(0.89 to 1.19)
Côte d'Ivoire	50311 (40518 to 61632)	13.924 (11.031 to 17.687)	111717 (93959 to 137734)	14.147 (11.169 to 17.724)	0.05<br>(0 to 0.10)
Democratic People's Republic of Korea	12437 (9898 to 15421)	95.454 (75.853 to 119.030)	26864 (21300 to 33311)	86.369 (68.486 to 107.316)	-0.32<br>(-0.35 to -0.30)
Democratic Republic of the	2267 (1747 to 2840)	15.232 (11.971 to 18.848)	4477 (3512 to 5549)	12.512 (9.848 to 15.413)	-0.62

Congo					(-0.69 to -0.56)
Denmark	11081 (9285 to 13362)	126.192 (105.534 to 151.825)	17242 (14564 to 20346)	131.885 (111.145 to 155.369)	0.15 (-0.01 to 0.32)
Djibouti	22 (17 to 27)	16.133 (12.870 to 20.007)	92 (71 to 116)	14.611 (11.460 to 17.949)	-0.31 (-0.35 to -0.28)
Dominica	25 (20 to 29)	40.819 (32.716 to 48.678)	40 (32 to 49)	47.336 (38.256 to 57.196)	0.48 (0.36 to 0.6)
Dominican Republic	1232 (978 to 1534)	34.019 (27.120 to 42.526)	3619 (2896 to 4396)	36.570 (29.292 to 44.274)	0.26 (0.22 to 0.30)
Ecuador	1622 (1290 to 2000)	31.693 (25.047 to 39.355)	5717 (4534 to 6879)	35.113 (27.915 to 42.286)	0.36 (0.18 to 0.53)
Egypt	14795 (11875 to 17666)	65.316 (52.693 to 79.026)	37001 (29929 to 44646)	69.226 (56.950 to 84.300)	0.17 (0.12 to 0.22)
El Salvador	783 (617 to 987)	27.166 (21.248 to 34.340)	1626 (1251 to 2041)	26.065 (20.028 to 32.951)	-0.10 (-0.22 to 0.01)
Equatorial Guinea	30 (23 to 37)	15.814 (12.573 to 19.413)	95 (76 to 117)	19.198 (15.417 to 23.713)	0.65 (0.60 to 0.71)
Eritrea	161 (126 to 203)	14.571 (11.504 to 17.935)	353 (280 to 437)	12.968 (10.371 to 15.869)	-0.37 (-0.40 to -0.35)
Estonia	5671 (4558 to 6965)	273.906 (220.731 to 336.097)	10442 (8463 to 12812)	365.151 (299.772 to 444.050)	0.94 (0.88 to 1.00)
Eswatini	41 (32 to 52)	13.524 (10.647 to 16.769)	83 (65 to 103)	13.852 (11.035 to 17.231)	0.08 (0.03 to 0.13)
Ethiopia	2697 (2419 to 2976)	13.808 (12.542 to 15.100)	4957 (4453 to 5498)	11.522 (10.437 to 12.667)	-0.57 (-0.61 to -0.53)
Fiji	210 (164 to 263)	73.263 (58.133 to 92.423)	498 (389 to 623)	74.124 (58.283 to 92.067)	0.04

					(0.01 to 0.08)
Finland	22859 (18973 to 27458)	303.295 (251.057 to 364.479)	45552 (37318 to 54893)	323.734 (265.949 to 386.651)	0.19<br>(0.07 to 0.31)
France	128520 (105336 to 157914)	147.073 (120.608 to 180.425)	160777 (126368 to 198447)	108.728 (85.703 to 134.171)	-0.97<br>(-1.01 to -0.93)
Gabon	119 (93 to 148)	21.030 (16.522 to 26.224)	190 (152 to 236)	18.609 (14.843 to 22.711)	-0.39<br>(-0.47 to -0.31)
Gambia	46 (36 to 57)	12.292 (9.694 to 15.333)	135 (108 to 169)	12.823 (10.151 to 16.047)	0.15<br>(0.09 to 0.20)
Georgia	27282 (21614 to 35081)	445.922 (353.524 to 567.619)	63551 (53898 to 75022)	1013.767 (860.903 to 1194.241)	2.69<br>(2.57 to 2.81)
Germany	190314 (160903 to 228313)	142.54 (120.225 to 170.060)	373801 (306801 to 444547)	178.094 (146.609 to 211.010)	0.75<br>(0.58 to 0.91)
Ghana	904 (714 to 1134)	13.635 (10.696 to 16.940)	2592 (2074 to 3220)	14.240 (11.347 to 18.116)	0.15<br>(0.12 to 0.19)
Greece	13468 (11111 to 16195)	84.999 (70.127 to 102.029)	35393 (28517 to 46381)	137.948 (112.399 to 182.933)	1.56<br>(1.51 to 1.61)
Greenland	162 (134 to 194)	607.667 (497.870 to 736.232)	377 (301 to 453)	605.583 (488.192 to 732.339)	0<br>(-0.02 to 0.01)
Grenada	34 (26 to 42)	46.189 (35.967 to 56.909)	74 (61 to 86)	63.780 (52.999 to 73.907)	1.05<br>(0.85 to 1.25)
Guam	64 (51 to 80)	103.870 (81.884 to 129.844)	180 (145 to 227)	84.593 (68.419 to 106.251)	-0.65<br>(-0.70 to -0.60)
Guatemala	918 (706 to 1165)	29.080 (22.718 to 37.029)	2922 (2257 to 3734)	27.277 (21.053 to 34.693)	-0.18<br>(-0.33 to -0.02)
Guinea	456 (361 to 575)	13.601 (10.708 to 17.031)	853 (685 to 1054)	14.217 (11.386 to 17.472)	0.16

					(0.12 to 0.20)
Guinea-Bissau	55 (43 to 69)	13.356 (10.589 to 16.572)	107 (86 to 134)	13.307 (10.682 to 16.531)	-0.01<br>(-0.05 to 0.03)
Guyana	177 (146 to 215)	47.558 (38.579 to 57.798)	305 (247 to 364)	48.336 (39.536 to 57.370)	0.06<br>(0.02 to 0.11)
Haiti	1196 (953 to 1464)	39.224 (31.160 to 48.348)	2449 (1962 to 2991)	36.204 (29.126 to 43.824)	-0.25<br>(-0.38 to -0.12)
Honduras	538 (420 to 680)	27.984 (21.868 to 35.195)	1763 (1385 to 2216)	29.250 (23.052 to 36.144)	0.16<br>(-0.10 to 0.41)
Hungary	58365 (48532 to 69893)	385.505 (322.056 to 461.284)	83485 (69389 to 100358)	404.347 (336.497 to 484.399)	0.14<br>(0.09 to 0.20)
Iceland	305 (249 to 371)	102.130 (83.251 to 123.922)	553 (446 to 676)	91.668 (73.972 to 111.093)	-0.36<br>(-0.42 to -0.29)
India	130781 (118935 to 143260)	33.524 (30.807 to 36.348)	396864 (364587 to 431478)	35.568 (32.865 to 38.494)	0.21<br>(0.11 to 0.31)
Indonesia	57790 (53084 to 63373)	72.683 (67.149 to 78.844)	165781 (152150 to 181286)	81.658 (75.692 to 88.435)	0.37<br>(0.35 to 0.39)
Iran (Islamic Republic of)	13398 (12295 to 14655)	59.787 (55.459 to 64.647)	42516 (39455 to 46155)	59.581 (55.329 to 64.492)	-0.02<br>(-0.06 to 0.02)
Iraq	3687 (2920 to 4564)	49.654 (39.446 to 61.761)	10163 (8151 to 12724)	47.723 (38.325 to 60.042)	-0.11<br>(-0.14 to -0.08)
Ireland	6855 (5647 to 8425)	155.104 (127.214 to 189.721)	12625 (10309 to 15241)	151.369 (123.877 to 182.096)	-0.07<br>(-0.12 to -0.03)
Israel	10183 (8379 to 12342)	198.714 (163.028 to 240.992)	22952 (19075 to 27554)	175.361 (145.443 to 210.235)	-0.40<br>(-0.42 to -0.39)
Italy	756052 (722135 to 791331)	807.463 (771.238 to 844.824)	1466416 (1392644 to 1532945)	924.860 (882.157 to 966.441)	0.45

					(0.40 to 0.50)
Jamaica	575 (458 to 709)	31.687 (25.087 to 39.408)	975 (785 to 1209)	31.462 (25.352 to 39.042)	-0.01<br>(-0.07 to 0.05)
Japan	680585 (636809 to 730824)	398.838 (373.214 to 427.349)	1650169 (1573292 to 1730896)	420.380 (402.863 to 441.937)	0.17<br>(0.15 to 0.19)
Jordan	564 (443 to 709)	47.769 (37.796 to 59.971)	2989 (2370 to 3711)	43.451 (34.985 to 53.458)	-0.30<br>(-0.34 to -0.27)
Kazakhstan	42560 (33615 to 52810)	363.625 (287.847 to 452.366)	68050 (54236 to 83620)	406.662 (328.079 to 496.018)	0.36<br>(0.34 to 0.38)
Kenya	1125 (1020 to 1239)	13.563 (12.370 to 14.843)	3021 (2722 to 3336)	13.006 (11.866 to 14.233)	-0.13<br>(-0.16 to -0.10)
Kiribati	24 (19 to 30)	77.720 (61.408 to 96.053)	48 (37 to 59)	80.356 (63.041 to 99.078)	0.12<br>(0.08 to 0.17)
Kuwait	322 (261 to 390)	59.436 (47.968 to 73.406)	1483 (1194 to 1824)	55.149 (43.868 to 68.560)	-0.24<br>(-0.29 to -0.19)
Kyrgyzstan	10390 (8247 to 12757)	369.254 (293.967 to 454.134)	15572 (12686 to 19308)	366.145 (298.007 to 450.126)	-0.02<br>(-0.04 to -0.01)
Lao People's Democratic Republic	1251 (989 to 1548)	71.118 (56.538 to 87.264)	3160 (2500 to 3886)	81.633 (65.197 to 100.991)	0.44<br>(0.39 to 0.49)
Latvia	8073 (6512 to 10038)	223.444 (180.625 to 276.339)	9974 (7958 to 12763)	240.635 (192.825 to 303.199)	0.21<br>(0.02 to 0.40)
Lebanon	1269 (1033 to 1560)	63.871 (52.641 to 78.355)	3643 (2980 to 4363)	59.261 (48.365 to 71.056)	-0.24<br>(-0.26 to -0.23)
Lesotho	99 (77 to 124)	11.411 (8.956 to 14.130)	155 (122 to 196)	13.860 (11.100 to 17.322)	0.64<br>(0.62 to 0.67)
Liberia	148 (117 to 185)	12.599 (9.896 to 15.716)	273 (219 to 349)	11.314 (8.988 to 14.441)	-0.33

					(-0.37 to -0.29)
Libya	1142 (909 to 1413)	66.507 (53.237 to 82.077)	2607 (2113 to 3186)	56.501 (45.797 to 69.261)	-0.53<br>(-0.59 to -0.47)
Lithuania	12659 (10050 to 15857)	279.928 (222.564 to 348.214)	18853 (15160 to 23338)	313.005 (252.604 to 388.023)	0.38<br>(0.27 to 0.49)
Luxembourg	1980 (1664 to 2420)	345.354 (289.556 to 422.023)	3520 (2884 to 4271)	326.054 (267.818 to 394.748)	-0.18<br>(-0.39 to 0.03)
Madagascar	884 (704 to 1097)	17.256 (13.975 to 21.323)	1721 (1377 to 2125)	15.096 (12.152 to 18.633)	-0.43<br>(-0.48 to -0.38)
Malawi	517 (403 to 652)	13.709 (10.782 to 17.132)	903 (707 to 1134)	12.152 (9.597 to 15.157)	-0.38<br>(-0.41 to -0.36)
Malaysia	6785 (5435 to 8391)	83.770 (66.883 to 103.720)	26169 (21254 to 32590)	99.438 (81.161 to 124.456)	0.56<br>(0.54 to 0.58)
Maldives	66 (51 to 83)	95.149 (74.947 to 118.210)	317 (256 to 384)	114.599 (93.056 to 139.884)	0.58<br>(0.51 to 0.66)
Mali	460 (359 to 577)	11.301 (8.926 to 14.275)	1094 (857 to 1386)	11.813 (9.237 to 14.932)	0.15<br>(0.11 to 0.18)
Malta	432 (362 to 519)	99.471 (83.306 to 119.590)	1007 (836 to 1242)	93.256 (77.718 to 114.782)	-0.20<br>(-0.33 to -0.07)
Marshall Islands	11 (9 to 14)	77.492 (61.791 to 98.163)	22 (18 to 27)	79.751 (64.798 to 97.822)	0.10<br>(0.05 to 0.14)
Mauritania	149 (120 to 186)	14.407 (11.547 to 18.064)	311 (245 to 389)	13.652 (10.831 to 16.896)	-0.16<br>(-0.25 to -0.08)
Mauritius	554 (454 to 686)	84.508 (69.290 to 103.526)	1512 (1218 to 1887)	82.695 (67.102 to 102.349)	-0.07<br>(-0.09 to -0.05)
Mexico	13304 (12164 to 14482)	32.994 (30.265 to 35.880)	37709 (34543 to 41162)	30.487 (27.954 to 33.098)	-0.28

					(-0.37 to -0.20)
Micronesia (Federated States of)	34 (27 to 43)	77.196 (61.423 to 96.256)	45 (36 to 58)	75.094 (59.739 to 93.386)	-0.08<br>(-0.11 to -0.05)
Monaco	86 (69 to 107)	112.601 (90.362 to 141.139)	123 (99 to 152)	116.868 (94.296 to 143.196)	0.12<br>(0.10 to 0.13)
Mongolia	5318 (4166 to 6588)	540.489 (423.361 to 672.875)	11862 (9620 to 14345)	596.927 (481.761 to 723.967)	0.31<br>(0.21 to 0.41)
Montenegro	1426 (1151 to 1802)	235.423 (190.644 to 294.923)	2528 (2061 to 3119)	251.093 (206.588 to 308.863)	0.22<br>(0.15 to 0.29)
Morocco	7952 (6369 to 9776)	59.562 (47.898 to 74.017)	20054 (16370 to 24298)	62.172 (51.228 to 74.184)	0.14<br>(0.12 to 0.16)
Mozambique	740 (570 to 930)	12.626 (9.777 to 15.838)	1499 (1178 to 1839)	13.491 (10.659 to 16.688)	0.23<br>(0.17 to 0.30)
Myanmar	13333 (10387 to 16709)	68.031 (53.801 to 84.631)	34234 (26911 to 42382)	78.693 (62.524 to 97.209)	0.47<br>(0.44 to 0.50)
Namibia	104 (81 to 127)	15.258 (11.955 to 18.648)	216 (172 to 272)	14.766 (11.683 to 18.457)	-0.09<br>(-0.12 to -0.07)
Nauru	4 (3 to 4)	102.510 (82.265 to 127.88)	4 (3 to 5)	82.362 (66.476 to 102.543)	-0.70<br>(-0.72 to -0.67)
Nepal	2567 (1968 to 3232)	32.166 (25.027 to 40.575)	7582 (6002 to 9538)	34.699 (27.542 to 44.136)	0.26<br>(0.21 to 0.31)
Netherlands	48268 (39219 to 58908)	228.141 (185.465 to 279.559)	107450 (87720 to 129834)	277.530 (227.789 to 335.783)	0.63<br>(0.59 to 0.67)
New Zealand	11482 (10845 to 12198)	277.478 (262.115 to 294.574)	27697 (26256 to 29274)	306.368 (290.694 to 323.680)	0.32<br>(0.27 to 0.36)
Nicaragua	431 (341 to 534)	29.476 (23.326 to 36.592)	1322 (1038 to 1629)	27.876 (21.630 to 34.531)	-0.23

					(-0.44 to -0.02)
Niger	351 (276 to 448)	11.870 (9.335 to 15.096)	994 (799 to 1261)	11.287 (8.937 to 14.305)	-0.17<br>(-0.22 to -0.11)
Nigeria	6245 (5709 to 6826)	13.588 (12.491 to 14.771)	13132 (11948 to 14434)	13.025 (11.950 to 14.115)	-0.15<br>(-0.20 to -0.10)
Niue	2 (1 to 2)	77.650 (61.153 to 97.115)	2 (1 to 2)	75.950 (60.223 to 95.182)	-0.06<br>(-0.12 to 0)
North Macedonia	4416 (3570 to 5449)	248.777 (202.457 to 305.282)	8259 (6723 to 10183)	244.51 (200.131 to 301.439)	-0.05<br>(-0.07 to -0.04)
Northern Mariana Islands	11 (9 to 14)	93.060 (72.254 to 116.921)	37 (29 to 47)	84.066 (66.014 to 106.593)	-0.32<br>(-0.38 to -0.26)
Norway	64933 (61711 to 68214)	853.129 (811.465 to 895.811)	95424 (90477 to 100580)	883.241 (839.582 to 929.201)	0.13<br>(0.03 to 0.23)
Oman	319 (252 to 402)	52.411 (40.584 to 66.227)	999 (812 to 1217)	57.501 (46.026 to 70.932)	0.30<br>(0.25 to 0.34)
Pakistan	18167 (16560 to 19783)	34.957 (32.089 to 37.961)	40479 (37073 to 44287)	38.221 (35.190 to 41.593)	0.29<br>(0.28 to 0.30)
Palau	8 (6 to 10)	89.908 (70.529 to 112.734)	17 (13 to 21)	86.043 (70.484 to 105.190)	-0.14<br>(-0.18 to -0.09)
Palestine	383 (301 to 471)	46.425 (36.453 to 57.317)	1037 (831 to 1261)	44.160 (35.284 to 54.419)	-0.16<br>(-0.18 to -0.14)
Panama	544 (431 to 675)	37.391 (29.660 to 46.371)	1522 (1205 to 1914)	34.510 (27.311 to 43.425)	-0.23<br>(-0.34 to -0.12)
Papua New Guinea	958 (739 to 1219)	68.819 (53.841 to 86.488)	2701 (2145 to 3414)	69.147 (55.143 to 86.064)	0.02<br>(-0.01 to 0.06)
Paraguay	807 (642 to 981)	36.725 (28.991 to 44.856)	2474 (2027 to 2968)	42.821 (35.185 to 51.285)	0.52

					(0.48 to 0.55)
Peru	2861 (2241 to 3608)	24.677 (19.233 to 31.313)	7843 (6142 to 9807)	23.563 (18.505 to 29.510)	-0.12 (-0.4 to 0.15)
Philippines	17300 (15887 to 18972)	69.875 (64.256 to 75.730)	50289 (46296 to 55046)	69.863 (64.827 to 75.755)	0.01 (-0.03 to 0.04)
Poland	61800 (58036 to 66192)	141.073 (132.643 to 150.742)	109778 (104726 to 115197)	147.418 (141.042 to 154.671)	0.14 (0.10 to 0.19)
Portugal	8472 (6987 to 10131)	57.441 (47.492 to 68.837)	19752 (16454 to 25743)	75.253 (62.600 to 97.802)	0.83 (0.69 to 0.97)
Puerto Rico	1430 (1137 to 1780)	38.948 (31.057 to 48.443)	2552 (2017 to 3162)	35.720 (28.469 to 43.536)	-0.27 (-0.28 to -0.25)
Qatar	77 (60 to 93)	89.712 (70.845 to 110.589)	538 (421 to 671)	64.902 (52.702 to 80.591)	-1.04 (-1.17 to -0.92)
Republic of Korea	69578 (56855 to 86346)	253.788 (207.406 to 314.245)	260543 (213528 to 315746)	273.758 (225.772 to 329.976)	0.25 (0.23 to 0.26)
Republic of Moldova	10466 (8445 to 12806)	249.129 (203.418 to 304.688)	14456 (11522 to 17757)	238.298 (190.801 to 291.580)	-0.14 (-0.18 to -0.10)
Romania	73198 (59577 to 90153)	264.870 (216.145 to 322.507)	103388 (86031 to 125370)	266.559 (220.772 to 321.865)	0.02 (-0.01 to 0.05)
Russian Federation	433531 (399210 to 473948)	243.963 (224.981 to 265.168)	670197 (621491 to 727516)	272.369 (253.373 to 294.521)	0.36 (0.33 to 0.39)
Rwanda	498 (402 to 618)	17.533 (14.033 to 21.464)	848 (665 to 1063)	13.538 (10.676 to 16.874)	-0.83 (-0.86 to -0.80)
Saint Kitts and Nevis	20 (16 to 24)	50.010 (40.195 to 60.454)	33 (26 to 41)	48.867 (39.755 to 58.886)	-0.07 (-0.16 to 0.01)
Saint Lucia	31 (24 to 39)	34.945 (27.408 to 43.808)	79 (63 to 99)	33.031 (26.427 to 41.223)	-0.17

					(-0.21 to -0.12)
Saint Vincent and the Grenadines	32 (25 to 39)	43.391 (34.288 to 53.297)	69 (56 to 83)	47.736 (39.242 to 57.725)	0.32<br>(0.29 to 0.35)
Samoa	58 (45 to 72)	76.192 (60.376 to 95.817)	98 (78 to 120)	75.020 (60.208 to 93.397)	-0.06<br>(-0.09 to -0.02)
San Marino	51 (40 to 62)	135.148 (107.773 to 164.370)	115 (93 to 143)	145.213 (117.424 to 180.479)	0.23<br>(0.21 to 0.25)
Sao Tome and Principe	8 (6 to 10)	12.167 (9.447 to 15.278)	17 (13 to 21)	13.370 (10.687 to 16.848)	0.32<br>(0.28 to 0.36)
Saudi Arabia	3719 (3038 to 4450)	72.646 (59.698 to 87.902)	12192 (10009 to 14714)	77.461 (64.227 to 93.747)	0.21<br>(0.18 to 0.25)
Senegal	410 (321 to 523)	12.267 (9.539 to 15.469)	1020 (815 to 1287)	12.399 (9.769 to 15.701)	0.06<br>(0 to 0.12)
Serbia	48510 (39449 to 58378)	442.933 (363.391 to 528.348)	90102 (74943 to 105191)	512.972 (426.213 to 602.413)	0.54<br>(0.31 to 0.76)
Seychelles	60 (48 to 74)	104.260 (83.762 to 128.655)	126 (102 to 153)	119.235 (97.096 to 144.942)	0.43<br>(0.4 to 0.46)
Sierra Leone	245 (193 to 307)	11.681 (9.170 to 14.670)	482 (386 to 607)	11.763 (9.244 to 14.857)	0.03<br>(0 to 0.05)
Singapore	7274 (5976 to 9066)	347.991 (287.257 to 433.741)	26188 (21548 to 31826)	304.042 (250.002 to 368.410)	-0.44<br>(-0.48 to -0.41)
Slovakia	10533 (8506 to 13070)	174.360 (141.340 to 216.626)	18818 (15549 to 22456)	191.365 (158.712 to 227.513)	0.32<br>(0.20 to 0.44)
Slovenia	12159 (9798 to 14621)	493.572 (399.083 to 592.153)	23199 (18908 to 28351)	499.792 (409.388 to 605.930)	0.05<br>(-0.04 to 0.15)
Solomon Islands	81 (63 to 102)	75.959 (59.716 to 95.920)	197 (154 to 242)	70.732 (55.551 to 88.349)	-0.21

					(-0.24 to -0.19)
Somalia	274 (217 to 341)	11.809 (9.097 to 14.587)	548 (424 to 683)	8.910 (6.974 to 11.040)	-0.9<br>(-0.94 to -0.85)
South Africa	3122 (2876 to 3401)	14.339 (13.232 to 15.559)	5912 (5376 to 6531)	12.262 (11.236 to 13.435)	-0.5<br>(-0.57 to -0.42)
South Sudan	435 (345 to 539)	17.032 (13.670 to 21.230)	469 (366 to 582)	12.568 (9.805 to 15.512)	-0.98<br>(-1.01 to -0.95)
Spain	60815 (50835 to 73326)	104.826 (87.743 to 126.505)	142532 (116973 to 195126)	139.382 (114.194 to 193.096)	0.88<br>(0.80 to 0.96)
Sri Lanka	9637 (7726 to 12089)	104.105 (83.957 to 129.818)	32332 (26195 to 39400)	120.403 (97.858 to 146.715)	0.49<br>(0.43 to 0.55)
Sudan	4693 (3766 to 5670)	54.333 (43.712 to 66.350)	9231 (7455 to 11097)	53.345 (43.166 to 64.874)	-0.06<br>(-0.07 to -0.04)
Suriname	121 (99 to 146)	49.066 (39.895 to 59.064)	313 (257 to 379)	49.947 (41.321 to 60.426)	0.06<br>(0.03 to 0.09)
Sweden	43125 (40231 to 46309)	256.282 (239.525 to 274.361)	69622 (64719 to 74784)	292.136 (272.397 to 316.098)	0.40<br>(0.34 to 0.46)
Switzerland	32375 (26877 to 40090)	290.56 (239.389 to 357.378)	52238 (42102 to 62861)	270.906 (219.918 to 327.294)	-0.25<br>(-0.36 to -0.14)
Syrian Arab Republic	4021 (3114 to 4912)	88.053 (67.718 to 109.693)	10207 (8094 to 12439)	87.373 (70.227 to 107.297)	-0.02<br>(-0.06 to 0.03)
Taiwan (Province of China)	23460 (18538 to 29041)	166.051 (131.496 to 206.271)	72802 (58848 to 88724)	166.495 (134.961 to 202.697)	0.01<br>(-0.03 to 0.05)
Tajikistan	9426 (7456 to 11757)	374.330 (297.913 to 469.350)	18784 (14960 to 23747)	390.020 (310.450 to 485.356)	0.13<br>(0.08 to 0.18)
Thailand	23895 (18609 to 29861)	79.083 (62.177 to 99.117)	100099 (79333 to 123096)	91.613 (72.789 to 112.771)	0.47

					(0.44 to 0.51)
Timor-Leste	147 (115 to 185)	73.159 (57.584 to 91.487)	652 (514 to 809)	80.793 (63.555 to 99.918)	0.31<br>(0.26 to 0.36)
Togo	163 (130 to 204)	12.301 (9.692 to 15.527)	522 (422 to 656)	12.250 (9.866 to 15.355)	-0.01<br>(-0.06 to 0.04)
Tokelau	1 (1 to 1)	77.268 (61.173 to 95.955)	1 (1 to 1)	74.008 (58.947 to 91.607)	-0.12<br>(-0.20 to -0.05)
Tonga	37 (29 to 46)	73.468 (58.209 to 92.393)	54 (43 to 68)	70.145 (55.802 to 87.621)	-0.15<br>(-0.20 to -0.10)
Trinidad and Tobago	328 (264 to 400)	38.649 (31.167 to 47.436)	666 (529 to 843)	34.208 (27.214 to 42.995)	-0.38<br>(-0.46 to -0.30)
Tunisia	2893 (2297 to 3528)	61.534 (49.277 to 75.530)	7660 (6159 to 9321)	59.372 (47.730 to 72.261)	-0.12<br>(-0.15 to -0.10)
Turkey	28700 (23729 to 34385)	88.985 (72.875 to 107.081)	71931 (59887 to 87359)	77.758 (64.916 to 94.322)	-0.45<br>(-0.62 to -0.28)
Turkmenistan	6476 (5109 to 8128)	376.679 (299.899 to 474.619)	14474 (11484 to 18552)	409.671 (330.847 to 519.177)	0.26<br>(0.22 to 0.31)
Tuvalu	4 (3 to 5)	71.644 (56.031 to 89.671)	7 (6 to 9)	71.725 (57.752 to 89.283)	0.01<br>(-0.07 to 0.08)
Uganda	832 (648 to 1027)	12.907 (10.025 to 16.138)	1918 (1500 to 2367)	12.801 (10.007 to 15.649)	-0.02<br>(-0.04 to 0)
Ukraine	154290 (141326 to 168545)	215.205 (197.892 to 234.556)	175230 (162440 to 190531)	218.263 (202.459 to 236.931)	0.05<br>(-0.04 to 0.15)
United Arab Emirates	326 (264 to 390)	90.250 (72.383 to 110.107)	2592 (2035 to 3229)	74.829 (61.016 to 92.409)	-0.6<br>(-0.64 to -0.56)
United Kingdom	227815 (214240 to 242215)	233.036 (219.644 to 248.926)	400856 (378160 to 422861)	283.424 (267.709 to 298.834)	0.63

					(0.61 to 0.66)
United Republic of Tanzania	1686 (1317 to 2073)	15.584 (12.336 to 19.403)	3799 (3017 to 4702)	14.814 (11.839 to 18.259)	-0.15 (-0.17 to -0.12)
United States of America	1786377 (1691259 to 1885120)	527.707 (500.188 to 556.969)	3615215 (3442691 to 3783700)	581.253 (554.343 to 608.598)	0.33 (0.30 to 0.37)
United States Virgin Islands	50 (41 to 61)	60.498 (49.787 to 73.537)	127 (105 to 156)	66.577 (55.050 to 80.947)	0.32 (0.28 to 0.36)
Uruguay	5655 (4586 to 7060)	139.289 (112.863 to 173.723)	9344 (7715 to 11574)	161.709 (132.646 to 199.014)	0.46 (0.41 to 0.52)
Uzbekistan	38300 (30260 to 47518)	354.062 (280.185 to 443.88)	112860 (90907 to 141948)	503.572 (411.487 to 625.747)	1.18 (1.10 to 1.26)
Vanuatu	37 (29 to 47)	74.823 (58.832 to 93.063)	101 (79 to 124)	70.269 (55.557 to 87.931)	-0.20 (-0.26 to -0.14)
Venezuela (Bolivarian Republic of)	3226 (2560 to 4046)	34.943 (27.690 to 43.924)	10636 (8541 to 13133)	35.910 (28.845 to 44.247)	0.09 (-0.04 to 0.22)
Viet Nam	25723 (20525 to 32060)	68.138 (54.268 to 84.584)	70041 (55587 to 87512)	78.392 (62.810 to 97.575)	0.45 (0.44 to 0.47)
Yemen	2404 (1932 to 2932)	56.416 (45.222 to 68.4900)	6642 (5351 to 7975)	53.513 (43.224 to 64.608)	-0.16 (-0.18 to -0.15)
Zambia	447 (350 to 556)	16.111 (12.637 to 20.005)	1119 (910 to 1367)	16.473 (13.334 to 19.850)	0.08 (0.05 to 0.10)
Zimbabwe	580 (442 to 728)	13.593 (10.357 to 16.942)	980 (764 to 1237)	13.280 (10.510 to 16.494)	-0.06 (-0.08 to -0.03)

Abbreviations: AAPC, annual percentage change; CI, confidence interval; DMVD, degenerative mitral valve disease; UI, uncertain interval.

Supplementary Table 2: Age-standardized mortality rates and average annual percentage change for DMVD at national level, 1990-2021

	1990		2021		1990 – 2021
**Nation**	**Cases (95% UI)**	**Age-standardized mortality per 100 000 population (95% UI)**	**Cases (95% UI)**	**Age- standardized mortality per 100 000 population (95% UI)**	**AAPC % (95% CI)**
Afghanistan	82 (39 to 147)	1.243 (0.645 to 2.147)	121 (54 to 217)	1.112 (0.559 to 1.872)	-0.35 (-0.43 to -0.27)
Albania	16 (11 to 21)	0.873 (0.620 to 1.199)	31 (18 to 49)	0.739 (0.434 to 1.162)	-0.64 (-1.08 to -0.19)
Algeria	117 (74 to 193)	1.069 (0.685 to 1.749)	261 (159 to 468)	0.836 (0.503 to 1.508)	-0.77 (-0.89 to -0.66)
American Samoa	0 (0 to 0)	0.291 (0.165 to 0.379)	0 (0 to 0)	0.151 (0.110 to 0.225)	-2.00 (-2.33 to -1.67)
Andorra	0 (0 to 0)	0.645 (0.379 to 1.007)	1 (0 to 1)	0.348 (0.200 to 0.606)	-2.04 (-2.39 to -1.67)
Angola	33 (18 to 49)	0.922 (0.537 to 1.347)	84 (51 to 132)	0.802 (0.491 to 1.325)	-0.45 (-0.66 to -0.25)
Antigua and Barbuda	0 (0 to 0)	0.626 (0.569 to 0.683)	0 (0 to 1)	0.484 (0.447 to 0.523)	-0.69 (-2.20 to 0.83)
Argentina	165 (141 to 187)	0.535 (0.457 to 0.607)	187 (168 to 205)	0.325 (0.294 to 0.356)	-1.46 (-2.34 to -0.58)
Armenia	3 (2 to 4)	0.119 (0.097 to 0.145)	11 (9 to 13)	0.249 (0.209 to 0.292)	2.03 (0.63 to 3.45)
Australia	196 (176 to 211)	1.058 (0.943 to 1.142)	278 (229 to 316)	0.527 (0.442 to 0.594)	-2.11 (-2.98 to -1.23)
Austria	137 (124 to 147)	1.134 (1.029 to 1.220)	212 (172 to 239)	0.934 (0.774 to 1.040)	-0.50

					(-1.67 to 0.69)
Azerbaijan	2 (1 to 2)	0.035 (0.025 to 0.048)	3 (2 to 4)	0.032 (0.023 to 0.046)	-0.20<br>(-0.80 to 0.41)
Bahamas	1 (1 to 1)	0.341 (0.299 to 0.381)	2 (1 to 2)	0.382 (0.314 to 0.473)	0.15<br>(-0.55 to 0.85)
Bahrain	1 (1 to 2)	0.704 (0.480 to 0.943)	3 (2 to 4)	0.360 (0.218 to 0.478)	-2.22<br>(-2.74 to -1.68)
Bangladesh	183 (102 to 269)	0.404 (0.228 to 0.592)	451 (276 to 687)	0.374 (0.228 to 0.572)	-0.07<br>(-0.66 to 0.51)
Barbados	1 (0 to 1)	0.184 (0.164 to 0.205)	1 (1 to 1)	0.171 (0.136 to 0.211)	-0.04<br>(-0.80 to 0.74)
Belarus	1 (1 to 2)	0.011 (0.008 to 0.014)	6 (5 to 7)	0.037 (0.030 to 0.046)	4.05<br>(3.41 to 4.69)
Belgium	371 (325 to 406)	2.354 (2.069 to 2.568)	204 (163 to 230)	0.686 (0.566 to 0.764)	-3.94<br>(-4.60 to -3.28)
Belize	0 (0 to 1)	0.415 (0.366 to 0.507)	2 (1 to 2)	0.485 (0.427 to 0.549)	0.69<br>(-0.43 to 1.82)
Benin	11 (7 to 17)	0.568 (0.332 to 0.882)	22 (13 to 34)	0.439 (0.255 to 0.721)	-0.82<br>(-0.90 to -0.73)
Bermuda	0 (0 to 1)	0.771 (0.687 to 0.868)	1 (0 to 1)	0.416 (0.351 to 0.504)	-2.28<br>(-2.99 to -1.56)
Bhutan	1 (0 to 1)	0.323 (0.168 to 0.537)	2 (1 to 3)	0.320 (0.186 to 0.480)	0<br>(-0.11 to 0.11)
Bolivia (Plurinational State of)	14 (6 to 22)	0.415 (0.178 to 0.640)	27 (12 to 40)	0.302 (0.138 to 0.455)	-1.01<br>(-1.10 to -0.92)
Bosnia and Herzegovina	48 (33 to 68)	1.307 (0.889 to 1.871)	84 (57 to 121)	1.322 (0.897 to 1.902)	0.04

					(-0.34 to 0.43)
Botswana	3 (2 to 5)	0.599 (0.374 to 0.860)	5 (3 to 7)	0.348 (0.226 to 0.498)	-1.87 (-2.99 to -0.74)
Brazil	775 (746 to 799)	0.835 (0.793 to 0.866)	1670 (1536 to 1765)	0.677 (0.621 to 0.717)	-0.57 (-1.13 to -0.02)
Brunei Darussalam	1 (1 to 1)	1.209 (0.705 to 1.753)	2 (1 to 3)	1.057 (0.563 to 1.494)	-0.57 (-0.99 to -0.16)
Bulgaria	31 (27 to 35)	0.298 (0.262 to 0.336)	87 (73 to 103)	0.610 (0.514 to 0.722)	2.32 (0.97 to 3.69)
Burkina Faso	30 (16 to 45)	0.766 (0.429 to 1.188)	62 (37 to 102)	0.734 (0.440 to 1.266)	-0.13 (-0.28 to 0.02)
Burundi	23 (12 to 38)	0.945 (0.508 to 1.534)	24 (14 to 38)	0.479 (0.268 to 0.864)	-2.20 (-2.33 to -2.06)
Cabo Verde	1 (1 to 2)	0.490 (0.267 to 0.979)	2 (1 to 3)	0.487 (0.304 to 0.741)	-0.03 (-0.34 to 0.29)
Cambodia	3 (1 to 11)	0.081 (0.035 to 0.273)	6 (3 to 23)	0.063 (0.027 to 0.244)	-0.76 (-0.86 to -0.65)
Cameroon	35 (18 to 53)	0.852 (0.448 to 1.390)	80 (47 to 127)	0.667 (0.397 to 1.097)	-0.80 (-0.91 to -0.7)
Canada	277 (253 to 298)	0.864 (0.787 to 0.926)	326 (275 to 363)	0.399 (0.342 to 0.441)	-2.46 (-3.58 to -1.32)
Central African Republic	11 (5 to 16)	1.042 (0.530 to 1.635)	16 (9 to 25)	0.837 (0.456 to 1.395)	-0.69 (-0.84 to -0.54)
Chad	17 (9 to 27)	0.619 (0.348 to 1.056)	35 (21 to 53)	0.622 (0.376 to 1.014)	0.05 (-0.02 to 0.13)
Chile	57 (53 to 60)	0.608 (0.565 to 0.644)	105 (94 to 113)	0.406 (0.362 to 0.436)	-1.21

					(-2.47 to 0.08)
China	800 (397 to 1054)	0.122 (0.059 to 0.165)	927 (637 to 1248)	0.051 (0.035 to 0.069)	-2.80<br>(-3.04 to -2.55)
Colombia	167 (159 to 175)	0.957 (0.906 to 1.002)	537 (448 to 628)	0.971 (0.813 to 1.138)	-0.17<br>(-0.96 to 0.62)
Comoros	2 (1 to 2)	0.769 (0.370 to 1.302)	2 (1 to 4)	0.497 (0.280 to 0.850)	-1.41<br>(-1.73 to -1.09)
Congo	12 (6 to 17)	1.203 (0.616 to 1.737)	20 (12 to 28)	0.824 (0.502 to 1.203)	-1.20<br>(-1.47 to -0.93)
Cook Islands	0 (0 to 0)	0.046 (0.025 to 0.078)	0 (0 to 0)	0.017 (0.007 to 0.049)	-3.15<br>(-3.26 to -3.03)
Costa Rica	4 (3 to 4)	0.205 (0.184 to 0.227)	18 (16 to 20)	0.326 (0.285 to 0.369)	1.32<br>(0.54 to 2.10)
Croatia	28 (16 to 41)	1.253 (1.145 to 1.389)	63 (35 to 101)	1.080 (0.928 to 1.237)	-0.48<br>(-2.08 to 1.15)
Cuba	69 (63 to 76)	0.507 (0.466 to 0.565)	105 (90 to 120)	0.544 (0.474 to 0.621)	0.22<br>(-0.33 to 0.77)
Cyprus	52 (48 to 58)	3.019 (1.575 to 4.927)	102 (89 to 117)	1.076 (0.647 to 1.687)	-3.16<br>(-4.01 to -2.31)
Czechia	16 (8 to 24)	0.712 (0.668 to 0.758)	18 (11 to 27)	1.034 (0.890 to 1.154)	1.10<br>(0.07 to 2.14)
Côte d'Ivoire	97 (91 to 103)	0.768 (0.452 to 1.230)	236 (203 to 263)	0.597 (0.345 to 0.991)	-0.80<br>(-0.96 to -0.64)
Democratic People's Republic of Korea	21 (12 to 54)	0.155 (0.085 to 0.412)	28 (12 to 94)	0.096 (0.041 to 0.329)	-1.56<br>(-1.63 to -1.48)
Democratic Republic of the Congo	105 (54 to 161)	0.760 (0.407 to 1.249)	224 (127 to 363)	0.699 (0.386 to 1.203)	-0.28

					(-0.36 to -0.19)
Denmark	99 (89 to 109)	1.158 (1.057 to 1.262)	101 (86 to 112)	0.749 (0.646 to 0.830)	-1.32<br>(-1.77 to -0.87)
Djibouti	1 (1 to 1)	0.754 (0.423 to 1.150)	3 (2 to 5)	0.538 (0.320 to 0.878)	-1.06<br>(-1.23 to -0.90)
Dominica	0 (0 to 0)	0.301 (0.210 to 0.398)	0 (0 to 0)	0.313 (0.191 to 0.440)	0.12<br>(-0.01 to 0.25)
Dominican Republic	11 (7 to 15)	0.265 (0.168 to 0.346)	25 (15 to 33)	0.237 (0.143 to 0.318)	-0.29<br>(-0.85 to 0.26)
Ecuador	33 (30 to 36)	0.559 (0.505 to 0.624)	60 (48 to 74)	0.372 (0.300 to 0.458)	-0.79<br>(-2.29 to 0.73)
Egypt	305 (205 to 405)	1.049 (0.735 to 1.473)	435 (307 to 565)	0.673 (0.497 to 0.918)	-1.37<br>(-1.69 to -1.05)
El Salvador	1 (1 to 2)	0.037 (0.021 to 0.055)	3 (1 to 4)	0.04 (0.017 to 0.056)	0.25<br>(-0.44 to 0.95)
Equatorial Guinea	2 (1 to 3)	0.985 (0.486 to 1.566)	3 (2 to 5)	0.631 (0.366 to 1.040)	-1.38<br>(-1.76 to -1.01)
Eritrea	11 (6 to 17)	0.981 (0.527 to 1.518)	19 (12 to 27)	0.712 (0.449 to 1.149)	-1.04<br>(-1.17 to -0.91)
Estonia	5 (4 to 6)	0.264 (0.221 to 0.316)	19 (15 to 22)	0.627 (0.532 to 0.736)	2.91<br>(1.29 to 4.55)
Eswatini	2 (1 to 3)	0.554 (0.320 to 0.796)	3 (2 to 5)	0.526 (0.304 to 0.779)	-0.17<br>(-0.35 to 0.01)
Ethiopia	138 (59 to 214)	0.676 (0.320 to 1.055)	161 (109 to 249)	0.353 (0.229 to 0.563)	-2.08<br>(-2.21 to -1.95)
Fiji	0 (0 to 0)	0.058 (0.037 to 0.078)	0 (0 to 1)	0.055 (0.031 to 0.080)	-0.18

					(-0.50 to 0.15)
Finland	108 (89 to 128)	1.503 (1.253 to 1.785)	157 (128 to 175)	1.015 (0.851 to 1.117)	-1.22<br>(-1.84 to -0.60)
France	706 (627 to 773)	0.802 (0.722 to 0.868)	582 (483 to 650)	0.303 (0.259 to 0.336)	-3.01<br>(-3.52 to -2.50)
Gabon	6 (3 to 8)	1.050 (0.587 to 1.560)	7 (4 to 10)	0.731 (0.421 to 1.069)	-1.14<br>(-1.34 to -0.93)
Gambia	2 (1 to 4)	0.708 (0.379 to 1.223)	6 (4 to 10)	0.666 (0.396 to 1.033)	-0.20<br>(-0.81 to 0.41)
Georgia	4 (3 to 5)	0.065 (0.053 to 0.080)	79 (68 to 91)	1.308 (1.133 to 1.509)	10.11<br>(7.34 to 12.96)
Germany	2302 (1988 to 2627)	1.747 (1.513 to 2.000)	3010 (2440 to 3377)	1.219 (1.015 to 1.356)	-1.14<br>(-1.58 to -0.70)
Ghana	57 (32 to 81)	0.958 (0.549 to 1.401)	101 (58 to 153)	0.658 (0.373 to 1.021)	-1.20<br>(-1.27 to -1.12)
Greece	121 (109 to 130)	0.843 (0.759 to 0.906)	215 (179 to 240)	0.674 (0.576 to 0.743)	-0.71<br>(-1.23 to -0.19)
Greenland	1 (0 to 1)	1.946 (0.955 to 2.959)	0 (0 to 1)	0.881 (0.502 to 1.429)	-2.48<br>(-2.70 to -2.25)
Grenada	1 (1 to 1)	0.989 (0.795 to 1.166)	1 (1 to 1)	1.113 (0.957 to 1.264)	0.52<br>(0.13 to 0.91)
Guam	0 (0 to 0)	0.031 (0.011 to 0.047)	0 (0 to 0)	0.008 (0.004 to 0.020)	-4.12<br>(-5.20 to -3.02)
Guatemala	5 (4 to 7)	0.167 (0.129 to 0.210)	17 (15 to 20)	0.160 (0.137 to 0.186)	-0.50<br>(-1.02 to 0.03)
Guinea	21 (11 to 34)	0.683 (0.361 to 1.117)	34 (20 to 53)	0.605 (0.352 to 1.002)	-0.38

					(-0.51 to -0.25)
Guinea-Bissau	4 (2 to 6)	1.061 (0.580 to 1.644)	6 (3 to 9)	0.843 (0.491 to 1.261)	-0.74<br>(-0.81 to -0.68)
Guyana	4 (3 to 4)	0.894 (0.774 to 1.023)	5 (4 to 7)	0.801 (0.619 to 1.036)	0<br>(-0.97 to 0.98)
Haiti	30 (15 to 49)	0.850 (0.449 to 1.308)	57 (32 to 88)	0.694 (0.397 to 1.076)	-0.59<br>(-0.7 to -0.48)
Honduras	6 (3 to 9)	0.310 (0.160 to 0.471)	23 (13 to 33)	0.397 (0.213 to 0.555)	0.83<br>(0.41 to 1.26)
Hungary	241 (223 to 258)	1.709 (1.584 to 1.811)	328 (291 to 362)	1.599 (1.422 to 1.765)	-0.24<br>(-0.71 to 0.23)
Iceland	2 (2 to 3)	0.753 (0.662 to 0.824)	3 (3 to 4)	0.475 (0.402 to 0.532)	-1.54<br>(-2.00 to -1.08)
India	1439 (802 to 2106)	0.338 (0.190 to 0.491)	3867 (2583 to 5084)	0.355 (0.235 to 0.471)	0.20<br>(-0.3 to 0.69)
Indonesia	74 (36 to 248)	0.085 (0.040 to 0.303)	146 (58 to 545)	0.081 (0.032 to 0.318)	-0.13<br>(-0.23 to -0.03)
Iran (Islamic Republic of)	189 (130 to 240)	0.768 (0.491 to 0.965)	390 (263 to 475)	0.530 (0.341 to 0.650)	-1.21<br>(-1.40 to -1.03)
Iraq	23 (11 to 53)	0.279 (0.137 to 0.620)	56 (24 to 139)	0.248 (0.116 to 0.583)	-0.34<br>(-0.49 to -0.19)
Ireland	41 (37 to 44)	1.036 (0.940 to 1.122)	37 (31 to 42)	0.438 (0.367 to 0.494)	-2.69<br>(-3.29 to -2.10)
Israel	80 (71 to 89)	1.718 (1.513 to 1.896)	105 (89 to 118)	0.761 (0.650 to 0.852)	-2.55<br>(-3.06 to -2.05)
Italy	1102 (1018 to 1149)	1.243 (1.148 to 1.297)	1354 (1097 to 1492)	0.722 (0.606 to 0.784)	-1.65

					(-2.20 to -1.11)
Jamaica	4 (4 to 5)	0.229 (0.201 to 0.257)	9 (7 to 12)	0.294 (0.230 to 0.371)	1.80<br>(0.47 to 3.14)
Japan	2499 (2239 to 2634)	1.630 (1.441 to 1.729)	3654 (2716 to 4205)	0.590 (0.462 to 0.663)	-3.34<br>(-3.67 to -3.01)
Jordan	6 (4 to 8)	0.422 (0.288 to 0.577)	15 (10 to 20)	0.211 (0.134 to 0.283)	-2.18<br>(-2.71 to -1.65)
Kazakhstan	7 (6 to 9)	0.058 (0.045 to 0.074)	45 (37 to 55)	0.279 (0.232 to 0.335)	5.15<br>(2.83 to 7.52)
Kenya	36 (21 to 51)	0.428 (0.259 to 0.628)	93 (64 to 135)	0.451 (0.292 to 0.695)	0.17<br>(0.12 to 0.21)
Kiribati	0 (0 to 0)	0.238 (0.158 to 0.437)	0 (0 to 0)	0.241 (0.149 to 0.460)	0.03<br>(-0.07 to 0.13)
Kuwait	2 (2 to 2)	0.224 (0.199 to 0.247)	5 (4 to 6)	0.179 (0.147 to 0.219)	-0.54<br>(-3.03 to 2.03)
Kyrgyzstan	2 (2 to 3)	0.073 (0.064 to 0.084)	6 (5 to 7)	0.132 (0.105 to 0.159)	1.26<br>(-1.60 to 4.20)
Lao People's Democratic Republic	2 (1 to 6)	0.106 (0.047 to 0.349)	3 (1 to 11)	0.073 (0.030 to 0.280)	-1.23<br>(-1.30 to -1.15)
Latvia	4 (3 to 4)	0.110 (0.095 to 0.126)	10 (9 to 12)	0.258 (0.224 to 0.294)	2.98<br>(1.46 to 4.52)
Lebanon	28 (15 to 47)	1.322 (0.704 to 2.196)	21 (14 to 31)	0.346 (0.224 to 0.500)	-4.27<br>(-4.52 to -4.03)
Lesotho	4 (2 to 5)	0.429 (0.270 to 0.630)	7 (4 to 10)	0.599 (0.343 to 0.868)	1.16<br>(0.77 to 1.56)
Liberia	8 (4 to 12)	0.702 (0.356 to 1.163)	11 (6 to 19)	0.537 (0.286 to 0.939)	-0.87

					(-1.05 to -0.69)
Libya	15 (9 to 26)	0.717 (0.449 to 1.245)	47 (22 to 105)	0.864 (0.395 to 1.954)	0.59<br>(0.28 to 0.90)
Lithuania	4 (3 to 5)	0.086 (0.071 to 0.105)	14 (12 to 17)	0.249 (0.210 to 0.295)	3.62<br>(2.23 to 5.04)
Luxembourg	9 (8 to 10)	1.736 (1.576 to 1.896)	10 (8 to 11)	0.795 (0.670 to 0.898)	-2.47<br>(-3.08 to -1.86)
Madagascar	64 (35 to 89)	1.159 (0.680 to 1.702)	116 (73 to 164)	0.940 (0.581 to 1.466)	-0.70<br>(-0.87 to -0.52)
Malawi	20 (9 to 32)	0.516 (0.263 to 0.896)	37 (23 to 60)	0.485 (0.279 to 0.847)	-0.20<br>(-0.36 to -0.03)
Malaysia	14 (9 to 19)	0.149 (0.096 to 0.206)	32 (21 to 43)	0.124 (0.080 to 0.165)	-0.73<br>(-1.03 to -0.42)
Maldives	0 (0 to 0)	0.288 (0.173 to 0.483)	1 (0 to 1)	0.210 (0.117 to 0.294)	-1.14<br>(-1.43 to -0.85)
Mali	24 (11 to 37)	0.644 (0.328 to 1.052)	42 (23 to 63)	0.472 (0.272 to 0.772)	-0.97<br>(-1.05 to -0.89)
Malta	4 (3 to 4)	0.921 (0.830 to 1.004)	6 (5 to 7)	0.511 (0.436 to 0.589)	-2.06<br>(-2.88 to -1.23)
Marshall Islands	0 (0 to 0)	0.089 (0.052 to 0.139)	0 (0 to 0)	0.063 (0.033 to 0.128)	-1.11<br>(-1.19 to -1.03)
Mauritania	8 (5 to 12)	0.882 (0.479 to 1.332)	11 (7 to 18)	0.570 (0.330 to 0.908)	-1.41<br>(-1.53 to -1.29)
Mauritius	4 (4 to 4)	0.554 (0.515 to 0.597)	8 (8 to 9)	0.492 (0.450 to 0.523)	0.79<br>(-8.38 to 10.88)
Mexico	133 (129 to 135)	0.332 (0.320 to 0.341)	239 (213 to 266)	0.197 (0.175 to 0.219)	-2.08

					(-4.19 to 0.08)
Micronesia (Federated States of)	0 (0 to 0)	0.096 (0.056 to 0.147)	0 (0 to 0)	0.05 (0.029 to 0.095)	-2.10<br>(-2.17 to -2.04)
Monaco	0 (0 to 1)	0.528 (0.326 to 0.776)	1 (0 to 1)	0.441 (0.261 to 0.659)	-0.58<br>(-0.74 to -0.43)
Mongolia	5 (3 to 7)	0.451 (0.303 to 0.69)	9 (7 to 12)	0.407 (0.295 to 0.542)	-0.34<br>(-0.80 to 0.13)
Montenegro	3 (2 to 3)	0.441 (0.281 to 0.57)	5 (3 to 7)	0.565 (0.338 to 0.763)	0.92<br>(0.30 to 1.54)
Morocco	141 (87 to 240)	0.969 (0.588 to 1.598)	311 (197 to 557)	0.956 (0.611 to 1.688)	-0.03<br>(-0.10 to 0.04)
Mozambique	37 (18 to 61)	0.667 (0.333 to 1.188)	79 (41 to 138)	0.731 (0.371 to 1.408)	0.33<br>(0.15 to 0.50)
Myanmar	21 (10 to 66)	0.101 (0.048 to 0.338)	31 (14 to 112)	0.073 (0.032 to 0.274)	-1.02<br>(-1.12 to -0.92)
Namibia	4 (2 to 5)	0.566 (0.356 to 0.758)	6 (4 to 10)	0.472 (0.315 to 0.713)	-0.50<br>(-0.8 to -0.19)
Nauru	0 (0 to 0)	0.12 (0.074 to 0.181)	0 (0 to 0)	0.074 (0.038 to 0.157)	-1.60<br>(-1.85 to -1.36)
Nepal	27 (15 to 42)	0.322 (0.176 to 0.5)	67 (39 to 101)	0.326 (0.19 to 0.5)	0.05<br>(-0.10 to 0.21)
Netherlands	979 (876 to 1054)	4.748 (4.235 to 5.117)	745 (617 to 835)	1.855 (1.554 to 2.065)	-3.02<br>(-3.6 to -2.43)
New Zealand	55 (49 to 61)	1.467 (1.302 to 1.609)	83 (71 to 94)	0.908 (0.784 to 1.018)	-1.49<br>(-2.70 to -0.26)
Nicaragua	1 (1 to 2)	0.088 (0.062 to 0.126)	5 (3 to 7)	0.104 (0.053 to 0.14)	0.44

					(0.07 to 0.82)
Niger	16 (8 to 27)	0.660 (0.338 to 1.171)	38 (20 to 69)	0.516 (0.276 to 0.950)	-0.78<br>(-0.87 to -0.69)
Nigeria	322 (179 to 491)	0.802 (0.446 to 1.277)	419 (236 to 685)	0.506 (0.291 to 0.824)	-1.49<br>(-1.58 to -1.39)
Niue	0 (0 to 0)	0.066 (0.042 to 0.110)	0 (0 to 0)	0.055 (0.029 to 0.129)	-0.46<br>(-1.19 to 0.28)
North Macedonia	8 (6 to 11)	0.501 (0.362 to 0.673)	18 (9 to 25)	0.672 (0.327 to 0.960)	0.95<br>(0.50 to 1.41)
Northern Mariana Islands	0 (0 to 0)	0.012 (0.003 to 0.023)	0 (0 to 0)	0.003 (0.001 to 0.008)	-5.70<br>(-8.15 to -3.18)
Norway	134 (120 to 143)	1.714 (1.541 to 1.829)	75 (62 to 83)	0.601 (0.507 to 0.663)	-3.51<br>(-4.86 to -2.13)
Oman	4 (2 to 5)	0.508 (0.329 to 0.729)	6 (4 to 7)	0.297 (0.190 to 0.378)	-1.70<br>(-2.07 to -1.32)
Pakistan	174 (103 to 246)	0.328 (0.192 to 0.471)	458 (278 to 651)	0.401 (0.237 to 0.570)	0.68<br>(0.59 to 0.77)
Palau	0 (0 to 0)	0.07 (0.044 to 0.108)	0 (0 to 0)	0.059 (0.031 to 0.122)	-0.60<br>(-0.83 to -0.37)
Palestine	3 (2 to 4)	0.337 (0.199 to 0.468)	5 (4 to 7)	0.223 (0.151 to 0.299)	-1.38<br>(-1.55 to -1.20)
Panama	3 (3 to 4)	0.225 (0.199 to 0.249)	19 (15 to 23)	0.423 (0.334 to 0.511)	2.09<br>(1.54 to 2.64)
Papua New Guinea	2 (1 to 5)	0.065 (0.030 to 0.129)	4 (2 to 8)	0.041 (0.023 to 0.083)	-1.46<br>(-1.77 to -1.14)
Paraguay	11 (7 to 15)	0.467 (0.301 to 0.635)	32 (20 to 45)	0.555 (0.330 to 0.774)	0.62

					(0.23 to 1.01)
Peru	15 (9 to 22)	0.115 (0.069 to 0.163)	34 (18 to 47)	0.098 (0.053 to 0.138)	-0.43<br>(-1.99 to 1.14)
Philippines	13 (6 to 16)	0.053 (0.024 to 0.068)	39 (15 to 53)	0.052 (0.020 to 0.072)	-0.03<br>(-0.50 to 0.43)
Poland	162 (156 to 167)	0.376 (0.361 to 0.387)	749 (669 to 817)	1.001 (0.899 to 1.091)	3.07<br>(2.39 to 3.74)
Portugal	112 (104 to 119)	0.894 (0.831 to 0.951)	178 (146 to 200)	0.584 (0.493 to 0.646)	-1.39<br>(-2.24 to -0.53)
Puerto Rico	13 (12 to 14)	0.374 (0.349 to 0.398)	17 (14 to 20)	0.260 (0.214 to 0.307)	-1.07<br>(-2.69 to 0.58)
Qatar	1 (1 to 1)	0.874 (0.619 to 1.264)	3 (2 to 5)	0.380 (0.240 to 0.535)	-2.82<br>(-3.56 to -2.08)
Republic of Korea	107 (63 to 148)	0.554 (0.331 to 0.840)	232 (115 to 326)	0.261 (0.129 to 0.367)	-2.53<br>(-2.91 to -2.15)
Republic of Moldova	4 (3 to 4)	0.090 (0.080 to 0.101)	13 (11 to 15)	0.220 (0.196 to 0.251)	2.93<br>(1.51 to 4.38)
Romania	189 (164 to 212)	0.713 (0.618 to 0.796)	196 (167 to 227)	0.509 (0.440 to 0.589)	-1.29<br>(-1.92 to -0.67)
Russian Federation	120 (115 to 123)	0.072 (0.069 to 0.075)	616 (563 to 667)	0.267 (0.245 to 0.289)	4.42<br>(3.50 to 5.35)
Rwanda	35 (18 to 51)	1.167 (0.630 to 1.771)	31 (20 to 50)	0.540 (0.315 to 0.908)	-2.47<br>(-2.57 to -2.36)
Saint Kitts and Nevis	0 (0 to 0)	0.643 (0.557 to 0.769)	0 (0 to 0)	0.529 (0.444 to 0.612)	-0.23<br>(-1.51 to 1.06)
Saint Lucia	0 (0 to 0)	0.296 (0.261 to 0.337)	0 (0 to 1)	0.206 (0.169 to 0.250)	-1.15

					(-2.21 to -0.07)
Saint Vincent and the Grenadines	0 (0 to 0)	0.297 (0.257 to 0.334)	1 (1 to 1)	0.601 (0.524 to 0.683)	2.18<br>(0.94 to 3.43)
Samoa	0 (0 to 0)	0.057 (0.036 to 0.090)	0 (0 to 0)	0.037 (0.021 to 0.075)	-1.33<br>(-1.47 to -1.20)
San Marino	0 (0 to 0)	0.465 (0.280 to 0.631)	0 (0 to 0)	0.242 (0.136 to 0.377)	-2.40<br>(-2.68 to -2.12)
Sao Tome and Principe	0 (0 to 1)	0.500 (0.254 to 0.922)	1 (0 to 1)	0.503 (0.316 to 0.813)	0.01<br>(-0.22 to 0.23)
Saudi Arabia	48 (33 to 65)	0.729 (0.497 to 1.015)	136 (88 to 188)	0.582 (0.396 to 0.768)	-0.74<br>(-0.92 to -0.55)
Senegal	22 (12 to 34)	0.712 (0.400 to 1.153)	45 (28 to 73)	0.619 (0.379 to 1.043)	-0.49<br>(-0.79 to -0.19)
Serbia	269 (216 to 362)	2.980 (2.325 to 4.093)	445 (289 to 573)	2.578 (1.683 to 3.313)	-0.37<br>(-0.73 to -0.01)
Seychelles	0 (0 to 0)	0.337 (0.219 to 0.608)	0 (0 to 0)	0.215 (0.119 to 0.460)	-1.49<br>(-1.92 to -1.06)
Sierra Leone	13 (7 to 20)	0.650 (0.366 to 1.055)	21 (12 to 34)	0.552 (0.320 to 0.918)	-0.52<br>(-0.59 to -0.45)
Singapore	15 (14 to 16)	0.820 (0.753 to 0.875)	20 (17 to 22)	0.244 (0.210 to 0.265)	-3.91<br>(-4.46 to -3.35)
Slovakia	30 (23 to 43)	0.514 (0.395 to 0.722)	46 (24 to 62)	0.480 (0.255 to 0.651)	-0.24<br>(-0.44 to -0.04)
Slovenia	39 (35 to 43)	1.574 (1.411 to 1.733)	68 (55 to 81)	1.302 (1.064 to 1.555)	-0.59<br>(-1.21 to 0.04)
Solomon Islands	0 (0 to 0)	0.041 (0.021 to 0.073)	0 (0 to 0)	0.027 (0.015 to 0.051)	-1.38

					(-1.59 to -1.16)
Somalia	18 (7 to 31)	0.708 (0.311 to 1.249)	30 (14 to 51)	0.474 (0.215 to 0.952)	-1.24<br>(-1.31 to -1.18)
South Africa	112 (68 to 135)	0.439 (0.259 to 0.533)	166 (120 to 219)	0.360 (0.255 to 0.481)	-0.72<br>(-1.16 to -0.29)
South Sudan	18 (9 to 27)	0.686 (0.352 to 1.088)	18 (9 to 29)	0.465 (0.247 to 0.813)	-1.24<br>(-1.39 to -1.09)
Spain	691 (620 to 743)	1.305 (1.173 to 1.401)	1121 (901 to 1264)	0.859 (0.710 to 0.961)	-1.31<br>(-1.74 to -0.88)
Sri Lanka	10 (4 to 14)	0.100 (0.042 to 0.138)	8 (5 to 13)	0.034 (0.020 to 0.054)	-3.28<br>(-3.69 to -2.86)
Sudan	108 (61 to 194)	1.117 (0.672 to 1.914)	176 (99 to 342)	0.843 (0.496 to 1.545)	-0.91<br>(-0.96 to -0.85)
Suriname	2 (1 to 2)	0.683 (0.454 to 0.846)	3 (2 to 5)	0.547 (0.343 to 0.731)	-0.62<br>(-1.06 to -0.18)
Sweden	156 (141 to 169)	0.922 (0.829 to 0.991)	144 (117 to 162)	0.51 (0.424 to 0.571)	-2.06<br>(-2.51 to -1.61)
Switzerland	145 (127 to 164)	1.288 (1.124 to 1.444)	144 (115 to 164)	0.617 (0.505 to 0.695)	-2.32<br>(-3.1 to -1.53)
Syrian Arab Republic	102 (66 to 179)	1.948 (1.258 to 3.408)	146 (89 to 290)	1.303 (0.841 to 2.504)	-1.24<br>(-1.59 to -0.90)
Taiwan (Province of China)	136 (128 to 144)	0.977 (0.902 to 1.041)	146 (127 to 160)	0.342 (0.300 to 0.374)	-3.05<br>(-5.00 to -1.05)
Tajikistan	0 (0 to 0)	0.008 (0.004 to 0.015)	0 (0 to 1)	0.006 (0.003 to 0.010)	-0.90<br>(-1.31 to -0.50)
Thailand	49 (34 to 92)	0.149 (0.098 to 0.296)	242 (121 to 368)	0.234 (0.119 to 0.353)	1.34

					(0.55 to 2.13)
Timor-Leste	0 (0 to 1)	0.077 (0.033 to 0.269)	0 (0 to 2)	0.066 (0.025 to 0.255)	-0.51<br>(-0.71 to -0.31)
Togo	9 (5 to 13)	0.744 (0.411 to 1.162)	23 (13 to 35)	0.652 (0.370 to 1.113)	-0.41<br>(-0.54 to -0.28)
Tokelau	0 (0 to 0)	0.082 (0.050 to 0.141)	0 (0 to 0)	0.061 (0.033 to 0.134)	-0.73<br>(-1.54 to 0.09)
Tonga	0 (0 to 0)	0.032 (0.020 to 0.051)	0 (0 to 0)	0.019 (0.009 to 0.044)	-1.62<br>(-1.7 to -1.53)
Trinidad and Tobago	4 (4 to 5)	0.499 (0.454 to 0.547)	8 (6 to 10)	0.421 (0.324 to 0.532)	-0.34<br>(-0.66 to -0.02)
Tunisia	40 (26 to 70)	0.861 (0.540 to 1.516)	86 (49 to 172)	0.691 (0.392 to 1.370)	-0.72<br>(-0.81 to -0.64)
Turkey	647 (406 to 864)	1.873 (1.160 to 2.494)	859 (629 to 1189)	0.953 (0.696 to 1.337)	-2.13<br>(-2.45 to -1.81)
Turkmenistan	0 (0 to 1)	0.023 (0.019 to 0.027)	5 (4 to 7)	0.133 (0.101 to 0.177)	5.60<br>(2.72 to 8.57)
Tuvalu	0 (0 to 0)	0.099 (0.056 to 0.162)	0 (0 to 0)	0.045 (0.026 to 0.094)	-2.50<br>(-2.59 to -2.40)
Uganda	36 (19 to 61)	0.573 (0.306 to 1.029)	67 (41 to 110)	0.446 (0.266 to 0.782)	-0.81<br>(-0.93 to -0.68)
Ukraine	16 (13 to 19)	0.025 (0.021 to 0.030)	81 (61 to 103)	0.113 (0.085 to 0.144)	4.85<br>(2.35 to 7.40)
United Arab Emirates	2 (1 to 4)	0.480 (0.260 to 0.714)	7 (5 to 12)	0.240 (0.143 to 0.361)	-2.14<br>(-3.99 to -0.25)
United Kingdom	712 (663 to 738)	0.764 (0.710 to 0.792)	872 (751 to 932)	0.573 (0.500 to 0.608)	-0.93

					(-1.53 to -0.33)
United Republic of Tanzania	73 (41 to 116)	0.712 (0.392 to 1.198)	128 (75 to 220)	0.520 (0.299 to 0.949)	-1.01<br>(-1.08 to -0.93)
United States of America	3373 (3030 to 3557)	1.014 (0.914 to 1.069)	3877 (3267 to 4196)	0.614 (0.527 to 0.660)	-1.65<br>(-2.21 to -1.09)
United States Virgin Islands	1 (1 to 1)	1.104 (0.760 to 1.421)	1 (1 to 1)	0.516 (0.353 to 0.725)	-2.41<br>(-2.58 to -2.23)
Uruguay	28 (25 to 31)	0.719 (0.642 to 0.790)	37 (32 to 41)	0.612 (0.544 to 0.674)	-0.55<br>(-1.60 to 0.51)
Uzbekistan	12 (9 to 15)	0.099 (0.071 to 0.133)	55 (45 to 65)	0.224 (0.184 to 0.267)	2.10<br>(0.26 to 3.98)
Vanuatu	0 (0 to 0)	0.060 (0.035 to 0.099)	0 (0 to 0)	0.040 (0.021 to 0.086)	-1.34<br>(-1.64 to -1.04)
Venezuela (Bolivarian Republic of)	40 (35 to 46)	0.408 (0.356 to 0.466)	171 (131 to 219)	0.591 (0.453 to 0.754)	1.14<br>(-0.02 to 2.32)
Viet Nam	28 (12 to 108)	0.077 (0.031 to 0.313)	57 (21 to 244)	0.070 (0.024 to 0.310)	-0.32<br>(-0.42 to -0.21)
Yemen	58 (33 to 101)	1.220 (0.710 to 2.049)	150 (86 to 249)	1.072 (0.643 to 1.820)	-0.39<br>(-0.54 to -0.24)
Zambia	24 (12 to 34)	0.803 (0.440 to 1.219)	50 (33 to 76)	0.726 (0.461 to 1.117)	-0.31<br>(-0.49 to -0.12)
Zimbabwe	17 (10 to 27)	0.424 (0.234 to 0.615)	48 (29 to 68)	0.601 (0.358 to 0.851)	1.19<br>(0.51 to 1.88)

Abbreviations: AAPC, annual percentage change; CI, confidence interval; DMVD, degenerative mitral valve disease; UI, uncertain interval.

Supplementary Table 3: Age-standardized DALYs rates and average annual percentage change for DMVD at national level, 1990-2021

	1990		2021		1990 – 2021
**Nation**	**Cases (95% UI)**	**Age-standardized DALYs per 100 000 population (95% UI)**	**Cases (95% UI)**	**Age- standardized DALYs per 100 000 population (95% UI)**	**AAPC % (95% CI)**
Afghanistan	2598 (1094 to 4848)	36.060 (16.063 to 66.523)	4562 (1915 to 8509)	31.181 (14.607 to 54.722)	-0.45 (-0.54 to -0.37)
Albania	425 (324 to 542)	21.180 (16.229 to 27.288)	739 (505 to 1058)	17.325 (11.827 to 24.895)	-0.76 (-1.13 to -0.38)
Algeria	3934 (2512 to 6756)	27.472 (18.058 to 44.767)	7468 (4618 to 13596)	19.956 (12.688 to 35.778)	-1.02 (-1.09 to -0.96)
American Samoa	5 (3 to 7)	12.987 (8.394 to 17.194)	4 (3 to 5)	7.904 (5.783 to 10.994)	-1.55 (-1.87 to -1.24)
Andorra	7 (5 to 10)	13.100 (8.595 to 19.525)	12 (8 to 19)	7.348 (4.901 to 11.665)	-1.90 (-2.18 to -1.62)
Angola	1115 (570 to 1715)	22.908 (12.787 to 33.577)	2760 (1671 to 4222)	18.791 (11.456 to 29.460)	-0.60 (-1.12 to -0.09)
Antigua and Barbuda	10 (9 to 11)	18.724 (17.209 to 20.403)	14 (13 to 15)	13.102 (12.117 to 14.176)	-0.94 (-2.23 to 0.37)
Argentina	4542 (3943 to 5222)	14.261 (12.378 to 16.373)	4874 (4270 to 5757)	8.704 (7.690 to 10.197)	-1.49 (-2.15 to -0.82)
Armenia	188 (129 to 274)	7.617 (5.180 to 11.189)	450 (336 to 608)	10.588 (7.966 to 14.261)	0.94 (0.46 to 1.42)
Australia	4077 (3755 to 4496)	21.229 (19.556 to 23.293)	4954 (4274 to 5751)	10.528 (9.230 to 12.088)	-2.09 (-2.47 to -1.70)
Austria	3276 (2863 to 3936)	27.481 (24.272 to 32.485)	3984 (3357 to 4903)	19.600 (16.677 to 24.064)	-1.03 (-1.95 to -0.10)

Azerbaijan	290 (178 to 468)	6.569 (3.936 to 10.760)	574 (344 to 929)	7.213 (4.259 to 11.716)	0.30<br>(0.16 to 0.44)
Bahamas	24 (21 to 27)	11.782 (10.388 to 13.087)	54 (43 to 67)	12.447 (10.048 to 15.509)	0.06<br>(-0.54 to 0.65)
Bahrain	42 (29 to 54)	17.728 (12.285 to 23.052)	94 (61 to 124)	8.860 (5.898 to 11.545)	-2.27<br>(-2.66 to -1.88)
Bangladesh	5659 (3172 to 8852)	10.051 (5.906 to 14.896)	11609 (7322 to 16793)	8.349 (5.291 to 12.065)	-0.44<br>(-0.75 to -0.13)
Barbados	17 (15 to 19)	6.149 (5.497 to 6.865)	23 (19 to 29)	5.345 (4.276 to 6.741)	-0.25<br>(-1.00 to 0.50)
Belarus	437 (247 to 739)	3.519 (2.001 to 5.884)	601 (386 to 974)	3.769 (2.489 to 6.036)	0.23<br>(0.09 to 0.37)
Belgium	6336 (5769 to 6883)	41.154 (37.528 to 44.391)	3124 (2703 to 3491)	12.549 (11.145 to 13.841)	-3.84<br>(-4.38 to -3.30)
Belize	15 (13 to 19)	12.717 (11.250 to 15.836)	50 (44 to 57)	14.156 (12.521 to 15.942)	0.59<br>(-0.35 to 1.53)
Benin	313 (182 to 443)	13.423 (7.835 to 19.511)	677 (397 to 1024)	10.278 (6.030 to 15.959)	-0.85<br>(-0.96 to -0.73)
Bermuda	15 (14 to 17)	23.135 (20.812 to 25.797)	13 (11 to 16)	11.731 (9.858 to 14.234)	-2.27<br>(-2.98 to -1.55)
Bhutan	25 (12 to 43)	8.275 (4.422 to 13.533)	47 (30 to 73)	7.454 (4.765 to 11.207)	-0.30<br>(-0.4 to -0.21)
Bolivia (Plurinational State of)	517 (231 to 791)	12.377 (5.608 to 18.779)	826 (422 to 1254)	8.239 (4.208 to 12.424)	-1.31<br>(-1.42 to -1.19)
Bosnia and Herzegovina	1326 (957 to 1801)	33.089 (24.140 to 44.556)	1938 (1396 to 2615)	31.618 (22.795 to 43.204)	-0.17<br>(-0.55 to 0.21)

Botswana	134 (89 to 188)	17.001 (11.099 to 23.577)	184 (119 to 268)	9.549 (6.354 to 13.504)	-2.00 (-3.30 to -0.69)
Brazil	26987 (26288 to 27640)	24.489 (23.700 to 25.234)	44784 (42312 to 46807)	17.892 (16.876 to 18.709)	-0.99 (-1.65 to -0.34)
Brunei Darussalam	27 (18 to 36)	26.683 (18.227 to 36.078)	64 (41 to 85)	22.738 (14.672 to 30.222)	-0.57 (-0.77 to -0.37)
Bulgaria	1134 (925 to 1470)	9.943 (8.201 to 12.747)	2229 (1834 to 2692)	16.598 (13.790 to 19.775)	1.81 (1.33 to 2.31)
Burkina Faso	844 (457 to 1265)	17.771 (9.957 to 26.994)	1770 (993 to 2763)	16.355 (9.596 to 26.591)	-0.25 (-0.43 to -0.08)
Burundi	835 (394 to 1386)	26.096 (13.356 to 42.728)	883 (515 to 1346)	12.247 (7.155 to 19.817)	-2.45 (-2.71 to -2.18)
Cabo Verde	32 (19 to 55)	12.769 (7.706 to 22.727)	53 (34 to 77)	10.970 (7.087 to 16.203)	-0.51 (-0.81 to -0.20)
Cambodia	139 (73 to 390)	3.001 (1.661 to 7.660)	278 (150 to 705)	2.509 (1.377 to 6.146)	-0.57 (-0.65 to -0.50)
Cameroon	1083 (536 to 1596)	20.412 (10.839 to 30.770)	2676 (1557 to 4094)	16.067 (9.338 to 25.456)	-0.78 (-0.89 to -0.67)
Canada	7082 (6176 to 8613)	21.727 (19.010 to 26.317)	8417 (6703 to 11058)	11.316 (9.193 to 14.620)	-2.05 (-2.68 to -1.42)
Central African Republic	353 (164 to 545)	25.829 (12.784 to 38.843)	563 (296 to 860)	20.305 (11.223 to 31.454)	-0.77 (-0.95 to -0.60)
Chad	467 (255 to 728)	14.731 (8.278 to 22.926)	1127 (679 to 1718)	14.859 (8.967 to 22.604)	0.03 (-0.13 to 0.20)
Chile	1509 (1389 to 1655)	14.978 (13.716 to 16.556)	2641 (2341 to 3056)	10.400 (9.246 to 11.966)	-1.14 (-2.14 to -0.14)

China	34735 (21437 to 46510)	4.488 (2.893 to 6.113)	53736 (38123 to 79238)	2.791 (2.012 to 4.079)	-1.53 (-1.72 to -1.35)
Colombia	5093 (4865 to 5341)	24.551 (23.364 to 25.790)	12843 (10783 to 14996)	23.364 (19.645 to 27.292)	-0.37 (-1.21 to 0.47)
Comoros	57 (21 to 90)	20.964 (9.111 to 32.970)	72 (41 to 114)	12.542 (7.187 to 20.230)	-1.75 (-2.64 to -0.85)
Congo	364 (177 to 545)	29.418 (14.884 to 42.842)	644 (374 to 896)	19.699 (11.833 to 27.688)	-1.27 (-1.58 to -0.96)
Cook Islands	1 (0 to 1)	3.644 (2.330 to 5.626)	0 (0 to 1)	1.941 (1.101 to 3.649)	-2.00 (-2.09 to -1.91)
Costa Rica	104 (93 to 116)	5.398 (4.838 to 6.049)	457 (398 to 515)	8.342 (7.294 to 9.393)	1.09 (0.40 to 1.79)
Croatia	958 (537 to 1408)	29.741 (26.429 to 34.169)	2110 (1173 to 3244)	25.201 (21.477 to 30.060)	-0.54 (-1.43 to 0.37)
Cuba	1711 (1521 to 1968)	15.783 (14.636 to 17.525)	2284 (1937 to 2766)	15.717 (13.741 to 17.920)	-0.04 (-0.61 to 0.52)
Cyprus	1694 (1571 to 1879)	44.422 (24.963 to 67.607)	2693 (2342 to 3089)	16.294 (10.805 to 24.211)	-3.20 (-3.89 to -2.50)
Czechia	281 (157 to 408)	21.841 (19.486 to 25.316)	302 (200 to 440)	24.844 (21.452 to 29.448)	0.35 (-0.31 to 1.02)
Côte d'Ivoire	2942 (2607 to 3431)	18.138 (10.488 to 26.919)	5372 (4608 to 6451)	14.274 (8.227 to 22.901)	-0.76 (-0.97 to -0.55)
Democratic People's Republic of Korea	795 (483 to 1682)	5.242 (3.247 to 10.703)	1100 (603 to 2585)	3.615 (1.976 to 8.522)	-1.20 (-1.25 to -1.14)
Democratic Republic of the Congo	3408 (1728 to 5081)	18.407 (9.754 to 28.447)	7031 (3919 to 11028)	16.120 (9.137 to 26.215)	-0.45 (-0.56 to -0.33)

Denmark	1918 (1758 to 2100)	23.791 (21.971 to 25.814)	1717 (1528 to 1916)	14.030 (12.692 to 15.469)	-1.62 (-2.04 to -1.19)
Djibouti	39 (21 to 58)	18.873 (10.907 to 26.595)	102 (61 to 161)	12.785 (7.703 to 20.343)	-1.25 (-1.46 to -1.03)
Dominica	5 (4 to 7)	8.953 (6.489 to 11.517)	8 (5 to 11)	9.823 (6.138 to 13.467)	0.28 (0.09 to 0.47)
Dominican Republic	476 (319 to 616)	9.144 (6.092 to 11.848)	905 (571 to 1196)	8.329 (5.314 to 10.973)	-0.26 (-0.94 to 0.42)
Ecuador	1192 (1089 to 1308)	16.748 (15.330 to 18.452)	1780 (1416 to 2224)	10.406 (8.294 to 12.961)	-1.09 (-2.53 to 0.38)
Egypt	12217 (7632 to 16713)	31.947 (21.646 to 42.111)	16172 (10877 to 20720)	20.343 (14.531 to 25.822)	-1.41 (-1.72 to -1.09)
El Salvador	46 (31 to 63)	1.409 (0.952 to 1.932)	90 (53 to 127)	1.418 (0.819 to 2.016)	0.03 (-0.39 to 0.45)
Equatorial Guinea	55 (25 to 86)	24.640 (11.810 to 37.398)	101 (53 to 167)	14.557 (8.300 to 23.843)	-1.59 (-2.01 to -1.17)
Eritrea	445 (216 to 711)	26.251 (13.404 to 40.380)	681 (427 to 1025)	17.651 (11.477 to 26.333)	-1.30 (-1.45 to -1.14)
Estonia	217 (168 to 278)	11.225 (8.766 to 14.229)	470 (389 to 592)	17.791 (14.945 to 21.747)	1.56 (0.10 to 3.03)
Eswatini	69 (45 to 98)	15.629 (9.791 to 22.294)	134 (77 to 197)	15.896 (9.597 to 23.548)	0.07 (-0.19 to 0.33)
Ethiopia	5244 (2094 to 8351)	18.868 (8.406 to 29.073)	5591 (3887 to 8553)	8.886 (6.081 to 13.706)	-2.40 (-2.54 to -2.27)
Fiji	22 (14 to 29)	3.709 (2.624 to 5.023)	25 (17 to 35)	3.292 (2.274 to 4.589)	-0.39 (-0.63 to -0.15)

Finland	2307 (1963 to 2713)	32.556 (27.853 to 38.133)	2811 (2429 to 3247)	20.762 (18.338 to 23.650)	-1.42 (-1.71 to -1.13)
France	13657 (12392 to 15300)	16.274 (14.925 to 17.991)	9596 (8293 to 11328)	6.117 (5.407 to 7.176)	-3.14 (-3.67 to -2.61)
Gabon	150 (84 to 212)	24.691 (13.769 to 35.030)	191 (110 to 284)	16.599 (9.713 to 24.049)	-1.25 (-1.48 to -1.03)
Gambia	76 (41 to 115)	16.864 (9.258 to 26.815)	205 (124 to 298)	15.920 (9.389 to 23.393)	-0.19 (-1.08 to 0.7)
Georgia	487 (303 to 770)	8.318 (5.176 to 13.110)	2678 (2203 to 3333)	45.589 (37.782 to 56.219)	5.62 (4.43 to 6.82)
Germany	42969 (37904 to 48339)	33.944 (30.003 to 37.950)	44359 (38244 to 49452)	20.458 (18.051 to 22.658)	-1.63 (-1.97 to -1.29)
Ghana	1907 (1052 to 2701)	23.972 (13.411 to 33.828)	3150 (1880 to 4593)	15.507 (9.028 to 23.193)	-1.38 (-1.58 to -1.17)
Greece	2340 (2153 to 2547)	16.000 (14.727 to 17.301)	3410 (2973 to 3866)	12.867 (11.379 to 14.351)	-0.67 (-1.22 to -0.11)
Greenland	17 (10 to 23)	49.593 (29.484 to 69.237)	16 (11 to 22)	26.580 (18.675 to 38.166)	-1.93 (-2.09 to -1.77)
Grenada	21 (17 to 25)	30.372 (25.208 to 35.768)	38 (33 to 44)	32.709 (27.718 to 37.318)	0.49 (0.15 to 0.84)
Guam	3 (1 to 4)	3.116 (1.884 to 4.668)	3 (2 to 6)	1.715 (0.987 to 2.841)	-1.95 (-2.50 to -1.39)
Guatemala	182 (147 to 230)	4.458 (3.565 to 5.591)	538 (459 to 625)	4.472 (3.831 to 5.175)	-0.35 (-0.90 to 0.19)
Guinea	601 (296 to 918)	16.572 (8.423 to 25.168)	1057 (603 to 1621)	14.745 (8.846 to 23.045)	-0.38 (-0.52 to -0.23)

Guinea-Bissau	141 (67 to 219)	27.579 (14.503 to 41.776)	222 (128 to 325)	21.231 (12.171 to 30.095)	-0.84 (-0.91 to -0.77)
Guyana	142 (117 to 167)	27.611 (23.448 to 31.730)	191 (145 to 250)	26.195 (20.065 to 34.076)	0.06 (-0.71 to 0.84)
Haiti	1170 (570 to 1953)	27.358 (13.858 to 43.915)	2196 (1203 to 3451)	22.071 (12.480 to 34.150)	-0.62 (-0.76 to -0.48)
Honduras	201 (108 to 278)	8.357 (4.569 to 11.510)	621 (350 to 914)	9.355 (5.273 to 13.426)	0.47 (-0.01 to 0.94)
Hungary	6456 (5900 to 7164)	45.531 (41.893 to 50.153)	7176 (6332 to 8105)	38.234 (33.899 to 42.720)	-0.75 (-1.58 to 0.09)
Iceland	43 (39 to 47)	14.670 (13.347 to 16.051)	54 (47 to 61)	9.140 (8.013 to 10.275)	-1.54 (-2.04 to -1.03)
India	47782 (27359 to 72659)	8.955 (5.278 to 12.931)	108780 (76289 to 137786)	8.806 (6.156 to 11.169)	-0.04 (-0.23 to 0.15)
Indonesia	3192 (1844 to 8389)	3.209 (1.846 to 8.111)	6214 (3308 to 16685)	3.011 (1.650 to 7.449)	-0.20 (-0.27 to -0.13)
Iran (Islamic Republic of)	6525 (4793 to 8295)	20.520 (14.566 to 25.659)	11189 (8664 to 13856)	13.735 (10.442 to 16.836)	-1.32 (-1.45 to -1.19)
Iraq	773 (372 to 1798)	8.406 (4.306 to 18.221)	1779 (797 to 4402)	6.691 (3.230 to 15.656)	-0.71 (-0.80 to -0.63)
Ireland	832 (754 to 922)	20.514 (18.745 to 22.489)	725 (622 to 844)	9.083 (7.850 to 10.485)	-2.57 (-3.03 to -2.11)
Israel	1620 (1469 to 1781)	33.390 (30.315 to 36.646)	1879 (1650 to 2141)	14.570 (12.881 to 16.502)	-2.65 (-3.24 to -2.05)
Italy	32892 (28531 to 39194)	37.616 (33.082 to 44.273)	37679 (29333 to 50845)	22.959 (18.198 to 30.493)	-1.57 (-2.03 to -1.11)

Jamaica	123 (108 to 139)	6.627 (5.829 to 7.449)	262 (205 to 336)	8.426 (6.584 to 10.817)	1.69 (0.50 to 2.9)
Japan	52996 (48213 to 58944)	32.864 (29.896 to 36.439)	63453 (51445 to 78386)	13.222 (10.899 to 16.445)	-2.91 (-3.13 to -2.70)
Jordan	232 (164 to 310)	12.419 (8.795 to 16.444)	523 (347 to 688)	5.933 (4.007 to 7.746)	-2.37 (-2.80 to -1.95)
Kazakhstan	799 (510 to 1224)	7.001 (4.411 to 10.960)	2030 (1536 to 2633)	12.256 (9.183 to 16.288)	1.83 (1.24 to 2.43)
Kenya	1244 (754 to 1704)	10.623 (6.455 to 14.910)	3057 (2157 to 4327)	10.470 (7.223 to 15.142)	-0.04 (-0.17 to 0.09)
Kiribati	9 (6 to 14)	12.700 (8.966 to 20.879)	15 (9 to 27)	13.125 (8.242 to 22.640)	0.10 (0.04 to 0.15)
Kuwait	66 (62 to 72)	7.005 (6.307 to 7.772)	186 (155 to 225)	5.059 (4.191 to 6.134)	-0.87 (-2.93 to 1.23)
Kyrgyzstan	217 (143 to 327)	7.687 (4.971 to 11.888)	382 (277 to 537)	8.777 (6.141 to 12.790)	0.25 (-0.90 to 1.43)
Lao People's Democratic Republic	80 (40 to 221)	3.778 (2.041 to 9.726)	134 (70 to 376)	2.998 (1.665 to 7.431)	-0.75 (-0.83 to -0.67)
Latvia	215 (162 to 305)	6.251 (4.773 to 8.756)	354 (286 to 463)	9.627 (7.927 to 12.160)	1.42 (0.62 to 2.23)
Lebanon	846 (434 to 1436)	36.115 (18.630 to 60.761)	570 (397 to 804)	9.462 (6.586 to 13.473)	-4.24 (-4.59 to -3.89)
Lesotho	111 (74 to 156)	11.394 (7.610 to 16.041)	252 (156 to 355)	17.714 (10.886 to 25.597)	1.47 (1.12 to 1.82)
Liberia	223 (107 to 344)	16.656 (8.299 to 26.280)	396 (204 to 654)	13.149 (7.106 to 22.459)	-0.76 (-0.94 to -0.57)

Libya	525 (317 to 956)	20.908 (13.495 to 36.834)	1607 (783 to 3439)	24.838 (12.478 to 52.948)	0.56<br>(0.29 to 0.83)
Lithuania	285 (201 to 419)	6.489 (4.603 to 9.519)	571 (443 to 772)	10.258 (8.187 to 13.581)	1.48<br>(0.78 to 2.19)
Luxembourg	185 (167 to 206)	34.325 (31.351 to 37.980)	181 (155 to 212)	16.086 (13.843 to 18.909)	-2.44<br>(-3.07 to -1.81)
Madagascar	2467 (1243 to 3505)	33.551 (18.575 to 46.539)	4786 (2919 to 6756)	26.314 (16.753 to 37.799)	-0.81<br>(-1.08 to -0.54)
Malawi	739 (327 to 1135)	13.693 (6.596 to 21.699)	1351 (841 to 2089)	12.442 (7.646 to 20.312)	-0.30<br>(-0.49 to -0.1)
Malaysia	531 (377 to 686)	5.137 (3.663 to 6.785)	1239 (905 to 1594)	4.482 (3.335 to 5.877)	-0.41<br>(-0.63 to -0.20)
Maldives	10 (5 to 16)	9.053 (5.462 to 14.598)	20 (14 to 27)	5.790 (4.015 to 7.828)	-1.51<br>(-1.69 to -1.33)
Mali	792 (343 to 1250)	16.465 (7.816 to 25.358)	1474 (796 to 2153)	11.948 (6.724 to 17.940)	-1.00<br>(-1.16 to -0.84)
Malta	72 (66 to 79)	17.468 (16.049 to 19.064)	99 (87 to 114)	9.929 (8.789 to 11.327)	-1.84<br>(-2.23 to -1.45)
Marshall Islands	2 (1 to 3)	5.403 (3.595 to 8.160)	2 (1 to 4)	4.538 (2.712 to 7.592)	-0.56<br>(-0.63 to -0.49)
Mauritania	239 (133 to 328)	20.951 (11.750 to 29.256)	315 (188 to 461)	12.799 (7.573 to 19.326)	-1.59<br>(-1.84 to -1.35)
Mauritius	123 (114 to 134)	15.268 (14.064 to 16.730)	233 (214 to 253)	13.937 (12.810 to 15.057)	0.50<br>(-5.42 to 6.8)
Mexico	3962 (3846 to 4094)	8.095 (7.827 to 8.451)	6604 (5886 to 7445)	5.137 (4.587 to 5.799)	-1.68<br>(-3.78 to 0.48)

Micronesia (Federated States of)	5 (3 to 7)	5.860 (3.884 to 8.595)	3 (2 to 6)	3.676 (2.376 to 5.977)	-1.49 (-1.54 to -1.44)
Monaco	8 (5 to 11)	10.774 (7.421 to 14.775)	9 (6 to 13)	8.889 (5.763 to 12.459)	-0.63 (-0.72 to -0.53)
Mongolia	209 (151 to 291)	19.435 (13.814 to 27.632)	419 (315 to 567)	19.087 (13.907 to 26.141)	-0.07 (-0.29 to 0.14)
Montenegro	79 (58 to 101)	12.884 (9.484 to 16.558)	126 (90 to 164)	13.521 (9.548 to 17.544)	0.23 (-0.03 to 0.49)
Morocco	4578 (2771 to 8268)	27.211 (16.807 to 47.041)	8831 (5534 to 16388)	24.681 (15.830 to 45.353)	-0.30 (-0.36 to -0.23)
Mozambique	1199 (568 to 1946)	16.537 (8.002 to 27.562)	2801 (1454 to 4472)	18.141 (9.479 to 31.773)	0.32 (0.16 to 0.48)
Myanmar	907 (489 to 2334)	3.679 (2.095 to 8.877)	1334 (743 to 3449)	2.954 (1.641 to 7.318)	-0.73 (-0.78 to -0.68)
Namibia	135 (91 to 173)	15.719 (10.421 to 19.931)	229 (146 to 356)	12.869 (8.451 to 19.697)	-0.57 (-0.86 to -0.29)
Nauru	1 (0 to 1)	7.771 (5.170 to 11.376)	1 (0 to 1)	5.222 (3.150 to 9.722)	-1.31 (-1.51 to -1.12)
Nepal	904 (484 to 1461)	8.341 (4.789 to 12.756)	1892 (1143 to 2754)	7.824 (4.788 to 11.367)	-0.20 (-0.33 to -0.07)
Netherlands	16555 (15282 to 17695)	81.276 (75.254 to 86.481)	11019 (9604 to 12276)	30.032 (26.581 to 33.121)	-3.17 (-3.62 to -2.72)
New Zealand	1190 (1088 to 1321)	30.828 (28.196 to 33.963)	1607 (1391 to 1872)	18.767 (16.398 to 21.663)	-1.60 (-2.59 to -0.61)
Nicaragua	49 (37 to 66)	2.687 (2.009 to 3.620)	157 (87 to 208)	2.990 (1.665 to 3.957)	0.21 (-0.11 to 0.54)

Niger	533 (247 to 875)	15.561 (7.666 to 25.219)	1208 (603 to 2056)	11.749 (6.068 to 20.881)	-0.89<br>(-0.99 to -0.8)
Nigeria	9164 (5188 to 13708)	18.249 (10.220 to 27.558)	12811 (7013 to 21126)	11.277 (6.382 to 18.365)	-1.55<br>(-1.66 to -1.45)
Niue	0 (0 to 0)	4.234 (2.934 to 6.209)	0 (0 to 0)	4.295 (2.477 to 8.367)	0.11<br>(-0.47 to 0.69)
North Macedonia	248 (194 to 318)	13.922 (10.844 to 18.100)	449 (279 to 616)	14.882 (9.070 to 20.429)	0.24<br>(-0.16 to 0.64)
Northern Mariana Islands	0 (0 to 1)	2.119 (1.243 to 3.458)	1 (0 to 1)	1.427 (0.825 to 2.423)	-1.30<br>(-1.65 to -0.94)
Norway	2894 (2480 to 3522)	37.877 (32.696 to 45.736)	2104 (1582 to 2927)	18.327 (13.687 to 25.633)	-2.36<br>(-2.96 to -1.76)
Oman	120 (79 to 171)	13.517 (8.894 to 19.028)	185 (127 to 234)	7.502 (5.150 to 9.348)	-1.86<br>(-2.19 to -1.54)
Pakistan	5288 (3324 to 7808)	8.298 (5.188 to 11.831)	15345 (9264 to 22491)	10.234 (6.443 to 14.392)	0.70<br>(0.63 to 0.77)
Palau	1 (0 to 1)	4.976 (3.313 to 7.266)	1 (0 to 1)	4.812 (2.809 to 8.169)	-0.12<br>(-0.25 to 0)
Palestine	92 (57 to 132)	9.103 (5.692 to 12.650)	171 (121 to 219)	5.858 (4.153 to 7.552)	-1.45<br>(-1.64 to -1.26)
Panama	95 (85 to 105)	5.850 (5.223 to 6.506)	474 (380 to 573)	10.701 (8.568 to 12.940)	1.93<br>(1.46 to 2.41)
Papua New Guinea	147 (61 to 333)	4.451 (2.400 to 8.640)	284 (157 to 499)	3.408 (2.088 to 5.569)	-0.85<br>(-1.13 to -0.57)
Paraguay	349 (231 to 459)	13.103 (8.770 to 17.462)	934 (589 to 1278)	14.803 (9.407 to 20.141)	0.38<br>(-0.25 to 1.02)

Peru	593 (382 to 828)	3.804 (2.490 to 5.270)	1081 (635 to 1487)	3.043 (1.788 to 4.182)	-0.64 (-1.87 to 0.60)
Philippines	673 (433 to 896)	2.325 (1.530 to 3.192)	1909 (1101 to 2660)	2.417 (1.454 to 3.424)	0.14 (-0.05 to 0.33)
Poland	4750 (4371 to 5336)	10.939 (10.055 to 12.295)	15678 (14179 to 17264)	22.308 (20.251 to 24.550)	2.13 (1.27 to 3.00)
Portugal	2282 (2149 to 2424)	17.522 (16.551 to 18.505)	2806 (2456 to 3119)	10.659 (9.558 to 11.692)	-1.60 (-2.51 to -0.67)
Puerto Rico	424 (395 to 454)	11.792 (10.995 to 12.623)	431 (362 to 511)	8.341 (6.927 to 9.870)	-0.99 (-2.50 to 0.54)
Qatar	34 (26 to 49)	20.617 (15.377 to 29.083)	121 (73 to 172)	8.989 (5.887 to 12.330)	-2.77 (-3.30 to -2.24)
Republic of Korea	3264 (2195 to 4326)	12.798 (8.796 to 17.311)	6085 (4072 to 8631)	6.673 (4.466 to 9.367)	-2.12 (-2.36 to -1.88)
Republic of Moldova	241 (175 to 341)	6.033 (4.268 to 8.652)	511 (416 to 644)	8.910 (7.298 to 11.133)	1.25 (0.34 to 2.16)
Romania	5543 (4733 to 6439)	20.244 (17.327 to 23.617)	5299 (4454 to 6382)	14.659 (12.452 to 17.423)	-1.10 (-1.66 to -0.53)
Russian Federation	8855 (6332 to 12961)	5.293 (3.818 to 7.718)	23469 (19431 to 29276)	10.463 (8.794 to 12.857)	2.35 (1.60 to 3.10)
Rwanda	1357 (612 to 2006)	32.942 (17.180 to 47.475)	1057 (667 to 1645)	12.999 (8.239 to 20.770)	-2.98 (-3.12 to -2.83)
Saint Kitts and Nevis	7 (6 to 8)	19.447 (16.304 to 22.930)	9 (7 to 11)	13.212 (10.887 to 15.701)	-1.10 (-2.18 to -0.02)
Saint Lucia	8 (7 to 9)	8.646 (7.645 to 9.812)	14 (11 to 17)	6.064 (5.020 to 7.395)	-1.15 (-2.02 to -0.26)

Saint Vincent and the Grenadines	8 (7 to 9)	9.951 (8.659 to 11.256)	25 (22 to 28)	18.344 (15.979 to 21.018)	1.90 (0.66 to 3.15)
Samoa	5 (3 to 7)	3.834 (2.678 to 5.312)	5 (3 to 9)	3.056 (1.943 to 5.044)	-0.73 (-0.82 to -0.63)
San Marino	3 (2 to 4)	9.411 (6.548 to 11.988)	5 (3 to 7)	5.857 (3.822 to 8.412)	-1.76 (-1.94 to -1.57)
Sao Tome and Principe	9 (4 to 15)	12.113 (6.173 to 21.303)	16 (10 to 26)	11.944 (7.683 to 18.792)	0.01 (-0.24 to 0.27)
Saudi Arabia	1767 (1249 to 2421)	20.877 (14.839 to 28.520)	5405 (3475 to 7539)	17.165 (11.688 to 23.021)	-0.64 (-0.81 to -0.47)
Senegal	683 (373 to 1004)	17.368 (9.460 to 25.805)	1315 (850 to 2030)	14.317 (9.146 to 23.119)	-0.67 (-0.85 to -0.50)
Serbia	6657 (5357 to 8541)	65.109 (52.239 to 84.220)	9083 (6481 to 11466)	54.545 (39.318 to 68.462)	-0.52 (-0.89 to -0.15)
Seychelles	6 (4 to 10)	9.990 (6.890 to 16.098)	7 (5 to 13)	6.737 (4.340 to 11.707)	-1.24 (-1.52 to -0.96)
Sierra Leone	360 (191 to 552)	15.320 (8.406 to 23.806)	675 (358 to 1081)	13.459 (7.544 to 21.933)	-0.42 (-0.56 to -0.28)
Singapore	421 (375 to 489)	20.042 (17.673 to 23.708)	598 (472 to 796)	7.250 (5.729 to 9.649)	-3.29 (-3.68 to -2.89)
Slovakia	856 (676 to 1094)	14.469 (11.442 to 18.457)	1161 (707 to 1540)	12.545 (7.659 to 16.500)	-0.47 (-0.65 to -0.29)
Slovenia	988 (877 to 1121)	40.457 (35.988 to 45.710)	1300 (1086 to 1582)	27.848 (23.167 to 33.724)	-1.22 (-2.06 to -0.37)
Solomon Islands	6 (3 to 10)	3.025 (1.865 to 4.733)	11 (7 to 19)	2.392 (1.514 to 3.682)	-0.72 (-0.82 to -0.63)

Somalia	716 (264 to 1273)	19.070 (7.944 to 32.327)	1201 (552 to 1995)	12.213 (5.670 to 21.636)	-1.40 (-1.47 to -1.34)
South Africa	4906 (3046 to 5904)	15.831 (9.748 to 18.802)	5727 (4373 to 7410)	10.619 (7.895 to 13.811)	-1.48 (-1.93 to -1.02)
South Sudan	591 (282 to 931)	17.798 (8.952 to 27.466)	645 (334 to 1025)	12.017 (6.284 to 19.655)	-1.23 (-1.38 to -1.09)
Spain	13427 (12377 to 14359)	25.494 (23.582 to 27.156)	16542 (13992 to 18541)	14.993 (13.007 to 16.647)	-1.60 (-2.00 to -1.20)
Sri Lanka	468 (263 to 657)	4.234 (2.553 to 6.050)	662 (438 to 1001)	2.653 (1.791 to 3.977)	-1.47 (-1.80 to -1.13)
Sudan	3760 (1906 to 6990)	32.282 (17.797 to 57.916)	6166 (3264 to 12322)	23.459 (13.459 to 44.644)	-1.03 (-1.08 to -0.99)
Suriname	67 (45 to 82)	21.691 (15.080 to 26.535)	113 (75 to 146)	17.803 (11.873 to 23.027)	-0.57 (-0.99 to -0.14)
Sweden	2951 (2644 to 3398)	18.218 (16.451 to 20.757)	2659 (2183 to 3255)	10.417 (8.553 to 12.720)	-1.88 (-2.21 to -1.55)
Switzerland	2765 (2438 to 3141)	25.816 (22.959 to 29.051)	2561 (2160 to 3065)	12.615 (10.793 to 15.015)	-2.31 (-2.71 to -1.90)
Syrian Arab Republic	3322 (2147 to 5787)	50.711 (32.938 to 88.426)	4041 (2437 to 8113)	30.635 (19.019 to 60.587)	-1.64 (-1.96 to -1.33)
Taiwan (Province of China)	4048 (3818 to 4362)	24.776 (23.119 to 27.240)	3899 (3376 to 4634)	9.632 (8.435 to 11.318)	-2.71 (-4.39 to -1.00)
Tajikistan	143 (79 to 253)	6.060 (3.376 to 10.780)	250 (130 to 432)	6.265 (3.398 to 10.823)	0.11 (0.05 to 0.18)
Thailand	1883 (1381 to 3030)	4.858 (3.531 to 7.765)	7235 (4355 to 10147)	7.427 (4.568 to 10.326)	1.31 (0.82 to 1.81)

Timor-Leste	8 (5 to 22)	2.927 (1.662 to 6.948)	23 (12 to 61)	2.746 (1.459 to 6.796)	-0.19 (-0.33 to -0.05)
Togo	301 (157 to 430)	18.140 (9.839 to 26.387)	737 (425 to 1111)	15.539 (9.189 to 24.297)	-0.49 (-0.68 to -0.30)
Tokelau	0 (0 to 0)	5.121 (3.504 to 7.955)	0 (0 to 0)	4.716 (2.896 to 8.946)	0.09 (-0.36 to 0.55)
Tonga	2 (1 to 3)	2.596 (1.791 to 3.824)	2 (1 to 3)	1.999 (1.205 to 3.208)	-0.84 (-0.94 to -0.75)
Trinidad and Tobago	144 (132 to 157)	15.035 (13.785 to 16.468)	241 (187 to 304)	13.816 (10.801 to 17.534)	-0.07 (-0.39 to 0.24)
Tunisia	1211 (798 to 2159)	21.917 (14.320 to 37.840)	2236 (1257 to 4427)	17.008 (9.655 to 33.563)	-0.84 (-0.91 to -0.77)
Turkey	20367 (12965 to 27418)	50.814 (32.639 to 67.671)	22108 (17038 to 29962)	23.572 (18.120 to 32.383)	-2.45 (-2.75 to -2.15)
Turkmenistan	102 (61 to 165)	6.443 (3.716 to 10.468)	355 (256 to 501)	9.803 (6.696 to 14.389)	1.31 (0.61 to 2.02)
Tuvalu	0 (0 to 1)	5.892 (3.666 to 9.323)	0 (0 to 1)	3.401 (2.150 to 5.676)	-1.75 (-1.82 to -1.67)
Uganda	1192 (620 to 1952)	14.137 (7.609 to 23.990)	2375 (1407 to 3828)	10.926 (6.741 to 18.123)	-0.85 (-0.97 to -0.72)
Ukraine	2467 (1547 to 3997)	3.644 (2.348 to 5.852)	4363 (3123 to 6056)	6.023 (4.441 to 8.208)	1.71 (1.15 to 2.27)
United Arab Emirates	100 (58 to 145)	13.956 (8.251 to 20.062)	286 (187 to 446)	6.404 (4.376 to 9.337)	-2.26 (-3.19 to -1.33)
United Kingdom	15840 (14364 to 17931)	17.544 (16.085 to 19.577)	17823 (15480 to 21498)	13.047 (11.474 to 15.520)	-1.00 (-1.45 to -0.54)

United Republic of Tanzania	2408 (1306 to 3575)	17.525 (9.971 to 27.577)	4142 (2412 to 6619)	12.433 (7.339 to 20.857)	-1.10 (-1.17 to -1.03)
United States of America	86294 (75579 to 102458)	26.475 (23.404 to 31.084)	107178 (87494 to 138274)	18.070 (14.932 to 22.938)	-1.20 (-1.51 to -0.89)
United States Virgin Islands	33 (23 to 42)	33.455 (23.743 to 42.250)	21 (15 to 29)	17.551 (12.293 to 24.766)	-2.00 (-2.21 to -1.78)
Uruguay	686 (613 to 773)	18.113 (16.257 to 20.289)	830 (740 to 954)	15.422 (13.875 to 17.451)	-0.58 (-1.45 to 0.29)
Uzbekistan	942 (672 to 1366)	8.171 (5.608 to 12.190)	3096 (2314 to 4234)	13.327 (9.496 to 18.772)	1.46 (0.57 to 2.35)
Vanuatu	4 (3 to 7)	4.035 (2.612 to 6.146)	8 (4 to 15)	3.205 (1.958 to 5.562)	-0.79 (-1.04 to -0.54)
Venezuela (Bolivarian Republic of)	1286 (1137 to 1441)	10.988 (9.699 to 12.392)	4595 (3473 to 5953)	15.504 (11.760 to 19.975)	1.06 (-0.05 to 2.18)
Viet Nam	1073 (595 to 2655)	2.711 (1.500 to 6.728)	2244 (1181 to 5818)	2.546 (1.343 to 6.552)	-0.20 (-0.26 to -0.13)
Yemen	1878 (1024 to 3323)	32.229 (18.801 to 55.882)	4820 (2708 to 8122)	27.594 (16.217 to 45.244)	-0.46 (-0.73 to -0.18)
Zambia	906 (406 to 1300)	21.322 (11.068 to 30.157)	1805 (1152 to 2722)	17.788 (11.627 to 26.870)	-0.59 (-0.80 to -0.38)
Zimbabwe	648 (365 to 1112)	11.284 (6.411 to 17.816)	2053 (1235 to 3170)	18.662 (11.258 to 26.300)	1.80 (1.17 to 2.43)

Abbreviations: AAPC, annual percentage change; CI, confidence interval; DALYs, disability-adjusted life years; DMVD, degenerative mitral valve disease; UI, uncertain interval.

Supplementary Table 4: Age-standardized prevalence rates and average annual percentage change for DMVD at sex and SDI levels, 1990-2021

		1990		2021		1990 – 2021
		**Cases (95% UI)**	**Age-standardized prevalence per 100 000 population (95% UI)**	**Cases (95% UI)**	**Age- standardized prevalence per 100 000 population (95% UI)**	**AAPC % (95% CI)**
**Male**	High SDI	2361618 (2190355 to 2555298)	514.125 (477.427 to 556.003)	5298979 (4956078 to 5709366)	530.393 (496.592 to 571.206)	0.11 (0.04 to 0.19)
	High-middle SDI	1382759 (1278529 to 1510309)	364.732 (337.932 to 396.160)	2819728 (2633384 to 3034388)	327.629 (306.008 to 351.930)	-0.34 (-0.39 to -0.29)
	Middle SDI	420976 (380125 to 468984)	100.303 (90.992 to 110.691)	1320155 (1225722 to 1442596)	111.266 (103.522 to 120.794)	0.32 (0.31 to 0.34)
	Low-middle SDI	128389 (111360 to 147185)	48.081 (42.042 to 54.685)	321362 (283764 to 365704)	50.905 (44.998 to 57.481)	0.20 (0.10 to 0.30)
	Low SDI	25582 (21962 to 29595)	25.063 (21.714 to 28.589)	59970 (52497 to 68433)	26.825 (23.544 to 30.332)	0.25 (0.12 to 0.38)
**Female**	High SDI	1508588 (1403310 to 1619742)	219.114 (203.460 to 235.636)	2850346 (2665548 to 3039340)	227.592 (213.129 to 242.721)	0.14 (0.10 to 0.18)
	High-middle SDI	865304 (796034 to 938880)	153.289 (141.020 to 166.127)	1666733 (1554460 to 1782005)	145.693 (135.958 to 155.817)	-0.15 (-0.20 to -0.10)
	Middle SDI	278411 (253576to 306214)	58.110 (52.940 to 63.488)	811412 (753149 to 881731)	59.280 (55.057 to 64.311)	0.05 (0.01 to 0.08)
	Low-middle SDI	1077951 (94964 to 120651)	40.450 (35.554 to 45.540)	275879 (246333 to 307199)	39.164 (35.037 to 43.348)	-0.11 (-0.13 to -0.09)
	Low SDI	23837 (20778 to 27321)	23.242 (20.464 to 26.149)	55544 (49330 to 62865)	22.905 (20.478 to 25.642)	-0.05 (-0.09 to -0.02)

Abbreviations: AAPC, annual percentage change; CI, confidence interval; DMVD, degenerative mitral valve disease; SDI, sociodemographic index; UI, uncertain interval.

Supplementary Table 5: Age-standardized mortality rates and average annual percentage change for DMVD at sex and SDI levels, 1990-2021

		1990		2021		1990 – 2021
		**Cases (95% UI)**	**Age-standardized mortality per 100 000 population (95% UI)**	**Cases (95% UI)**	**Age- standardized mortality per 100 000 population (95% UI)**	**AAPC % (95% CI)**
**Male**	High SDI	4254 (4044 to 4412)	1.032 (0.971 to 1.075)	5861 (5294 to 6189)	0.604 (0.549 to 0.638)	-1.68 (-1.94 to -1.43)
	High-middle SDI	1621 (1411 to 1780)	0.459 (0.406 to 0.504)	2621 (2350 to 2887)	0.341 (0.306 to 0.375)	-0.92 (-1.14 to -0.70)
	Middle SDI	1116 (853 to 1299)	0.249 (0.187 to 0.294)	2406 (2023 to 3053)	0.214 (0.178 to 0.273)	-0.50 (-0.74 to -0.26)
	Low-middle SDI	970 (662 to 1378)	0.335 (0.226 to 0.476)	2203 (1644 to 3083	0.348 (0.260 to 0.496)	0.18 (0.08 to 0.28)
	Low SDI	584.118 (327.069 to 874.040)	0.552 (0.301 to 0.819)	1056 (681 to 1538)	0.455 (0.288 to 0.657)	-0.60 (-0.77 to -0.44)
**Female**	High SDI	8753 (7753 to 9387)	1.257 (1.119 to 1.346)	10404 (8147 to 11713)	0.661 (0.537 to 0.733)	-2.08 (-2.32 to -1.84)
	High-middle SDI	2822 (2450 to 3151)	0.542 (0.469 to 0.605)	4266 (3561 to 4992)	0.377 (0.316 to 0.443)	-1.16 (-1.31 to -1.01)
	Middle SDI	1590 (1211 to 1985)	0.313 (0.234 to 0.406)	3286 (2523 to 4542)	0.246 (0.188 to 0.340)	-0.82 (-1.10 to -0.53)
	Low-middle SDI	1459 (850 to 2102)	0.494 (0.288 to 0.742)	3262 (2056to 4440)	0.456 (0.286 to 0.630)	-0.24 (-0.53 to 0.05)
	Low SDI	752 (331 to 1193)	0.687 (0.299 to 1.187)	1423 (755 to 2146)	0.562 (0.286 to 0.911)	-0.64 (-0.76 to -0.52)

Abbreviations: AAPC, annual percentage change; CI, confidence interval; DMVD, degenerative mitral valve disease; SDI, sociodemographic index; UI, uncertain interval.

Supplementary Table 6: Age-standardized DALYs rates and average annual percentage change for DMVD at sex and SDI levels, 1990-2021

		1990		2021		1990 – 2021
		**Cases (95% UI)**	**Age-standardized DALYs per 100 000 population (95% UI)**	**Cases (95% UI)**	**Age- standardized DALYs per 100 000 population (95% UI)**	**AAPC % (95% CI)**
Men	High SDI	117708 (103891 to 137463)	26.522 (23.353 to 31.147)	160607 (131442 to 205513)	16.936 (14.061 to 21.319)	-1.44 (-1.60 to -1.27)
	High-middle SDI	59880 (49330 to 73804)	15.244 (12.439 to 19.138)	89367 (72850 to 113315)	11.023 (8.988 to 13.951)	-1.03 (-1.19 to -0.88)
	Middle SDI	42035 (33581 to 49060)	7.698 (6.140 to 9.129)	81193 (68142 to 100149)	6.676 (5.570 to 8.204)	-0.46 (-0.68 to -0.25)
	Low-middle SDI	32507 (22808 to 45732)	9.179 (6.426 to 12.982)	67870 (51374 to 89279)	9.158 (6.957 to 12.136)	0.04 (0.01 to 0.06)
	Low SDI	18751 (10780 to 27672)	14.050 (8.066 to 20.795)	34254 (22736 to 48789)	11.353 (7.495 to 16.340)	-0.67 (-0.79 to -0.55)
Women	High SDI	168889 (153388to 186026)	25.673 (23.499 to 28.099)	174088.103 (146629 to 203500)	13.387 (11.523 to 15.501)	-2.04 (-2.24 to -1.83)
	High-middle SDI	74207 (62880 to 86312)	13.564 (11.470 to 15.776)	95282(81029 to 116743)	8.876 (7.582 to 10.869)	-1.38 (-1.48 to -1.29)
	Middle SDI	55083 (43574 to 69653)	9.038 (7.213 to 11.182)	94681 (76578 to 127526)	6.918 (5.591 to 9.354)	-0.88 (-1.08 to -0.68)
	Low-middle SDI	49781 (29230 to 76891)	13.597 (8.137 to 19.829)	97243 (64141 to 127370)	12.022 (7.869 to 15.956)	-0.39 (-0.49 to -0.29)
	Low SDI	26127 (10964 to 41390)	18.262 (8.207 to 28.728)	47323 (26285 to 67197)	14.186 (7.825 to 21.059)	-0.80 (-0.85 to -0.76)

Abbreviations: AAPC, annual percentage change; CI, confidence interval; DALYs, disability-adjusted life years; DMVD, degenerative mitral valve disease; SDI, sociodemographic index; UI, uncertain interval.

Supplementary Table 7: The prevalence rates and rate change for DMVD at sex and age levels, 1990-2021

		1990		2021		1990 – 2021
**Sex**	**Age**	**Cases (95% UI)**	**Rate (95% UI)**	**Cases (95% UI)**	**Rate (95% UI)**	**RC per 100,000 population**
Both	0-4	..	..	..	..	..
	5-9	..	..	..	..	..
	10-14	..	..	..	..	..
	15-19	951 (745 to 1188)	0.183 (0.144 to 0.229)	946 (747 to 1193)	0.152 (0.120 to 0.191)	-0.031
	20-24	6136 (4824 to 7659)	1.247 (0.980 to 1.556)	6148 (4869 to 7716)	1.030 (0.815 to 1.292)	-0.217
	25-29	16098 (12779 to 20021)	3.637 (2.887 to 4.523)	16615 (13238 to 20722)	2.824 (2.250 to 3.522)	-0.813
	30-34	29589 (23571 to 36603)	7.677 (6.116 to 9.497)	33601 (26886 to 41719)	5.559 (4.448 to 6.902)	-2.118
	35-39	44901 (35751 to 55265)	12.747 (10.149 to 15.689)	53797 (43257 to 66509)	9.592 (7.712 to 11.858)	-3.155
	40-44	62227 (51772 to 75212)	21.721 (18.072 to 26.254)	79713 (66832 to 95454)	15.935 (13.360 to 19.081)	-5.786
	45-49	84228 (67985 to 102439)	36.274 (29.279 to 44.117)	125878 (102581 to 151859)	26.584 (21.664 to 32.071)	-9.690
	50-54	163415 (132785 to 207126)	76.875 (62.466 to 97.438)	238379 (194941 to 300372)	53.578 (43.814 to 67.511)	-23.297
	55-59	371597 (309952 to 456847)	200.647 (167.361 to 246.678)	582819 (494775 to 703864)	147.278 (125.029 to 177.866)	-53.369
	60-64	769186 (652641 to 927362)	478.918 (406.353 to 577.403)	1231968 (1066354 to 1456093)	384.932 (333.186 to 454.961)	-93.986
	65-69	1265714 (1151587 to 1409590)	1023.962 (931.634 to 1140.358)	2501089 (2316673 to 2740086)	906.710 (839.854 to 993.353)	-117.252
	70-74	1473057 (1361219 to 1590256)	1739.944 (1607.843 to 1878.378)	3633715 (3393547 to 3902759)	1765.317 (1648.640 to 1896.023)	25.373
	75-79	1440404 (1325751 to 1566720)	2340.008 (2153.749 to 2545.215)	2973841 (2759872 to 3196224)	2254.884 (2092.644 to 2423.504)	-85.124
	80-84	873294 (801732 to 952301)	2468.611 (2266.322 to 2691.947)	2192416 (2036952 to 2362417)	2503.242 (2325.738 to 2697.345)	34.631
	85-89	377009 (345934 to 412645)	2494.920 (2289.275 to 2730.747)	1192625 (1108730 to 1285467)	2608.438 (2424.949 to 2811.497)	113.518
	90-94	108205 (98678 to 118154)	2525.097 (2302.779 to 2757.258)	485250 (448558 to 523881)	2712.506 (2507.399 to 2928.450)	187.409
	95+	25747 (23540 to 28209)	2528.976 (2312.141 to 2770.762)	145847 (134542 to 157295)	2675.945 (2468.516 to 2885.978)	146.969

Male	0-4	..	..	..	..	..
	5-9	..	..	..	..	..
	10-14	..	..	..	..	..
	15-19	597 (474 to 743)	0.226 (0.180 to 0.282)	559 (446 to 697)	0.175 (0.139 to 0.217)	-0.051
	20-24	3921 (3131 to 4858)	1.581 (1.263 to 1.959)	3704 (2972 to 4595)	1.221 (0.979 to 1.515)	-0.360
	25-29	10475 (8439 to 12898)	4.707 (3.793 to 5.796)	10216 (8241 to 12653)	3.436 (2.771 to 4.255)	-1.271
	30-34	19562 (15830 to 23956)	10.016 (8.105 to 12.265)	21009 (17064 to 25958)	6.876 (5.585 to 8.495)	-3.140
	35-39	29904 (24178 to 36461)	16.726 (13.523 to 20.393)	33814 (27512 to 41628)	11.946 (9.720 to 14.706)	-4.780
	40-44	41969 (35463 to 50414)	28.696 (24.247 to 34.470)	50758 (43048 to 60182)	20.129 (17.071 to 23.866)	-8.567
	45-49	56748 (46004 to 68997)	47.931 (38.856 to 58.277)	82238 (67259 to 99882)	34.574 (28.277 to 41.992)	-13.357
	50-54	111611 (91743 to 140736)	103.675 (85.220 to 130.729)	161055 (133407 to 201733)	72.554 (60.099 to 90.879)	-31.121
	55-59	256709 (214532 to 316221)	276.364 (230.958 to 340.432)	403927 (342984 to 490593)	207.435 (176.138 to 251.942)	-68.929
	60-64	521214 (443481 to 631363)	663.577 (564.612 to 803.812)	845985 (732560 to 1001377)	543.913 (470.988 to 643.819)	-119.664
	65-69	810872 (738473 to 903363)	1414.364 (1288.082 to 1575.691)	1668243 (1544810 to 1830496)	1265.419 (1171.791 to 1388.494)	-148.945
	70-74	899613 (832021 to 974164)	2391.411 (2211.732 to 2589.587)	2361704 (2203086 to 2541523)	2450.119 (2285.562 to 2636.670)	58.708
	75-79	837242 (769785 to 915692)	3318.068 (3050.727 to 3628.969)	1880979 (1749520 to 2030103)	3146.149 (2926.268 to 3395.575)	-171.919
	80-84	475827 (437263 to 522034)	3582.111 (3291.790 to 3929.961)	1319914 (1227166 to 1425775)	3601.220 (3348.168 to 3890.047)	19.109
	85-89	188537 (172385 to 207597)	3723.034 (3404.092 to 4099.414)	672670 (625345 to 730212)	3898.937 (3624.627 to 4232.460)	175.903
	90-94	49019 (44732 to 54000)	3893.606 (3553.072 to 4289.292)	247358 (228951 to 268978)	4243.932 (3928.127 to 4614.869)	350.326
	95+	10694 (9810 to 11775)	4109.521 (3769.709 to 4524.830)	65203 (60235 to 70681)	4312.269 (3983.718 to 4674.571)	202.748
Female	0-4	..	..	..	..	..
	5-9	..	..	..	..	..
	10-14	..	..	..	..	..
	15-19	353 (270 to 454)	0.138 (0.106 to 0.178)	387 (299 to 492)	0.127 (0.099 to 0.162)	-0.011

	20-24	2215 (1693 to 2841)	0.907 (0.693 to 1.164)	2444 (1898 to 3099)	0.832 (0.646 to 1.055)	-0.075
	25-29	5623 (4307 to 7159)	2.555 (1.957 to 3.253)	6399 (4984 to 8082)	2.199 (1.713 to 2.778)	-0.356
	30-34	10026 (7721 to 12668)	5.274 (4.061 to 6.663)	12591 (9795 to 15824)	4.212 (3.277 to 5.293)	-1.062
	35-39	14997 (11543 to 18978)	8.646 (6.655 to 10.941)	19982 (15590 to 24913)	7.193 (5.612 to 8.968)	-1.453
	40-44	20258 (16179 to 24869)	14.446 (11.538 to 17.735)	28956 (23614 to 35011)	11.671 (9.518 to 14.112)	-2.775
	45-49	27480 (21746 to 33901)	24.147 (19.109 to 29.790)	43640 (35098 to 52994)	18.520 (14.895 to 22.489)	-5.627
	50-54	51804 (41174 to 66670)	49.377 (39.244 to 63.546)	77324 (61583 to 98666)	34.683 (27.623 to 44.256)	-14.694
	55-59	114889 (95015 to 140978)	124.457 (102.928 to 152.719)	178892 (150606 to 215937)	89.000 (74.927 to 107.429)	-35.457
	60-64	247973 (209853 to 298067)	302.173 (255.721 to 363.217)	385982 (333395 to 452616)	234.624 (202.658 to 275.128)	-67.549
	65-69	454842 (414476 to 505406)	686.261 (625.357 to 762.552)	832846 (771366 to 905831)	578.329 (535.638 to 629.010)	-107.932
	70-74	573444 (530750 to 618796)	1218.987 (1128.231 to 1315.394)	1272011 (1184471 to 1359372)	1162.208 (1082.224 to 1242.028)	-56.779
	75-79	603161 (554370 to 653154)	1660.564 (1526.236 to 1798.199)	1092861 (1015261 to 1170698)	1515.807 (1408.175 to 1623.767)	-144.757
	80-84	397467 (366145 to 432748)	1799.103 (1657.329 to 1958.800)	872501 (810766 to 933631)	1713.098 (1591.884 to 1833.122)	-86.005
	85-89	188473 (173416 to 205866)	1875.906 (1726.048 to 2049.024)	519954 (482488 to 558529)	1826.380 (1694.777 to 1961.876)	-49.526
	90-54	59186 (54046 to 64780)	1955.774 (1785.919 to 2140.625)	237893 (219403 to 255923)	1972.433 (1819.132 to 2121.926)	16.659
	95+	15053 (13711 to 16528)	1986.250 (1809.245 to 2180.836)	80644 (74105 to 86879)	2047.701 (1881.658 to 2206.019)	61.451

Abbreviations: DMVD, degenerative mitral valve disease; RC, rate change; UI, uncertain interval.

Supplementary Table 8: The mortality rates and rate change for DMVD at sex and age levels, 1990-2021

		1990		2021		1990– 2021
Sex	Age	Cases (95% UI)	Rate (95% UI)	Cases (95% UI)	Rate (95% UI)	RC per 100,000 population
Both	0-4	..	..	..	..	..
	5-9	..	..	..	..	..
	10-14	..	..	..	..	..
	15-19	263 (172 to 354)	0.051 (0.033 to 0.068)	260 (191 to 330)	0.042 (0.031 to 0.053)	-0.009
	20-24	326 (210 to 433)	0.066 (0.043 to 0.088)	348 (265 to 440)	0.058 (0.044 to 0.074)	-0.008
	25-29	352 (249 to 460)	0.080 (0.056 to 0.104)	376 (288 to 452)	0.064 (0.049 to 0.077)	-0.016
	30-34	375 (280 to 471)	0.097 (0.073 to 0.122)	447 (359 to 542)	0.074 (0.059 to 0.090)	-0.023
	35-39	456 (349 to 565)	0.129 (0.099 to 0.160)	559 (449 to 677)	0.100 (0.080 to 0.121)	-0.029
	40-44	542 (421 to 656)	0.189 (0.147 to 0.229)	739 (610 to 882)	0.148 (0.122 to 0.176)	-0.041
	45-49	616 (490 to 742)	0.265 (0.211 to 0.319)	870 (732 to 1061)	0.184 (0.155 to 0.224)	-0.081
	50-54	870 (711 to 1030)	0.409 (0.334 to 0.484)	1151 (959 to 1368)	0.259 (0.215 to 0.308)	-0.150
	55-59	1293 (1084 to 1486)	0.698 (0.585 to 0.803)	1705 (1423 to 2028)	0.431 (0.360 to 0.513)	-0.267
	60-64	1821 (1584 to 2029)	1.134 (0.986 to 1.263)	2188 (1924 to 2532)	0.684 (0.601 to 0.791)	-0.450
	65-69	2452 (2155 to 2695)	1.984 (1.743 to 2.180)	2876 (2450 to 3318)	1.043 (0.888 to 1.203)	-0.941
	70-74	2705 (2394 to 2955)	3.195 (2.828 to 3.491)	3539 (3059 to 4067)	1.719 (1.486 to 1.976)	-1.476
	75-79	3736 (3374 to 4026)	6.069 (5.482 to 6.541)	4147 (3599 to 4763)	3.145 (2.729 to 3.612)	-2.924
	80-84	3567 (3125 to 3898)	10.084 (8.833 to 11.017)	5008 (4228 to 5651)	5.718 (4.827 to 6.452)	-4.366
	85-89	2734 (2295 to 3018)	18.090 (15.190 to 19.971)	5586 (4458 to 6313)	12.218 (9.750 to 13.808)	-5.872
	90-94	1381 (1107 to 1568)	32.226 (25.831 to 36.588)	4622 (3489 to 5239)	25.837 (19.504 to 29.284)	-6.389
	95+	465 (346 to 531)	45.663 (33.973 to 52.188)	2422 (1690 to 2820)	44.438 (31.008 to 51.734)	-1.225

**Male**	0-4	..	..	..	..	..
	5-9	..	..	..	..	..
	10-14	..	..	..	..	..
	15-19	102 (73 to 129)	0.039 (0.028 to 0.049)	94 (73 to 126)	0.029 (0.023 to 0.039)	-0.010
	20-24	121 (90 to 148)	0.049 (0.036 to 0.060)	128 (95 to 166)	0.042 (0.031 to 0.055)	-0.007
	25-29	139 (110 to 167)	0.063 (0.049 to 0.075)	160 (121 to 201)	0.054 (0.041 to 0.068)	-0.009
	30-34	167 (135 to 197)	0.086 (0.069 to 0.101)	208 (165 to 260)	0.068 (0.054 to 0.085)	-0.018
	35-39	200 (162 to 241)	0.112 (0.091 to 0.135)	251 (198 to 331)	0.089 (0.070 to 0.117)	-0.023
	40-44	243 (192 to 293)	0.166 (0.131 to 0.200)	357 (281 to 439)	0.142 (0.112 to 0.174)	-0.024
	45-49	259 (208 to 303)	0.219 (0.175 to 0.256)	370 (295 to 468)	0.155 (0.124 to 0.197)	-0.064
	50-54	400 (324 to 467)	0.371 (0.301 to 0.434)	544 (445 to 672)	0.245 (0.201 to 0.303)	-0.126
	55-59	592 (486 to 683)	0.637 (0.524 to 0.735)	792 (666 to 970)	0.407 (0.342 to 0.498)	-0.230
	60-64	824 (698 to 940)	1.049 (0.889 to 1.196)	1056 (883 to 1250)	0.679 (0.568 to 0.804)	-0.370
	65-69	1023 (877 to 1140)	1.784 (1.530 to 1.988)	1336 (1124 to 1559)	1.014 (0.853 to 1.183)	-0.770
	70-74	1043 (896 to 1159)	2.772 (2.381 to 3.080)	1564 (1342 to 1831)	1.622 (1.392 to 1.900)	-1.150
	75-79	1271 (1139 to 1390)	5.035 (4.514 to 5.509)	1748 (1527 to 2032)	2.924 (2.554 to 3.398)	-2.111
	80-84	1088 (977 to 1174)	8.188 (7.358 to 8.842)	1959 (1705 to 2204)	5.345 (4.652 to 6.014)	-2.843
	85-89	711 (627 to 777)	14.043 (12.383 to 15.335)	1913 (1640 to 2112)	11.089 (9.505 to 12.239)	-2.954
	90-94	296 (256 to 323)	23.483 (20.331 to 25.643)	1237 (1014 to 1360)	21.219 (17.392 to 23.336)	-2.264
	95+	81 (65 to 90)	31.017 (24.891 to 34.404)	452 (345 to 505)	29.889 (22.825 to 33.377)	-1.128
**Female**	0-4	..	..	..	..	..
	5-9	..	..	..	..	..
	10-14	..	..	..	..	..
	15-19	162 (92 to 238)	0.063 (0.036 to 0.093)	166 (101 to 227)	0.055 (0.033 to 0.075)	-0.008

	20-24	206 (114 to 303)	0.084 (0.047 to 0.124)	220 (143 to 291)	0.075 (0.049 to 0.099)	-0.009
	25-29	213 (129 to 307)	0.097 (0.059 to 0.139)	216 (144 to 281)	0.074 (0.049 to 0.097)	-0.023
	30-34	208 (134 to 294)	0.109 (0.071 to 0.154)	239 (170 to 327)	0.080 (0.057 to 0.109)	-0.029
	35-39	255 (172 to 354)	0.147 (0.099 to 0.204)	309 (220 to 404)	0.111 (0.079 to 0.146)	-0.036
	40-44	299 (213 to 387)	0.213 (0.152 to 0.276)	382 (282 to 487)	0.154 (0.114 to 0.196)	-0.059
	45-49	357 (259 to 465)	0.314 (0.227 to 0.408)	500 (379 to 636)	0.212 (0.161 to 0.270)	-0.102
	50-54	471 (345 to 583)	0.449 (0.329 to 0.556)	608 (446 to 766)	0.273 (0.200 to 0.344)	-0.176
	55-59	701 (542 to 854)	0.759 (0.587 to 0.925)	913 (660 to 1187)	0.454 (0.328 to 0.590)	-0.305
	60-64	997 (825 to 1162)	1.214 (1.005 to 1.416)	1132 (887 to 1420)	0.688 (0.539 to 0.863)	-0.526
	65-69	1429 (1199 to 1634)	2.156 (1.808 to 2.466)	1540 (1181 to 1934)	1.069 (0.820 to 1.343)	-1.087
	70-74	1662 (1417 to 1876)	3.534 (3.012 to 3.988)	1975 (1544 to 2396)	1.805 (1.410 to 2.190)	-1.729
	75-79	2465 (2176 to 2730)	6.787 (5.991 to 7.516)	2399 (1901 to 3027)	3.328 (2.636 to 4.198)	-3.459
	80-84	2480 (2119 to 2765)	11.224 (9.590 to 12.515)	3049 (2437 to 3644)	5.986 (4.785 to 7.154)	-5.238
	85-89	2022 (1645 to 2261)	20.130 (16.369 to 22.501)	3673 (2781 to 4300)	12.902 (9.767 to 15.103)	-7.228
	90-94	1085 (850 to 1250)	35.863 (28.090 to 41.292)	3385 (2423 to 3930)	28.069 (20.093 to 32.585)	-7.794
	95+	384 (280 to 445)	50.692 (36.961 to 58.674)	1970 (1345 to 2318)	50.023 (34.163 to 58.858)	-0.669

Abbreviations: DMVD, degenerative mitral valve disease; RC, rate change; UI, uncertain interval.

Supplementary Table 9: The DALYs rates and rate change for DMVD at sex and age levels, 1990-2021

		1990		2021		1990 – 2021
Sex	Age	Cases (95% UI)	Rate (95% UI)	Cases (95% UI)	Rate (95% UI)	RC per 100,000 population
Both	0-4	..	..	..	..	..
	5-9	..	..	..	..	..
	10-14	..	..	..	..	..
	15-19	19108 (12509 to 25682)	3.679 (2.408 to 4.944)	18865 (13875 to 23939)	3.023 (2.224 to 3.837)	-0.656
	20-24	22081 (14198 to 29287)	4.487 (2.885 to 5.952)	23515 (17901 to 29799)	3.938 (2.998 to 4.990)	-0.549
	25-29	22096 (15625 to 28842)	4.992 (3.530 to 6.516)	23574 (18096 to 28333)	4.007 (3.076 to 4.816)	-0.985
	30-34	21682 (16198 to 27203)	5.625 (4.203 to 7.058)	25867 (20742 to 31356)	4.279 (3.431 to 5.187)	-1.346
	35-39	24120 (18501 to 29855)	6.847 (5.252 to 8.476)	29594 (23770 to 35824)	5.276 (4.238 to 6.387)	-1.571
	40-44	26082 (20224 to 31465)	9.104 (7.059 to 10.983)	35528 (29356 to 42283)	7.102 (5.868 to 8.452)	-2.002
	45-49	26714 (21293 to 32059)	11.505 (9.170 to 13.807)	37714 (31809 to 45887)	7.965 (6.718 to 9.691)	-3.540
	50-54	33893 (27706 to 39707)	15.945 (13.034 to 18.679)	44863 (37355 to 53086)	10.083 (8.396 to 11.931)	-5.862
	55-59	45332 (38306 to 51884)	24.478 (20.684 to 28.015)	60003 (50320 to 70755)	15.163 (12.716 to 17.880)	-9.315
	60-64	58111 (50733 to 66135)	36.182 (31.588 to 41.178)	71181 (62087 to 82773)	22.241 (19.399 to 25.863)	-13.941
	65-69	71590 (62370 to 83866)	57.917 (50.457 to 67.847)	90964 (78014 to 111071)	32.977 (28.282 to 40.266)	-24.940
	70-74	72482 (62508 to 86925)	85.615 (73.833 to 102.673)	110288 (90964 to 140688)	53.580 (44.192 to 68.349)	-32.035
	75-79	82877 (71225 to 100404)	134.637 (115.708 to 163.111)	108273 (86781 to 138302)	82.097 (65.801 to 104.866)	-52.540
	80-84	62634 (53709 to 76007)	177.052 (151.824 to 214.855)	102144 (84020 to 130415)	116.625 (95.932 to 148.905)	-60.427
	85-89	36772 (31162 to 43524)	243.347 (206.217 to 288.026)	81959 (67488 to 101412)	179.257 (147.606 to 221.803)	-64.090
	90-94	15243 (12688 to 17690)	355.707 (296.095 to 412.813)	52998 (42376 to 62015)	296.253 (236.878 to 346.661)	-59.454
	95+	5059 (4031 to 5878)	496.909 (395.913 to 577.349)	25928 (19890 to 30411)	475.710 (364.938 to 557.976)	-21.199

**Men**	0-4	..	..	..	..	..
	5-9	..	..	..	..	..
	10-14	..	..	..	..	..
	15-19	7382 (5313 to 9395)	2.797 (2.013 to 3.560)	6850 (5265 to 9111)	2.138 (1.644 to 2.844)	-0.659
	20-24	8174 (6077 to 9995)	3.297 (2.451 to 4.031)	8647 (6421 to 11249)	2.850 (2.116 to 3.707)	-0.447
	25-29	8751 (6894 to 10469)	3.933 (3.098 to 4.705)	10029 (7569 to 12623)	3.373 (2.546 to 4.245)	-0.560
	30-34	9675 (7791 to 11440)	4.954 (3.989 to 5.857)	12040 (9545 to 15042)	3.940 (3.124 to 4.923)	-1.014
	35-39	10614 (8599 to 12765)	5.936 (4.810 to 7.139)	13269 (10480 to 17529)	4.688 (3.702 to 6.193)	-1.248
	40-44	11720 (9267 to 14127)	8.013 (6.336 to 9.659)	17179 (13523 to 21077)	6.813 (5.363 to 8.358)	-1.200
	45-49	11287 (9142 to 13195)	9.533 (7.721 to 11.145)	16114 (12945 to 20401)	6.774 (5.442 to 8.577)	-2.759
	50-54	15711 (12950 to 18521)	14.594 (12.029 to 17.204)	21356 (17518 to 26082)	9.621 (7.892 to 11.750)	-4.973
	55-59	21216 (18021 to 24623)	22.841 (19.400 to 26.508)	28495 (24458 to 34783)	14.634 (12.560 to 17.863)	-8.207
	60-64	27547 (23638 to 32060)	35.071 (30.094 to 40.817)	35913 (30595 to 43243)	23.090 (19.671 to 27.803)	-11.981
	65-69	32553 (27295 to 39458)	56.780 (47.610 to 68.825)	46392 (38031 to 58791)	35.190 (28.848 to 44.595)	-21.590
	70-74	32107 (25860 to 40193)	85.348 (68.742 to 106.842)	56840 (43480 to 74701)	58.968 (45.107 to 77.497)	-26.380
	75-79	33879 (27332 to 43521)	134.266 (108.320 to 172.479)	54477 (42027 to 72336)	91.119 (70.295 to 120.990)	-43.147
	80-84	23494 (19070 to 30477)	176.867 (143.561 to 229.433)	48317 (37513 to 64390)	131.826 (102.350 to 175.681)	-45.041
	85-89	11925 (9883 to 15147)	235.485 (195.165 to 299.112)	34040 (27303 to 44089)	197.303 (158.256 to 255.550)	-38.182
	90-54	4069 (3374 to 4976)	323.210 (267.968 to 395.209)	17390 (14370 to 21402)	298.355 (246.540 to 367.190)	-24.855
	95+	1193 (970 to 1436)	458.491 (372.581 to 551.833)	6566 (5254 to 7803)	434.278 (347.465 to 516.077)	-24.213
**Women**	0-4	..	..	..	..	..
	5-9	..	..	..	..	..
	10-14	..	..	..	..	..
	15-19	11727 (6644 to 17301)	4.589 (2.600 to 6.771)	12015 (7298 to 16455)	3.957 (2.404 to 5.419)	-0.632

	20-24	13907 (7714 to 20517)	5.696 (3.160 to 8.404)	14868 (9647 to 19679)	5.061 (3.284 to 6.699)	-0.635
	25-29	13345 (8098 to 19269)	6.063 (3.679 to 8.755)	13545 (9008 to 17652)	4.655 (3.096 to 6.066)	-1.408
	30-34	12007 (7753 to 16967)	6.316 (4.078 to 8.925)	13828 (9834 to 18880)	4.626 (3.290 to 6.316)	-1.690
	35-39	13506 (9099 to 18681)	7.786 (5.246 to 10.770)	16325 (11650 to 21353)	5.877 (4.193 to 7.686)	-1.909
	40-44	14362 (10239 to 18564)	10.242 (7.302 to 13.239)	18349 (13564 to 23329)	7.396 (5.467 to 9.403)	-2.846
	45-49	15427 (11211 to 20056)	13.556 (9.851 to 17.623)	21601 (16440 to 27432)	9.167 (6.977 to 11.641)	-4.389
	50-54	18183 (13375 to 22472)	17.331 (12.748 to 21.419)	23507 (17274 to 29446)	10.544 (7.748 to 13.208)	-6.787
	55-59	24116 (18725 to 29363)	26.125 (20.285 to 31.808)	31508 (22682 to 40920)	15.675 (11.284 to 20.358)	-10.450
	60-64	30564 (25561 to 35673)	37.244 (31.148 to 43.471)	35268 (27843 to 44024)	21.438 (16.924 to 26.761)	-15.806
	65-69	39038 (33371 to 45395)	58.900 (50.351 to 68.492)	44572 (35767 to 55028)	30.951 (24.836 to 38.211)	-27.949
	70-74	40376 (34842 to 47667)	85.828 (74.065 to 101.328)	53448 (43107 to 68184)	48.834 (39.386 to 62.299)	-36.994
	75-79	48998 (42739 to 57898)	134.895 (117.666 to 159.398)	53796 (44317 to 68881)	74.615 (61.467 to 95.538)	-60.280
	80-84	39140 (33943 to 46062)	177.164 (153.641 to 208.498)	53827 (43935 to 66970)	105.687 (86.263 to 131.492)	-71.477
	85-89	24847 (20932 to 28733)	247.310 (208.337 to 285.984)	47919 (37995 to 57677)	168.320 (133.462 to 202.594)	-78.990
	90-54	11174 (9201 to 12816)	369.226 (304.031 to 423.503)	35608 (27465 to 41418)	295.237 (227.719 to 343.409)	-73.989
	95+	3866 (3021 to 4476)	510.101 (398.559 to 590.650)	19361 (14500 to 22766)	491.617 (368.176 to 578.071)	-18.484

Abbreviations: DALYs, disability-adjusted life years; DMVD, degenerative mitral valve disease; RC, rate change; UI, uncertain interval.

Supplementary Table 10: Decomposition analysis of DMVD prevalence, mortality and DALYs at global and SDI levels, 1990-2021

Prevalence changes due to population-level determinants			
	**Aging**	**Population**	**EC**
Global	3832623.206 (45.72%)	5282439.424 (63.01%)	-732172.380 (-8.73%)
High SDI	2143881.146 (50.10%)	1632256.493 (38.14%)	502980.700 (11.75%)
High-middle SDI	1453298.719 (64.93%)	996881.701 (44.54%)	-211783.059 (-9.46%)
Middle SDI	709165.894 (49.52%)	637225.578 (44.49%)	85788.842 (5.99%)
Low-middle SDI	97416.531 (26.98%)	256801.180 (71.12%)	6839.123 (1.89%)
Low SDI	-4301.235 (-6.51%)	68731.613 (103.99%)	1664.535 (2.52%)
**Mortality changes due to population-level determinants**			
	**Aging**	**Population**	**EC**
Global	12569.709 (97.52%)	15197.354 (117.91%)	-14877.846 (-115.43%)
High SDI	7863.735 (241.43%)	4302.088 (132.08%)	-8908.700 (-273.51%)
High-middle SDI	2872.401 (117.54%)	1744.051 (71.36%)	-2172.600 (-88.90%)
Middle SDI	1895.147 (63.47%)	2009.043 (67.28%)	-918.315 (-30.76%)
Low-middle SDI	767.142 (25.27%)	2476.606 (81.59%)	-208.393 (-6.87%)
Low SDI	-49.945 (-4.37%)	1666.401 (145.80%)	-473.543 (-41.43%)
**DALYs changes due to population-level determinants**			
	**Aging**	**Population**	**EC**
Global	230595.744 (77.54%)	396151.843 (133.21%)	-329365.966 (-110.76%)
High SDI	127397.315 (264.87%)	90891.042 (188.97%)	-170190.087 (-353.84%)
High-middle SDI	64464.806 (127.50%)	49378.754 (97.66%)	-63281.767 (-125.16%)

Middle SDI	44199.227 (56.12%)	66063.481 (83.88%)	-31507.343 (-40.01%)
Low-middle SDI	14984.542 (18.09%)	79045.730 (95.44%)	-11205.108 (-13.53%)
Low SDI	-1577.580 (-4.30%)	55506.022 (151.25%)	-17229.084 (-46.95%)

Abbreviations: DALYs, disability-adjusted life years; DMVD, degenerative mitral valve disease; EC, epidemiology changes; SDI, sociodemographic index.

Supplementary Table 11: Predicted prevalence, mortality and DALYs of DMVD from 2021 to 2035 reported by 5-year intervals at global level

	2025		2030		2035	
	**Cases (95% UI)**	**Age-standardized per 100,000 population (95% UI)**	**Cases (95% UI)**	**Age-standardized per 100,000 population (95% UI)**	**Cases (95% UI)**	**Age-standardized per 100,000 population (95% UI)**
Prevalence	17328153 (16062047,18594258)	178.211 (167.981,188.441)	19374065 (16419721,22328409)	170.250 (145.522,194.979)	21410812 (15718776,27102848)	161.499 (119.519,203.479)
Mortality	39540 (35791,43288)	0.437 (0.372,0.502)	43084 (33407,52761)	0.405 (0.304,0.506)	47878 (28449,67307)	0.379 (0.224,0.534)
DALYs	1014410 (933592,1095229)	10.86 (9.994,11.730)	1101629 (893361,1309896)	10.367 (8.399,12.335)	1204730 (793487,1615972)	10.009 (6.572,13.446)

Abbreviations: DALYs, disability-adjusted life years; DMVD, degenerative mitral valve disease; UI, uncertain interval.

Supplementary Figure 1: Map showing age-standardized rates and average annual percentage change in global mortality of DMVD, 1990-2021

a

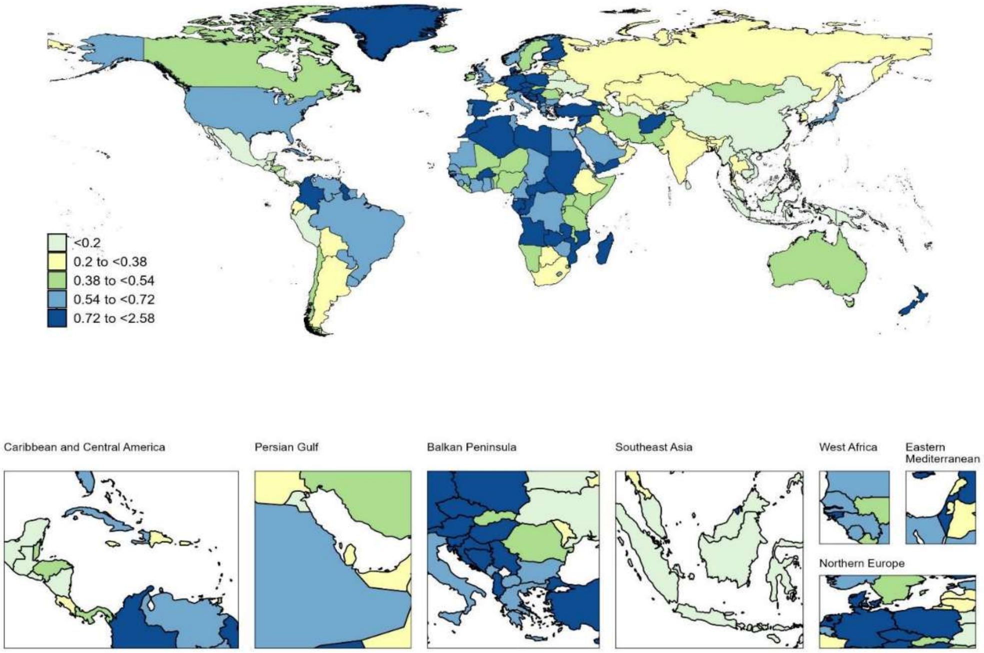

b

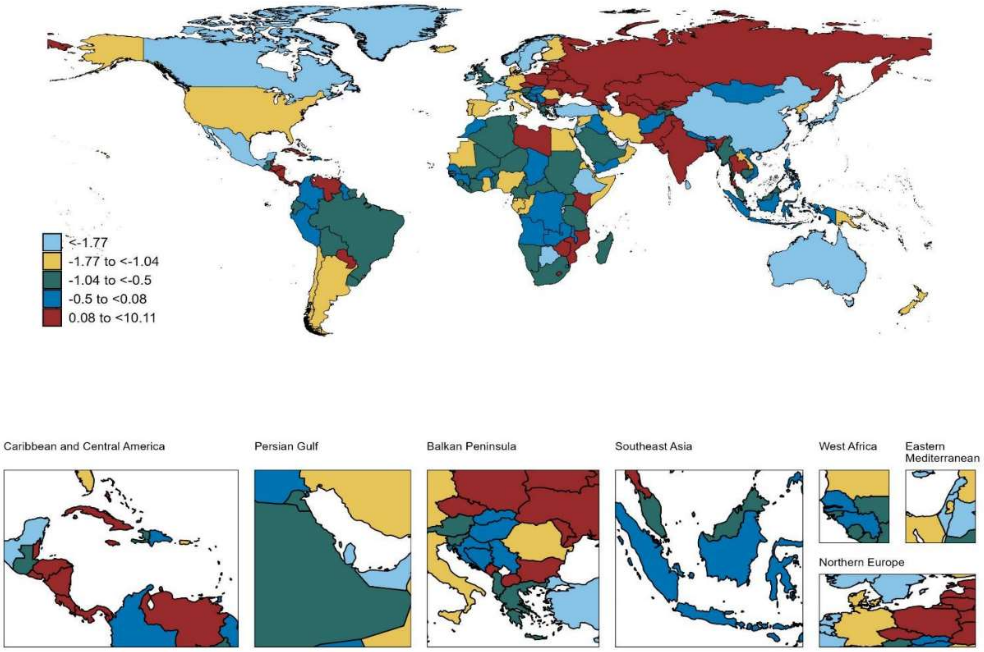

(a) Age-standardized mortality rates in 2021; (b) Average annual percent changes of age-standardized mortality rates from 1990 to 2021. Abbreviations: DMVD, degenerative mitral valve disease.

Supplementary Figure 2: Map showing age-standardized rates and average annual percentage change in global DALYs of DMVD, 1990-2021

a

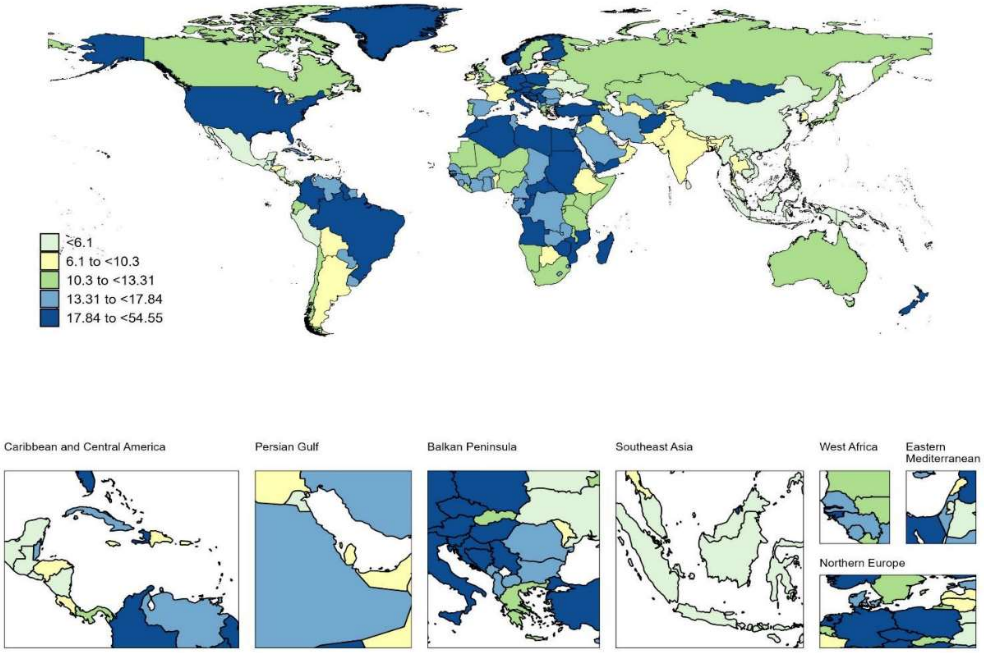

b

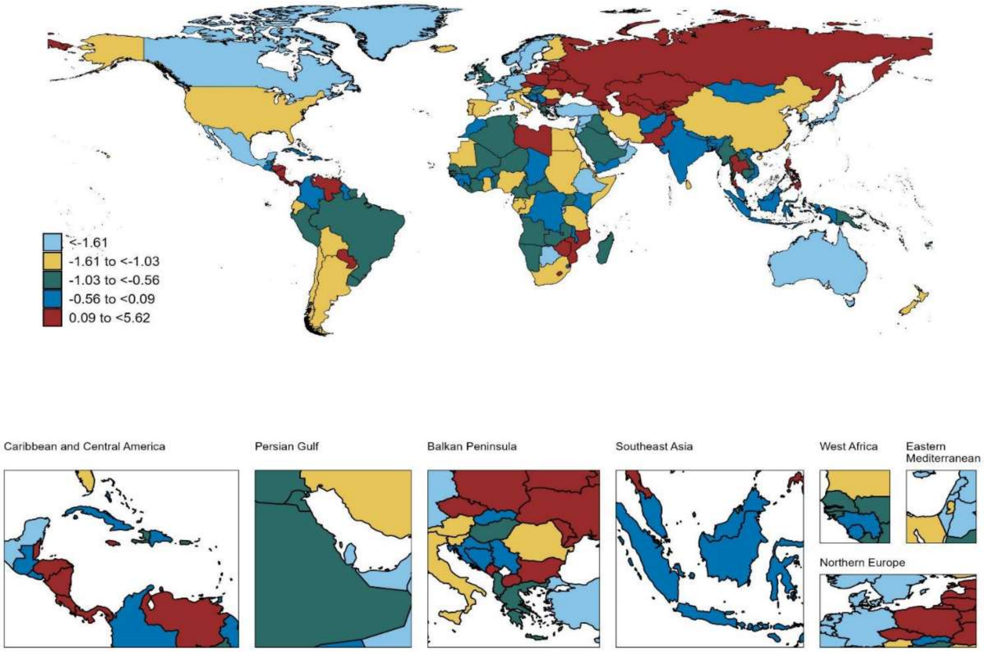

(a) Age-standardized DALYs rates in 2021; (b) Average annual percent changes of age-standardized DALYs rates from 1990 to 2021. Abbreviations: DMVD, degenerative mitral valve disease; DALYs, disability-adjusted life years.

Supplementary Figure 3: Age-standardized prevalence, mortality and DALYs rate of DMVD at SDI and sex levels, 1990-2021

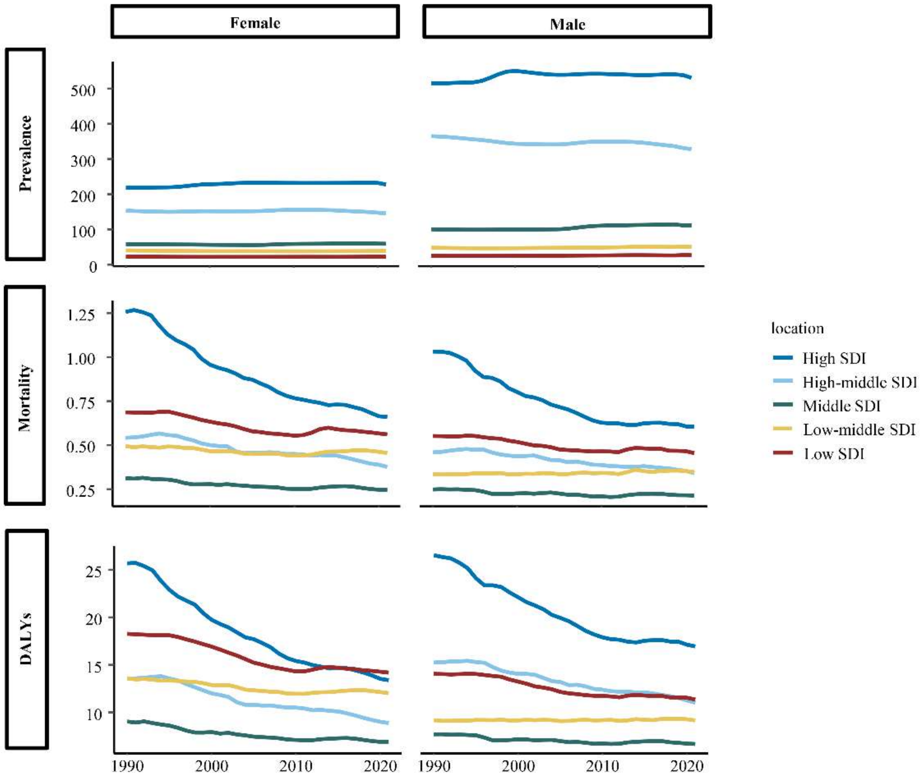

Abbreviations: DALYs, disability-adjusted life years; DMVD, degenerative mitral valve disease; SDI, sociodemographic index.

Supplementary Figure 4: Average annual percent changes of age-standardized rates of DMVD at SDI and sex levels, 1990-2021

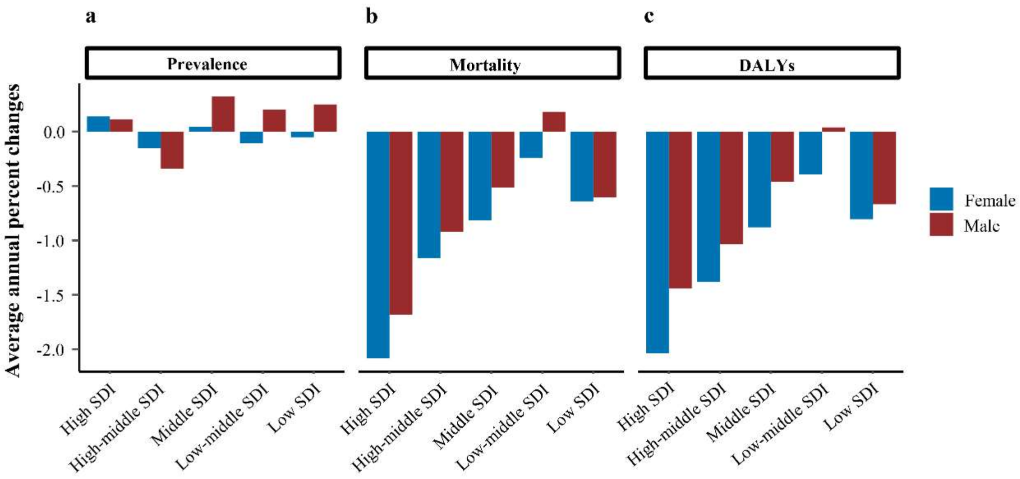

(a) Average annual percent changes of age-standardized prevalence rates from 1990 to 2021; (b) Average annual percent changes of age-standardized mortality rates from 1990 to 2021; (c) Average annual percent changes of age-standardized DALYs rates from 1990 to 2021. Abbreviations: DALYs, disability-adjusted life years; DMVD, degenerative mitral valve disease; SDI, sociodemographic index.

Supplementary Figure 5: Rate change in prevalence, mortality and DALYs of DMVD at sex and age levels, 1990-2021

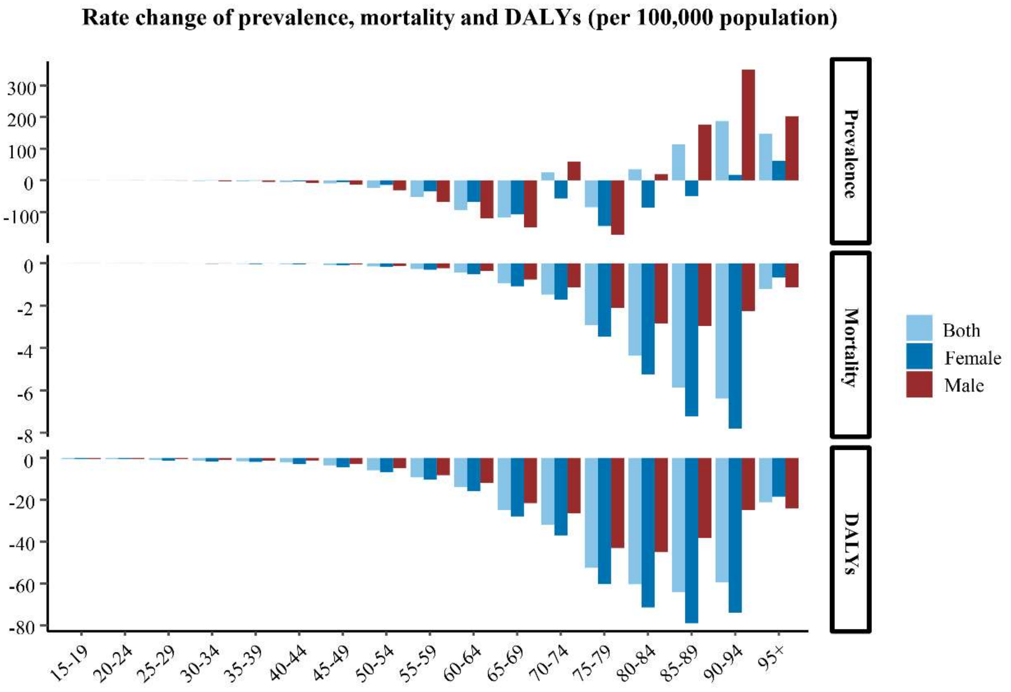

Abbreviations: DALYs, disability-adjusted life years; DMVD, degenerative mitral valve disease; SDI, sociodemographic index.

Supplementary Figure 6: Rate and rate change in prevalence of DMVD at SDI and age levels, 1990-2021

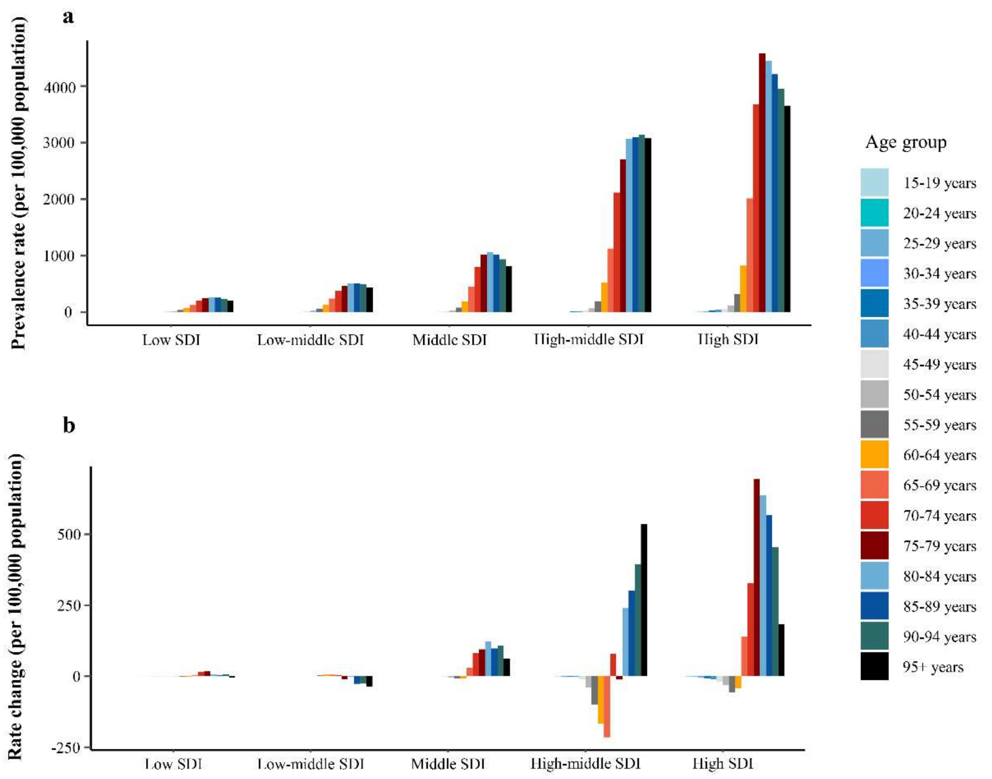

(a) Prevalence rate of DMVD in 2021; (b) Rate change of prevalence for DMVD from 1990 to 2021; Abbreviations: DMVD, degenerative mitral valve disease; SDI, sociodemographic index.

Supplementary Figure 7: Rate and rate change in mortality of DMVD at SDI and age levels, 1990-2021

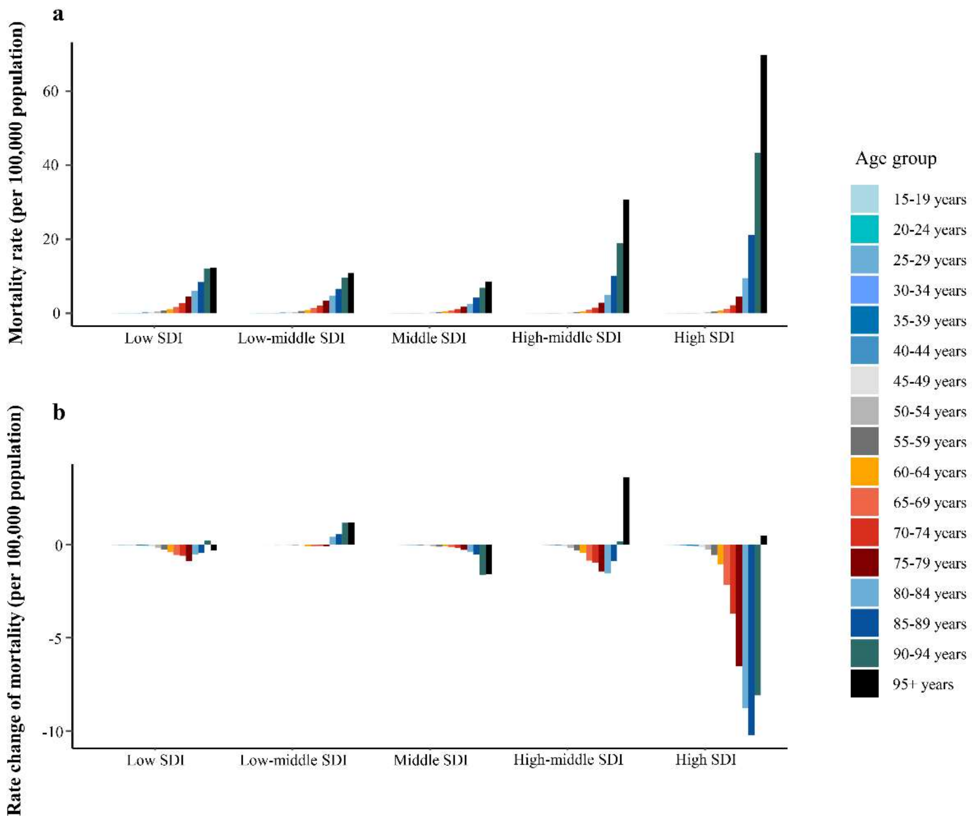

(a) Mortality rate of DMVD in 2021; (b) Rate change of mortality for DMVD from 1990 to 2021; Abbreviations: DMVD, degenerative mitral valve disease; SDI, sociodemographic index.

Supplementary Figure 8: Rate and rate change in DALYs of DMVD at SDI and age levels, 1990-2021

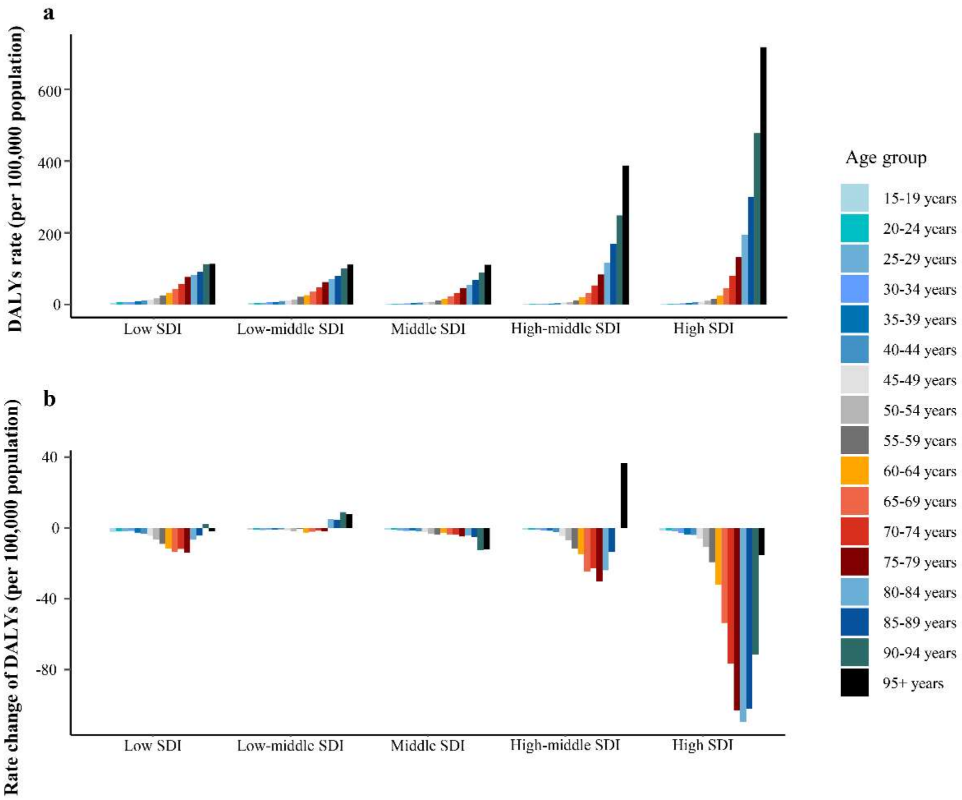

(a) DALYs rate of DMVD in 2021; (b) Rate change of DALYs for DMVD from 1990 to 2021; Abbreviations: DALYs, disability-adjusted life years; DMVD, degenerative mitral valve disease; SDI, sociodemographic index.
